# Benefit-Risk Trade-offs and Patient Preferences for Therapy Selection in Ulcerative Colitis: a Multicountry Preference Study

**DOI:** 10.1093/ibd/izae162

**Published:** 2024-08-10

**Authors:** Javier P Gisbert, Stefan Schreiber, Corey A Siegel, Fernando Magro, Anna Jus, Chiara Whichello, Christine Michaels-Igbokwe, Sebastian Heidenreich, Alessandra Oortwijn, Séverine Vermeire

**Affiliations:** Gastroenterology Unit, Hospital Universitario de La Princesa, Instituto de Investigación Sanitaria Princesa (IIS-Princesa), Universidad Autónoma de Madrid (UAM), Madrid, Spain; Centro de Investigación Biomédica en Red de Enfermedades Hepáticas y Digestivas (CIBERehd), Carlos III Health Institute, Madrid, Spain; Department of Internal Medicine I, Kiel University, University Hospital Schleswig-Holstein, Kiel, Germany; Inflammatory Bowel Disease Center, Section of Gastroenterology & Hepatology, Dartmouth-Hitchcock Medical Center, Lebanon, NH, USA; The Dartmouth Institute for Health Policy and Clinical Practice, Geisel School of Medicine at Dartmouth, Hanover, NH, USA; CINTESIS@RISE, Faculty of Medicine, University of Porto, Porto, Portugal; Galapagos NV, Leiden, Netherlands; Evidera Inc., London, UK; Evidera Inc., Montreal, QC, Canada; Evidera Inc., London, UK; Galapagos NV, Leiden, Netherlands; Department of Gastroenterology and Hepatology, UZ Leuven, Leuven, Belgium

**Keywords:** benefit-risk assessment, filgotinib, ulcerative colitis

## Abstract

**Background:**

To help navigate the complex treatment landscape of ulcerative colitis (UC), we quantified the benefit-risk trade-offs that patients were willing to make when choosing treatment.

**Methods:**

Patients completed an online discrete choice experiment. Eligible patients had a UC diagnosis for ≥6 months, were aged ≥18 years, and resided in France, Germany, Italy, Spain, or the UK. Patients chose between 2 hypothetical treatments set up to ensure trade-offs were made. Clinical trial data, literature review, and patient interviews identified treatment attributes. Relative attribute importance (RAI) scores and maximum acceptable risks were generated. A patient-centric benefit-risk assessment of 200 mg of filgotinib was conducted as an example to show how measured trade-offs can be used.

**Results:**

Overall, 631 patients participated; patients had a mean age of 42.2 years and were predominantly male (75.3%). Achieving and maintaining clinical remission was the most important factor for patients (RAI 32.4%); to achieve this, patients were willing to accept slightly higher risks of blood clots, serious infections, and malignancies compared with lower risk treatment profiles. Patients also valued the convenience of oral treatments, avoiding steroids, and the ability to attend school/work. The patient-centric benefit-risk assessment suggested patients are significantly more likely to prefer Janus kinase 1 preferential inhibitor filgotinib over placebo.

**Conclusions:**

Achieving clinical remission was the highest treatment priority for patients. To attain this, patients were willing to accept some slightly higher risk treatment profiles. Patient choices in the benefit-risk assessment suggested patients were significantly more likely to prefer filgotinib over placebo.

**Key messages**

**What is already known?** The UC treatment landscape is becoming increasingly complex; a variety of therapies are available with diverse efficacy, safety, and dosing profiles.

**What is new here?** Achieving and maintaining clinical remission was considered the most important factor for patients when choosing a treatment for UC; to achieve this, patients were willing to accept slightly higher risks of blood clots, serious infections, and malignancies compared with lower risk treatment profiles.

**How can this study help patient care?** Understanding patient treatment preferences can optimize shared decision-making when choosing a treatment in clinical practice and help ensure patients with UC have access to the appropriate therapies to fulfil their needs.

## Introduction

Ulcerative colitis (UC) is a chronic, idiopathic inflammatory disorder of the colonic mucosa that affects the rectum and typically expands to the more proximal parts of the colon.^1,2^ The disease is characterized by symptoms of abdominal pain, rectal bleeding, mucus discharge, bowel urgency, incontinence, and frequent bowel movements.^2^ Extraintestinal disease manifestations can affect multiple bodily systems, including the musculoskeletal, dermatologic, hepato-pancreato-biliary, ocular, metabolic, and renal systems.^3^

Ulcerative colitis can impair patients’ health-related quality of life (HRQoL); patients may perceive social stigmatization, combined with high levels of unemployment, sick leave, and the need for disability benefits.^4,5^ Ulcerative colitis disease activity is commonly assessed by stool frequency and rectal bleeding using the Mayo Clinic score.^6^ However, patient HRQoL can be negatively affected by a vast range of other gastrointestinal and extraintestinal symptoms, including fatigue and psychological factors.^5,7,8^

There is currently no cure for UC; the aim of treatment is to induce and maintain disease remission and preserve HRQoL.^9,10^ The UC treatment landscape is becoming increasingly complex, with a variety of treatment options available with diverse efficacy, safety, and dosing profiles,^11^ and the potential for combination therapies.^12^ Despite the range of therapeutic options, approximately one-half to two-thirds of patients do not achieve clinical remission, and patients often require dose escalation during maintenance therapy.^13–16^

Conventional UC treatments include 5-aminosalicylates, oral or topical corticosteroids, and immunosuppressants.^9,17^ In the case of refractory disease or drug intolerance, advanced therapies including biologics and small molecules are used.^17,18^ These agents target specific inflammatory mediators and include, but are not limited to, tumor necrosis factor-α antagonists, integrin receptors antagonists, sphingosine 1-phosphate receptor modulators, and interleukin antagonists.^19–21^ Small-molecule Janus kinase (JAK) inhibitors are orally administered treatments that simultaneously block multiple cytokines from different inflammatory pathways.^22^ JAK inhibitors that are approved for the treatment of UC include filgotinib, tofacitinib, and upadacitinib.^23–25^

The treatment strategy for UC is typically based on disease severity, distribution, and the pattern of disease (eg, relapse frequency, disease course, and previous medications).^26^ However, a treat-to-target strategy has recently been advocated by the Selecting Therapeutic Targets Inflammatory Bowel Disease (STRIDE) program, which considers the restoration of patient QoL as the ultimate long-term treatment target.^27,28^ Furthermore, a recent Delphi consensus panel recommended using a multicomponent assessment that goes beyond traditional trial end points to capture aspects that are important to patients; the panel suggested comprehensive disease control should be a treatment target in clinical practice.^29^ These strategies require the availability of patient preference data that can be used to derive patient-centric treatment plans, in which the relative importance of treatment risks and benefits to patients are considered. Patient preferences can be elicited using discrete choice experiments (DCE).^30^ A DCE uses quantitative methodology whereby patients are asked to make a choice between hypothetical healthcare options described by a common set of attributes that are systematically varied to ensure trade-offs are made.^31^

Although some patient preference studies in inflammatory bowel disease (IBD) have been conducted,^32–34^ there remains a need to align treatment preferences with clinical end points to conduct a truly patient-centric benefit-risk assessment (BRA), particularly with regards to advanced therapies. Here, we quantified the benefit-risk trade-offs that patients with UC were willing to accept when selecting a treatment. We applied the elicited patient preference information from the DCE to a patient-centric BRA of the JAK1 preferential inhibitor filgotinib.^25^

## Materials and Methods

### Study Design

A mixed-methods approach with 3 phases was used to design and test an online survey, which was composed of DCE and best-worst scaling (BWS) instruments (see Supplementary Figure 1, which summarizes the study design).

A targeted review of published UC literature and clinical trial data identified patient-relevant benefits, risks, and nonclinical aspects of UC treatments as treatment attributes for further discussion in interviews with patients (see Supplementary Text 1, which details the literature review methodology and see Supplementary Tables 1-2, which summarize the benefits and risks of medical treatments for UC extracted from evidence review). Qualitative 60-minute, virtual interviews were subsequently conducted with 25 patients across France, Germany, Italy, Spain, or the UK (5 patients from each country; see Supplementary Text 2, which details the qualitative interviews). The results were used to refine the list of patient-relevant treatment attributes to be included in the DCE and identify the key impacts of UC on patient HRQoL for use in the BWS (see Supplementary Figure 2, which summarizes the treatment themes in a conceptual map).

### Discrete Choice Experiment

**Figure 1.**
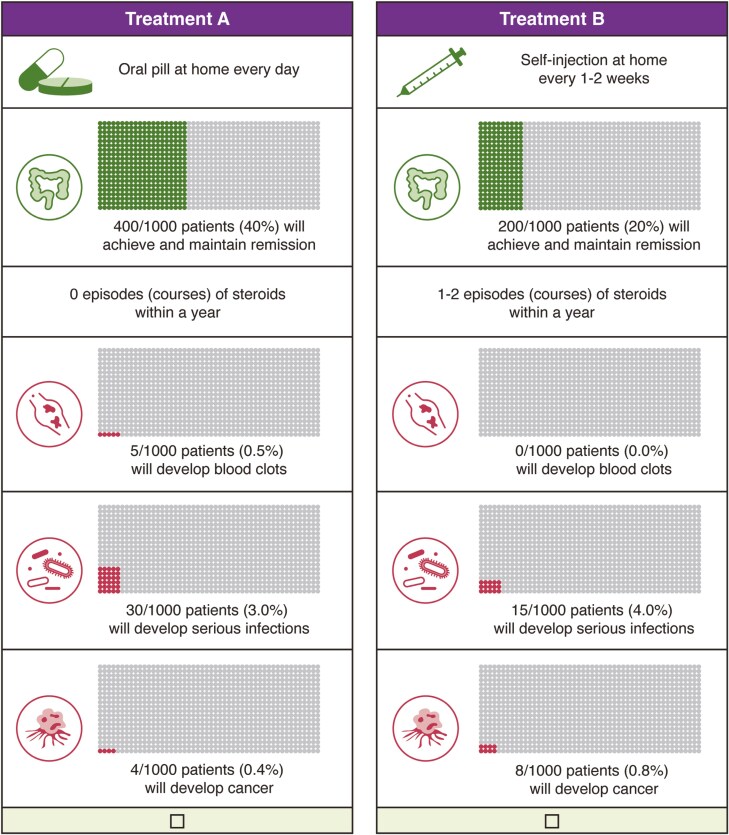
Example of a final discrete choice experiment task.

The most important attributes selected for inclusion in the DCE and corresponding levels are reported in Table 1; these include how and how often the treatment is taken, likelihood of achieving and maintaining remission, steroid use, and risk of blood clots, serious infections, and malignancies. These attributes and levels were used to generate DCE choice tasks. For each DCE choice task, patients were asked to choose between 2 hypothetical treatment options. The presentation order of tasks was randomized between patients, and attribute levels were systematically varied to ensure patients had to make trade-offs. An example DCE choice task is shown in Figure 1. A D-efficient design with 30 choice tasks was generated using Ngene version 1.2 (ChoiceMetrics, Sydney, Australia) and split equally across 3 blocks.^35^ In addition, 3 nonexperimental tasks were added to the DCE to test the internal validity of the data: a practice choice task to familiarize patients with the DCE format, a repeated choice task to test the stability of patient preferences, and a dominance task to test compensatory preferences.

**Table 1.** Attributes and corresponding levels included in the discrete choice experiment.

Attribute description	Definition	Levels
How and how often the treatment is taken	How the treatment is taken can differ between medicines. Whereas some medicines are taken every day via an oral pill, others may require regular injections every 8 weeks. Some injections can be administered at home, and others need to be injected by a clinician in a hospital.	Intravenous treatment in hospital or clinic every 4 to 8 weeks. Self-injection at home every 1 to 2 weeks. Oral pill at home every day.
Likelihood of achieving and maintaining remission	UC treatments may help you achieve and maintain remission. Remission means that you have no rectal bleeding and a stool frequency that’s close to normal.	20% (200 of 1000 patients) 40% (400 of 1000 patients) 60% (600 of 1000 patients)
Number of episodes (courses) of steroids per year	Steroids are often used to treat the UC symptoms, in combination with other therapies. One episode refers to the use of steroids for 3 months or less. Two episodes refers to separate courses of steroid use of 3 months or less, separated by a period of no steroid use in between. Each episode of steroid use can result in weight gain, mood swings, acne, sleep problems, facial hair growth, or concentration problems.	No steroid use 1 to 2 episodes (courses) 3 to 4 episodes (courses)
Risk of blood clots	UC patients have an increased risk of blood clots that can also be affected by treatments. Blood clots are a medical emergency, are often treated with blood-thinning medication, and may require hospitalization. In some cases, a blood clot can travel to the lung or heart, which can cause severe complications and may be life-threatening.	0.0% (0 of 1000 patients) 0.5% (5 of 1000 patients) 1.0% (10 of 1000 patients)
Risk of serious infections	Some UC treatments may make patients more susceptible to serious infections. Serious infections, such as pneumonia, require hospitalization and may become life-threatening. Different treatments have different risks of causing serious infections.	0.0% (0 of 1000 patients) 1.5% (15 of 1000 patients) 3.0% (30 of 1000 patients)
Risk of cancer	Some UC treatments may make patients more susceptible to cancers/malignancies. Some cancers can be treated or cured with treatment, while others may not be treatable.	0.0% (0 of 1000 patients) 0.4% (4 of 1000 patients) 0.8% (8 of 1000 patients)

Abbreviation: UC, ulcerative colitis.

### Best-worst Scaling

A BWS (Case 1) exercise was used to understand the HRQoL benefits that patients would like to achieve from their treatment, over and above remission (see **Supplementary Table 3**, which presents the full set of 8 HRQoL benefits considered in this study). A balanced incomplete block design was used to generate 14 BWS questions that were split into 2 equally sized blocks. In each BWS question, patients were asked to identify the best and the worst out of 4 HRQoL benefits.^36^ Patients were randomly assigned to one block.

In addition to the DCE and BWS instruments, the survey included clinical and sociodemographic questions and data quality indicators, including a health literacy and health numeracy questionnaire (where low scores indicate low health literacy/numeracy).

To pretest the DCE and BWS instruments, 25 qualitative pilot interviews (5 patients from each country, as noted above) were conducted using a cognitive interviewing approach in which patients were asked to “think aloud” while completing the survey.^37^ The qualitative interviews were used to gather feedback on the patient usability of the instruments and determine the patient’s ability and willingness to make benefit-risk trade-offs in the DCE. Patient feedback was used to improve survey presentation and wording, including the risk communication approach. Changes made to the survey following qualitative pilot testing included editorial changes of definitions and attribute levels, addition of background information on advanced therapies, improvement to visualizations, refinement of patient-friendly medical terms, and updates to attribute icons. A quantitative pilot study was then conducted with 81 patients (France *n* = 4, Germany *n* = 4, Italy *n* = 16, Spain *n* = 39, and the UK *n* = 18) to assess if the DCE attribute levels sufficiently covered the relevant patient preference ranges and to ascertain expected data quality. No changes were made to the attribute levels following the quantitative pilot study; however, the experimental design was updated based on Bayesian priors derived from the pilot study.

The final survey was completed by patients between June 9 and June 22, 2022, to elicit UC treatment preferences. Eligible patients had a diagnosis of UC for ≥6 months, were aged ≥18 years, and resided in France, Germany, Italy, Spain, or the UK. Patients were excluded if they had a diagnosis of infectious colitis, Crohn’s disease, or irritable bowel syndrome or had undergone a colectomy, ileostomy, or J-pouch surgery. Patients were recruited via databases, commercially managed online access panels, social media, and patient associations. Informed consent was provided by all patients before participation in the study. The study protocol received central ethical approval from Ethical & Independent Review Services, a fully accredited institutional review board.

### Statistical Analysis

Descriptive analyses were conducted on the final data set to assess data quality indicators including, but not limited to, health literacy, numeracy, stability and dominance tests, and response time.

A correlated mixed logit model was used to analyze the DCE data within a random utility maximization framework that estimated the effect of changes in attributes on preferences as part-worth utilities. The model’s statistical performance was assessed based on the Bayesian information criterion, adjusted McFadden pseudo-R^2^ (APR), and/or log-likelihood ratio test.

Multiple behavioral output measures were obtained from the DCE data. Relative attribute importance (RAI) scores were calculated to measure the maximal contribution of each attribute to a preference, conditional on that attribute’s level range. Standard errors (SEs) and 95% confidence intervals (CIs) were obtained for the RAI scores using a parametric bootstrap. The mixed logit model was used to estimate the interaction effects between attributes and particular characteristics to obtain subgroup-specific RAI scores; such characteristics included country, sex at birth, age, time since diagnosis, and treatment experience with biologics and/or JAK inhibitors. Maximum acceptable risks (MARs) of blood clots, serious infections, and malignancies were estimated to capture average trade-offs that patients were willing to make between treatment-related risk and benefit attributes.

To illustrate how DCE estimates can be used to inform a patient-centric BRA, the predicted probabilities of patients preferring 200 mg of filgotinib over placebo were obtained through a combination of estimated patient preference weights and clinical data from the SELECTION phase 2b/3 clinical trial (see Supplementary Table 4, which reports the values and clinical data sources used to describe 200 mg of filgotinib and placebo treatment profiles).^38^ Deterministic sensitivity analyses were performed to account for any differences/uncertainty in incidence rates of adverse events (AEs) between filgotinib and placebo.

Best-worst scaling data were analyzed in a pairwise MaxDiff multinomial logit model, and 95% CIs were obtained.

Please see Supplementary Text 3 and Supplementary Table 5 for additional information on the statistical analyses used in this study.

## Results

### Patient Demographics and Clinical Characteristics

Survey invitation emails were sent to 8997 patients, of whom 4240 patients were screened. Following the screening process, 834 patients were considered eligible to participate in the survey; and of these, 631 completed the survey (see Supplementary Figure 3, which provides full details of patient disposition). In total, 631 patients with UC across France (*n* = 110), Germany (*n* = 107), Italy (*n* = 146), Spain (*n* = 142), and the UK (*n *= 126) completed the survey.

Patient demographics and clinical characteristics are presented in Table 2. Overall, patients had a mean age of 42.2 years, were mostly male (*n* = 475 [75.3%]), educated to college/university level (*n* = 301 [47.7%]), employed full-time (*n* = 354 [56.1%]), and had public health insurance (*n* = 377 [59.7%]). Most patients had high health literacy (*n* = 400 [63.4%]) and high health numeracy (*n* = 617 [97.8%]). Nearly half of patients (*n* = 294 [46.6%]) were diagnosed with UC more than 2 years before completing the survey; the remaining patients (*n* = 337 [53.4%]) were diagnosed between 6 months and 2 years before completing the survey. Patients reported receiving 5-aminosalicylates (*n* = 412 [65.3%]), immunomodulators (*n* = 406 [64.3%]), steroids (*n* = 354 [56.1%]), biologics (*n* = 281 [44.5%]), and JAK inhibitors (*n* = 9 [1.4%]) to treat UC in the 12 months preceding the survey. In the week leading up to the survey, patients reported rectal bleeding (*n *= 559 [88.6%]) and abnormal stool frequency (*n* = 583 [92.4%]).

**Table 2.** Patient demographics and clinical characteristics.

	Overall (*N* = 631, 100%)	UK (*n* = 126, 20%)	France (*n* = 110, 17%)	Germany (*n* = 107, 17%)	Italy (*n* = 146, 23%)	Spain (*n* = 142, 23%)
**Age, years**						
Mean (SD)	42.2 (7.7)	44.0 (9.5)	42.6 (6.4)	41.8 (6.3)	42.2 (8.0)	40.5 (7.3)
Min, max	22, 77	22, 77	22, 65	27, 62	24, 75	24, 65
**Gender**						
Male	475 (75)	85 (67)	86 (78)	94 (88)	104 (71)	106 (75)
Female	156 (25)	41 (33)	24 (22)	13 (12)	42 (29)	36 (25)
**Education**						
Elementary school	3 (0)	0 (0)	0 (0)	0 (0)	3 (2)	0 (0)
High school	85 (13)	13 (10)	13 (12)	12 (11)	36 (25)	11 (8)
College/university (BA, BSc)	301 (48)	71 (56)	53 (48)	55 (51)	47 (32)	75 (53)
Postgraduate (Master, MD, PhD)	188 (30)	26 (21)	44 (40)	24 (22)	59 (40)	35 (25)
Other	54 (9)	16 (13)	0 (0)	16 (15)	1 (1)	21 (15)
**Employment status**						
Employed, full-time	354 (56)	76 (60)	40 (36)	46 (43)	105 (72)	87 (61)
Employed, part-time	190 (30)	31 (25)	54 (49)	53 (50)	19 (13)	33 (23)
Self-employed	28 (4)	3 (2)	1 (1)	2 (2)	14 (10)	8 (6)
Unemployed	11 (2)	5 (4)	0 (0)	0 (0)	2 (1)	4 (3)
Retired	16 (3)	5 (4)	7 (6)	2 (2)	2 (1)	0 (0)
On sick leave	20 (3)	3 (2)	7 (6)	3 (3)	0 (0)	7 (5)
Other	12 (2)	3 (2)	1 (1)	1 (1)	4 (3)	3 (2)
**Insurance status**						
Private insurance	335 (53)	59 (54)	58 (54)	81 (55)	59 (42)	78 (62)
Public insurance	377 (60)	56 (51)	49 (46)	100 (68)	105 (74)	67 (53)
**Health literacy**						
Low	231 (37)	35 (28)	45 (41)	48 (45)	45 (31)	58 (41)
High	400 (63)	91 (72)	65 (59)	59 (55)	101 (69)	84 (59)
**Numeracy**						
Low	14 (2)	3 (2)	0 (0)	2 (2)	7 (5)	2 (1)
High	617 (98)	123 (98)	110 (100)	105 (98)	139 (95)	140 (99)
**Time since UC diagnosis**						
6 months-1 year ago	106 (17)	24 (19)	21 (19)	24 (22)	24 (16)	13 (9)
1-2 years ago	231 (37)	42 (33)	59 (54)	37 (35)	49 (34)	44 (31)
2-5 years ago	212 (34)	39 (31)	22 (20)	40 (37)	46 (32)	65 (46)
>5 years ago	82 (13)	21 (17)	8 (7)	6 (6)	27 (18)	20 (14)
**Health comorbidities**						
Allergies	22 (3)	5 (4)	1 (1)	2 (2)	9 (6)	5 (4)
Arthritis	13 (2)	6 (5)	0 (0)	1 (1)	3 (2)	3 (2)
Cancer	1 (0)	0 (0)	0 (0)	0 (0)	1 (1)	0 (0)
Diabetes	20 (3)	2 (2)	3 (3)	1 (1)	9 (6)	5 (4)
Heart disease	5 (1)	1 (1)	1 (1)	2 (2)	0 (0)	1 (1)
Hyperlipidemia	3 (0)	0 (0)	0 (0)	0 (0)	3 (2)	0 (0)
Hypertension	35 (6)	12 (10)	3 (3)	4 (4)	5 (3)	11 (8)
Stroke	2 (0)	2 (2)	0 (0)	0 (0)	0 (0)	0 (0)
**Bowel movements when in remission or before diagnosis or symptom onset**						
Mean (SD)	2.9 (1.7)	2.9 (1.9)	2.9 (1.6)	3.3 (1.8)	2.6 (1.6)	2.7 (1.4)
Min, max	1, 15	1, 15	1, 11	1, 12	1, 11	1, 8
Median (Q1, Q3)	2 (2, 4)	3 (2, 3)	3 (2, 4)	3 (2, 4)	2 (2, 3)	2 (2, 3)
**Stool frequency in past week**						
Normal	48 (8)	16 (13)	4 (4)	5 (5)	13 (9)	10 (7)
1-2 additional stools per day	273 (43)	54 (43)	45 (41)	32 (30)	69 (47)	73 (51)
3-4 additional stools per day	265 (42)	48 (38)	46 (42)	58 (54)	58 (40)	55 (39)
>4 additional stools per day	45 (7)	8 (6)	15 (14)	12 (11)	6 (4)	4 (3)
**Rectal bleeding in past week**						
None	72 (11)	24 (19)	9 (8)	12 (11)	11 (8)	16 (11)
In stool, half the time	407 (65)	71 (56)	84 (76)	70 (65)	85 (58)	97 (68)
In stool, half the time or more	141 (22)	30 (24)	15 (14)	24 (22)	44 (30)	28 (20)
Passing blood alone	11 (2)	1 (1)	2 (2)	1 (1)	6 (4)	1 (1)
**Treatment taken in past 12 months**						
Steroids^a^	354 (56)	66 (52)	53 (48)	66 (62)	88 (60)	81 (57)
5-aminosalicylates^b^	412 (65)	100 (79)	73 (66)	87 (81)	64 (44)	88 (62)
Immunomodulators^c^	406 (64)	87 (69)	90 (82)	61 (57)	68 (47)	100 (70)
Biologics^d^	281 (45)	57 (45)	50 (45)	35 (33)	70 (48)	69 (49)
Janus kinase inhibitors^e^	9 (1)	3 (2)	1 (1)	3 (3)	1 (1)	1 (1)

All data are *n* (%), unless otherwise stated.

Abbreviations: max, maximum; min, minimum; SD, standard deviation; Q1, first quartile; Q3, third quartile.

^a^Steroids (overall): oral steroids, 182 (29); intravenous steroids, 180 (29); rectal suppository or foam/cream steroids, 147 (23).

^b^5-aminosalicylates (overall): mesalamine/mesalazine, 375 (59); sulfasalazine, 43 (7).

^c^Immunomodulators (overall): azathioprine, 108 (17); 6-mercaptopurine/mercaptopurine, 78 (12); methotrexate, 144 (23); cyclosporine/ciclosporin, 84 (13); tacrolimus, 36 (6).

^d^Biologics (overall): adalimumab, 58 (9); ustekinumab, 38 (6); vedolizumab, 60 (10); infliximab, 81 (13); golimumab, 44 (7); certolizumab, 45 (7).

^e^Janus kinase inhibitors (overall): tofacitinib, 9 (1).

### Preference Elicitation

**Figure 2.**
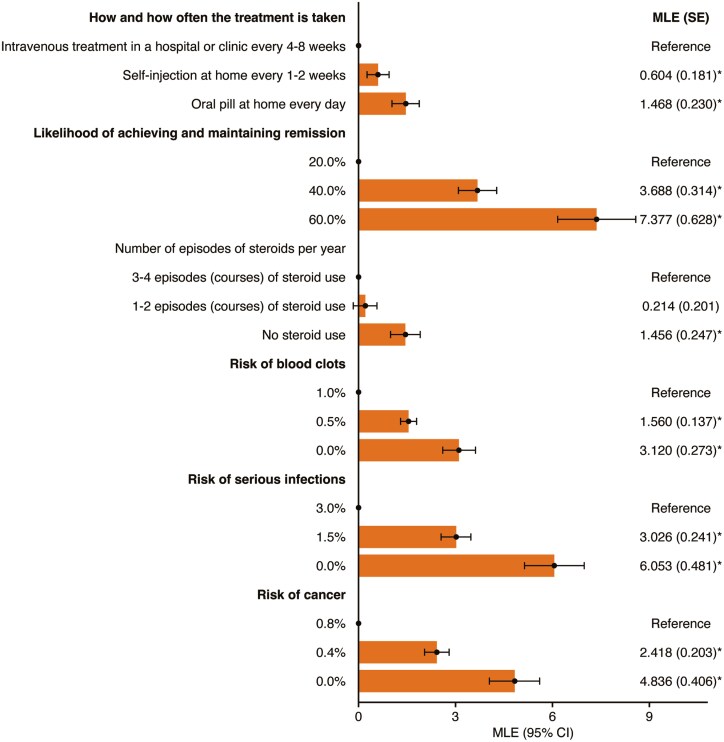
Maximum likelihood estimates from the discrete choice experiment. *Statistically significant (*P < *.001). Abbreviations: BIC, Bayesian information criterion; MLE, maximum likelihood estimate; SE, standard error. Constant: 0.167 (0.112); log-likelihood at convergence: -969.7; BIC: 2411.9; adjusted McFadden pseudo-R^2^: 76.6%; total number of parameters: 54. Estimates are marginal utilities that denote the effect of deviating from a reference level of an attribute on patient preferences.

Overall, 545 patients (86.4%) made consistent choices in the stability test, and 598 patients (94.8%) passed the dominance test. The model had a good APR data fit of 76.6%. The maximum likelihood estimates from the DCE are presented in Figure 2.

All else being equal, patients were more likely to choose a treatment taken as a daily oral pill (*P < *.001) or a subcutaneous self-injection at home every 1 to 2 weeks (*P < *.001) over an intravenously (i.v.) administered treatment in a hospital/clinic every 4 to 8 weeks (Figure 2). A daily oral pill at home was preferred over a subcutaneous self-injection at home every 1 to 2 weeks (*P = *.003). Patients preferred a treatment that would offer a 60% likelihood of achieving and maintaining remission over a 40% (*P < *.001) and 20% (*P < *.001) likelihood of achieving and maintaining remission. Patients also preferred a treatment that avoided the use of steroids over taking steroids (*P < *.001), although a preference was not distinguished between 1 to 2 and 3 to 4 courses per year (*P > *.05). In addition, patients preferred a 0% risk of cancer, blood clots, and serious infections over higher risks (0.8, 1.0, and 3.0%, respectively; all *P < *.001; Figure 2).

Maintaining and achieving remission (RAI, 32.4%; 95% CI, 29.5-35.2) was the most important driver of patient preference for UC treatment, and mode of treatment administration was the least important (RAI, 6.4%; 95% CI, 4.8-8.0). Reducing the risk of serious infections (RAI, 26.6%; 95% CI, 24.5-28.6) and malignancies (RAI, 21.2%; 95% CI, 19.3-23.2) were 3.9 and 3.1 times more important to patients than reducing the risk of blood clots (RAI, 6.9%; 95% CI, 5.9-7.8), respectively (see Supplementary Figure 4, which reports RAI scores for the overall population).

**Figure 3.**
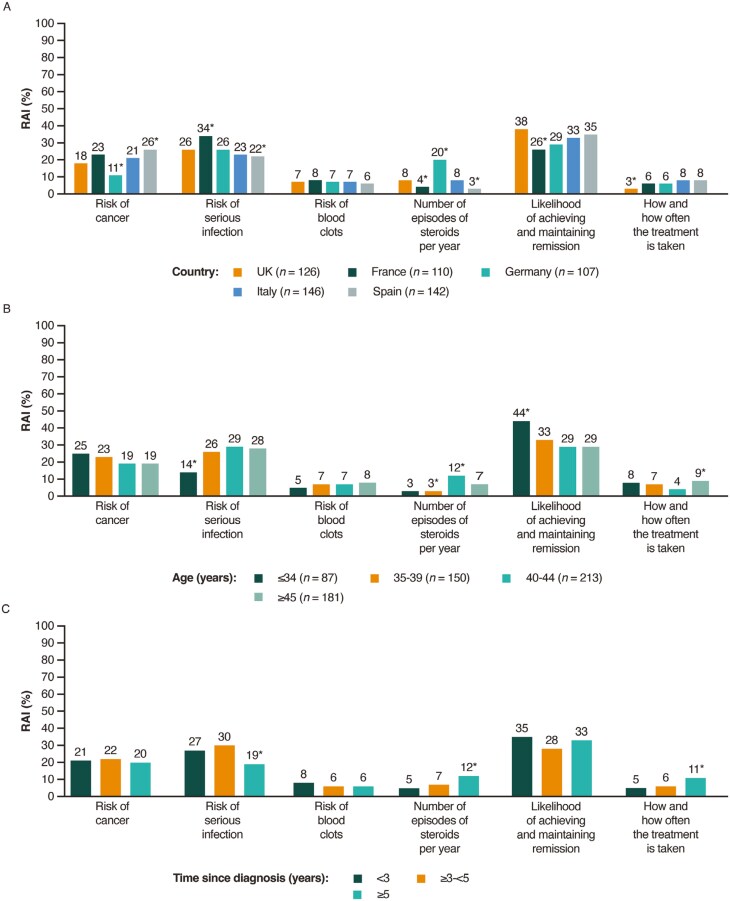

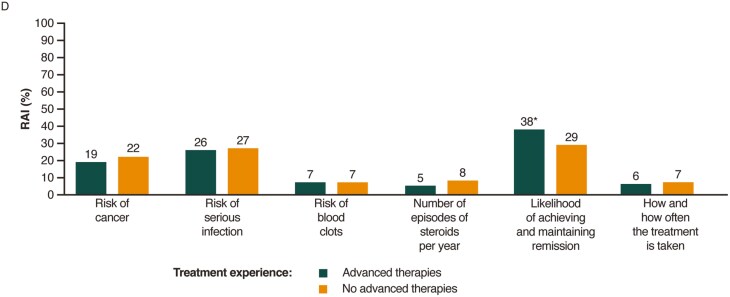
Relative attribute importance by (A) country, (B) age group, (C) time since diagnosis, and (D) treatment experience. Only RAI >3% are displayed. *Statistically significant from the corresponding overall sample RAI scores at the 5% confidence level. Abbreviation: RAI, relative attribute importance.

Subgroup analyses of RAI scores showed treatment preferences and priorities varied significantly (*P < *.05) by country of residence, age, treatment experience, and time since diagnosis (see Figure 3A-D, which present RAI data stratified by subgroup). When choosing a treatment, patients in France considered the most important factor to be risk of serious infections, in Spain it was risk of malignancies, and in Germany it was steroid use (all *P < *.01; Figure 3A). Achieving and maintaining remission was more important to patients aged 39 years and younger and those with previous exposure to advanced therapies (ie, JAK inhibitors or biologics) compared with patients aged 40 years and older and those who had never received an advanced therapy, respectively (both *P < *.05; Figure 3B and 3D). Patients who received a diagnosis of UC 5 years ago or more placed less importance on the risk of serious infections, but a greater importance on avoiding the use of steroids and having a more convenient mode of administration compared with patients diagnosed with UC more recently (less than 5 years ago; Figure 3C).

### Trade-offs

The MAR estimates capture the average trade-offs that patients with UC were willing to make between the risk of blood clots, serious infections, and malignancies. The results are presented in **Table 3**.

**Table 3.** MARs of serious infections, malignancies, or blood clots.

Attribute change		MAR of blood clots,^a^ % (95% CI)	MAR of serious infections,^b^ % (95% CI)	MAR of malignancies,^c^ % (95% CI)
From	To			
**Administration route**				
i.v.	SQ	0.2 (0.2-0.3)^*^	0.3 (0.2-0.4)^*^	0.2 (0.1-0.2)^*^
i.v.	Oral	0.7 (0.6-0.8)^*^	0.8 (0.7-0.9)^*^	0.2 (0.2-0.3)^*^
SQ	Oral	0.4 (0.3-0.6)	0.5 (0.4-0.5)^*^	0.1 (0.1-0.1)
**Likelihood of achieving and maintaining remission**				
20.0%	40.0%	1.4 (1.2-1.6)^*^	2.0 (1.7-2.3)^*^	0.7 (0.6-0.8)^*^
40.0%	60.0%	1.4 (1.2-1.6)	2.0 (1.7-2.3)	0.7 (0.6-0.8)
20.0%	60.0%	2.7 (2.3-3.2)^*^	4.0 (3.4-4.6)^*^	1.5 (1.2-1.7)^*^
**Risk of blood clots**				
0.5%	0.0%	–	0.7 (0.5-0.8)	0.3 (0.2-0.4)
1.0%	0.5%	–	0.7 (0.5-0.8)^*^	0.3 (0.2-0.4)^*^
1.0%	0.0%	–	1.3 (1.1-1.6)^*^	0.6 (0.5-0.7)^*^
**Risk of malignancies**				
0.4%	0.0%	0.9 (0.7-1.0)	1.2 (1.1-1.4)	–
0.8%	0.4%	0.9 (0.7-1.0)^*^	1.2 (1.1-1.4)^*^	–
0.8%	0.0%	1.8 (1.5-2.0)^*^	2.4 (2.2-2.7)^*^	–
**Risk of serious infections**				
1.5%	0.0%	1.1 (0.9-1.2)	–	0.6 (0.5-0.6)
3.0%	1.5%	1.1 (0.9-1.2)^*^	–	0.6 (0.5-0.6)^*^
3.0%	0.0%	2.2 (1.9-2.5)^*^	–	1.1 (1.0-1.3)^*^

^*^Statistically significant (*P < *.001).

^a^Constant: 0.017 (0.007); log-likelihood at convergence: -2148.7; BIC: 4770.0; adjusted McFadden pseudo-R^2^: 49.6%.

^b^Constant: 0.028 (0.010); log-likelihood at convergence: -1708.7; BIC: 3889.9; adjusted McFadden pseudo-R^2^: 59.7%.

^c^Constant: 0.002 (0.009); log-likelihood at convergence: -1785.8; BIC: 4044.1; adjusted McFadden pseudo-R^2^: 57.9%.

Abbreviations: BIC, Bayesian information criterion; CI, confidence interval; i.v., intravenous; MAR, maximum acceptable risk; SQ, self-injection.

Total number of parameters: 54; estimates denote risk equivalences of an attribute change. Equal MARs for changes of the same size in the same attribute result from linear effects.

Despite patient preference for a 0% risk of blood clots, serious infections, and malignancies over higher risks (all *P* < .001; Figure 2), MAR estimates indicated that patients were willing to accept a treatment with a higher AE risk if it were associated with an increased likelihood of achieving/maintaining remission. For a 20% increased likelihood of remission (from 20.0% to 40.0% and 40.0% to 60.0%), on average, patients were willing to accept an increase in risk of blood clots (1.4%; 95% CI, 1.2%-1.6%), serious infections (2.0%; 95% CI, 1.7%-2.3%), and malignancies (0.7%; 95% CI, 0.6%-0.8%). Similarly, for improved convenience in treatment administration, patients were willing to accept a higher AE risk. For example, on average, patients were willing to accept an increase in the risk of blood clots (0.7%; 95% CI, 0.6%-0.8%), serious infections (0.8%; 95% CI, 0.7%-0.9%), or malignancies (0.2%; 95% CI, 0.2%-0.3%) to have an oral pill once a day instead of an i.v. treatment (in a hospital or clinic) every 4 to 8 weeks.

For a 0.5% reduction in the risk of blood clots (0.5%-0.0% and 1.0%-0.5%), on average, patients were willing to tolerate an increase in risk of serious infections (0.7%; 95% CI, 0.5%-0.8%) and malignancies (0.3%; 95% CI, 0.2%-0.4%). For a 0.4% reduction in the risk of malignancies, patients were willing to tolerate an increase in risk of serious infections (1.2%; 95% CI, 1.1%-1.4%) and blood clots (0.9%; 95% CI, 0.7%-1.0%). For a 1.5% reduction in the risk of serious infections, patients were willing to tolerate an increase in risk of malignancies (0.6%; 95% CI, 0.5%-0.6%) and blood clots (1.1%; 95% CI, 0.9%-1.2%).

### Best-worst Scaling Data

Health-related quality of life benefits were scaled in order of importance, with a score of 100 assigned to the attribute ranked most important and a score of 0 for the attribute ranked least important; these scores were relative and not absolute (see Supplementary Figure 5, which presents the BWS data). Overall, patients placed the highest importance on improvements in their ability to attend school or work (relative weight [RW], 100.0). Following this, a similar importance (*P* > .05) was placed on improvement in their ability to perform daily activities (RW, 75.0; 95% CI, 66-84), the patient’s overall energy level (RW, 71.1; 95% CI, 62-80), reduced worry and anxiety related to UC (RW, 70.6; 95% CI, 62-79), and a good night’s sleep (RW, 65.9; 95% CI, 57-75). A treatment that would improve patients’ ability to attend social engagements was ranked the next most important (RW, 38.8; 95% CI, 30-47), followed by a treatment that would improve patients’ ability to maintain healthy sexual relationships (RW, 12.9; 95% CI, 4-22). Patients placed the lowest relative importance on improvements in participation in sports and leisure activities (RW, 0.0; reference category).

Health-related quality of life priorities were found to differ across countries of residence, age groups, and time since diagnosis (see Supplementary Figure 6, which presents the BWS data stratified by subgroups). Patients aged 35 years or older placed the greatest importance on improvements in attendance at school or work, and those aged 34 years or younger placed the greatest importance on achieving a good night’s sleep. Patients who had received a UC diagnosis between 2 and 5 years ago placed the most importance on improvements in feelings of worry and/or anxiety related to UC. In comparison, patients who received a diagnosis between 6 months and 1 year ago placed the most importance on improvement in overall energy levels.

### Treatment Profile Comparison

The preference estimates from the DCE data analysis were used to compare a treatment profile with clinical characteristics equivalent to that of 200 mg of filgotinib with placebo (see Supplementary Table 4, which reports the values and clinical data sources used to describe 200 mg of filgotinib and placebo treatment profiles).

**Figure 4.**
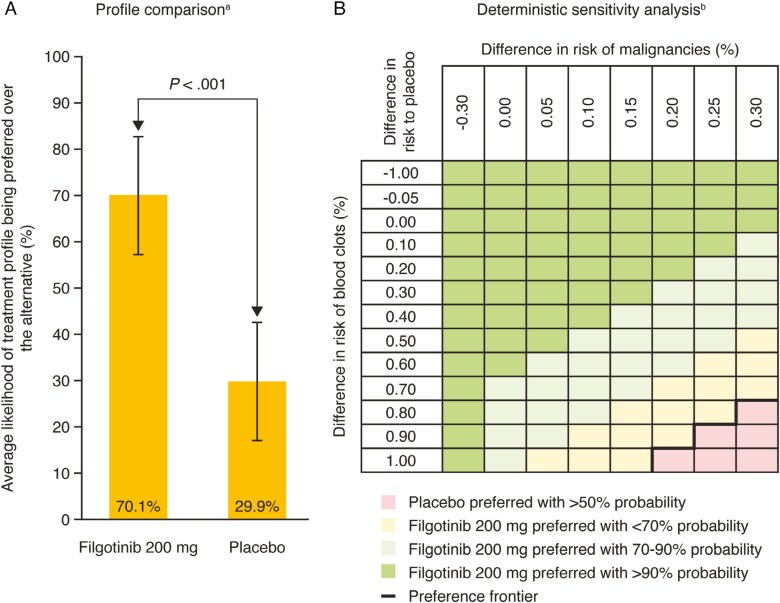
Patient preference for the treatment profile of 200 mg of filgotinib vs placebo. A, treatment profile comparison; B, deterministic sensitivity analysis. Abbreviation: AE, adverse event. ^a^Estimates denote the average likelihood of one treatment profile being preferred over the alternative. ^b^The grid shows the effect of differences in risks of malignancies and serious infections on the likelihood of 200 mg of filgotinib being preferred over placebo. A positive risk difference means filgotinib increases the net risk of the AE. A negative risk difference means that filgotinib decreases the net risk of the AE. Placebo was preferred over 200 mg of filgotinib for all combinations of risk differences under the preference frontier.

The treatment profile comparison denotes the average likelihood of a profile being preferred over the alternative. The results suggested that 200 mg of filgotinib has a 70.1% probability of being preferred over placebo (Figure 4A). A deterministic sensitivity analysis was conducted by varying risks of blood clots (−1.00 to 1.00%) and malignancies (−0.30 to 0.30%). In more than 90% of these scenarios, patients were more likely to prefer 200 mg of filgotinib over placebo (Figure 4B). Placebo was preferred over filgotinib with more than 50% probability in scenarios where the risks of blood clots were 0.80%, 0.90%, and 1.00% in combination with risks of malignancies of 0.30%, 0.25% to 0.30%, and 0.20% to 0.30%, respectively.

## Discussion

Here, we present knowledge that will help optimize treat-to-target approaches for UC and shared decision-making in clinical practice. We identified patient treatment preferences to quantify the benefit-risk trade-offs that patients with UC may make when choosing between therapeutic strategies.

When choosing a treatment, achieving and maintaining remission is considered an important factor to patients with UC.^34,39^ To increase their likelihood of achieving remission by 20%, patients were willing to accept a slight increase in the risk of blood clots, malignancies, and serious infections.

Following the achievement of remission, patients with UC consider avoiding treatment-related risks as the next most important factor during treatment selection. In particular, the risks of serious infections and malignancies were considered 3.9 and 3.1 times more important to the patient than the risk of blood clots, respectively. When choosing a treatment, the importance of certain risks appeared to change with age. Patients aged 34 years or younger placed the greatest importance on the risk of malignancies; the importance placed on this risk decreased with increasing age. Conversely, the risk of serious infections was considered significantly less important to patients aged 34 years or younger compared with patients aged 35 and older; the importance placed on this risk increased with increasing age. These findings align with a study conducted on lay awareness of the relationship between age and disease, in which older age was associated with a lower perceived cancer risk and a higher perceived disease risk than younger age.^40^ The heterogeneity observed across age groups emphasizes the value of patient-centric BRAs and highlights the need to balance the safety and efficacy needs of both the physician and patient in shared treatment decision-making.

These RAI observations are clinically relevant because patients with UC have an increased risk of malignancies,^41^ major adverse cardiovascular events,^42^ and venous thromboembolisms^43^ compared with the general population. Although JAK inhibitors have also been associated with increased risks of malignancies, major adverse cardiovascular events, and venous thromboembolisms when compared with anti-TNFs among patients ≥65 years old/or with cardiovascular risk factors,^44–46^ a recent integrated safety analysis of filgotinib in patients with UC suggested that these events occur at a low frequency in patients treated with 200 mg of filgotinib in general, including among patients ≥65 years.^47^ Patient perception of these risks would have been highly dependent on how the information was presented in the survey; the risk definitions are provided in Table 1. To convey the potential severity of blood clots and serious infections, these were defined in the survey as a “medical emergency” and “life-threatening,” respectively. Furthermore, given the timing of the study, it is believed that the COVID-19 vaccine coverage would have improved the general population’s understanding of blood clot risks.

Although the route of administration was considered least important by patients compared with the risk of AEs, steroid use, and clinical remission, patients preferred oral administration (*P* < .001) or subcutaneous self-injection of UC treatment at home (*P* < .001) over an i.v. treatment in the hospital. This highlights the relevance of convenient treatment strategies for patients. To achieve convenience in treatment administration (specifically, a once-daily oral pill), patients were willing to accept a 0.2% to 0.8% increase in the risk of AEs. The convenience of treatment was considered significantly more important by patients who received a UC diagnosis more than 5 years ago than by those who received a UC diagnosis less than 5 years ago.

While patients valued being able to avoid steroids, a simple reduction in frequency of steroid use did not impact patients’ treatment choices in the DCE. Additionally, the RAI of steroid use ranked below remission and the risk of AEs. This aligns with previous preference data for patients with UC.^33^ However, given patients significantly preferred avoiding the use of steroids, this study supports steroid-free remission as a relevant end point in clinical research. In line with this, a Delphi consensus panel recently recommended that corticosteroid-free remission is important to consider when assessing comprehensive disease control in UC.^29^ Of the 5 countries analyzed, patients in Germany were the most concerned about the need for steroids. It can be hypothesized that this may be owing to local awareness campaigns regarding steroid use; however, this country-specific interpretation is limited by the small number of patients enrolled from each country.

Patients with UC considered attending work and school as the most important treatment goal, beyond clinical remission. The markedly negative impact of UC on patients’ work and school life is well-documented.^48,49^ The ability to participate in leisure activities and to maintain sexual relationships were ranked least important when assessing HRQoL; this result is perhaps not surprising given UC has been reported to impact basic life activities, such as work and sleep. The high proportion of male participants in this study could also provide a possible explanation for the low importance placed on maintaining sexual relationships, because the literature reports reduced sexual activity as being more common among female patients with UC than male patients.^50,51^ The male population was unintentionally over-represented in this study at 75%, despite employing a range of recruitment methods, including healthcare professional referrals, online panels, social media, and patient associations. Health-related quality of life priorities were found to differ across countries of residence, within age groups, and based on time since diagnosis. Restoration of HRQoL is an important long-term treatment target for patients with UC,^52,53^ and a better understanding of patients’ HRQoL treatment goals may contribute to the use of patient-relevant end points in future clinical trials.

The strengths of this study include the large patient population. More than 600 patients with UC from 5 different countries were included; therefore, the study considers preferences from a wide group of patients. The instrument design was informed by best practice mixed-methods research and included attributes and levels that align with clinically relevant end points and the filgotinib safety profile.^38^ This allowed the data to be used for patient-centric BRAs. Internal validity tests indicated high data quality for this study; a large proportion of patients made consistent choices in the stability test (86%) and passed the dominance test (95%). These results are superior to the typical rates observed in comparable healthcare DCEs in the literature; for example, stability test pass rates of 70% and 74% and dominance test pass rates of 78% and 85% were reported in 2 recent DCE studies.^54,55^ The model also had a good APR data fit of 76.6%, suggesting that it was able to explain the choices that patients made in the DCE.

Limitations of the study include potential selection bias arising from data being available only for patients who chose to participate in the study (631 of 8997 patients), potentially resulting in a patient population who were more motivated to achieve disease remission than the general UC patient population. In addition, over 60% of patients in this study had high health literacy; further research is needed to understand the relationship between health literacy and UC treatment preference. As with all DCE patient preference studies, hypothetical bias could affect how applicable the results are to real-world clinical decisions. Patients responding to the questionnaire may not be fully representative of the general IBD population, owing to the oversampling of male patients in the main sample. Additionally, this patient population may have more severe/complex forms of UC than the general IBD population, with a high proportion of patients requiring the use of immunosuppressants and biologic agents. Data were not recorded on duration of remission at the time of study completion; remission was recognized during the study by an attribute, patient-friendly definition (Table 1). The impact of remission on benefit-risk decision-making when choosing treatment was therefore not assessed. Patient preference data should not be extrapolated to individual patients; patient life experiences, location, healthcare system, treatment cost, and healthcare professionals’ treatment experiences all influence the available treatment options and the associated benefit-risk analyses.

Patient preference data from the DCE, clinical trial data,^38^ and published literature were combined to generate a treatment profile comparable to 200 mg of filgotinib. The phase 2b/3 clinical trial (SELECTION) demonstrated that 200 mg of filgotinib was well tolerated and effective in inducing and maintaining clinical remission of UC when compared with placebo. Despite the random nature of patient preference data, patients were likely to prefer 200 mg of filgotinib over a placebo comparator about 70% of the time. The aforementioned increased risks of malignancies and major adverse cardiovascular events associated with JAK inhibitors when compared with other UC treatments or placebo are likely to have influenced patient choice.^44–46^ However, when the rate of blood clot and malignancy risk were varied, patients are expected to prefer 200 mg of filgotinib over placebo in more than 90% of scenarios. These data suggest patients are likely to consider that the benefits offered by filgotinib outweigh the risks. This provides an example of how patient-centric BRAs can be derived from patient preference data and clinical data to inform decision-making; application of these data to a wider range of therapies for UC would be useful.

Understanding patient treatment preferences for UC can help ensure patients have access to the appropriate therapies to fulfill their needs, contributing to increased treatment satisfaction, improved compliance/adherence, and shared decision-making. The choices made by patients in the DCE implied that their treatment preferences were mostly driven by the likelihood of achieving and maintaining remission. Patients also valued convenience in treatment administration and avoiding the use of steroids. Although the avoidance of treatment-related risks was important to patients, they were willing to accept some increase in the risks of blood clots, serious infections, and malignancies to increase the chance of clinical remission; these trade-off data can further support shared treatment decision-making when treatment for UC is considered among patients. A patient-centric BRA generated from these patient preference data and clinical data provided further context to the clinical data from the SELECTION trial.^38^ The heterogeneity in treatment preferences observed across patient subgroups emphasizes the value of involving patients in shared decision-making and treat-to-target approaches to design a tailored treatment plan that aligns with patients’ individual treatment priorities.

## Supplementary Data

Supplementary data is available at *Inflammatory Bowel Diseases* online.

izae162_suppl_Supplementary_Material
