# Supplementary material for: Benefit-Risk Trade-offs and Patient Preferences for Therapy Selection in Ulcerative Colitis: a Multicountry Preference Study
Source: Inflamm Bowel Dis. 2024 Aug 10;31(5):1281–94. doi: 10.1093/ibd/izae162 (PMC12069987; doi:10.1093/ibd/izae162)
Supplement: izae162_suppl_Supplementary_Material [file izae162_suppl_supplementary_material.docx]

1. **Supplementary Text** **1.** Literature review methodology.

A targeted literature review considered published quantitative and qualitative studies on ulcerative colitis (UC) patient preference studies and existing clinical evidence on the safety and efficacy of filgotinib in treating UC. Search results were used to inform the outline and content of the qualitative interview guide. Two search strategies were executed using the Ovid database; articles identified from the search strategies were screened based on predefined eligibility criteria.

Overall, 17 full-text studies were included in the targeted literature review, consisting of 11 quantitative preference studies and 6 qualitative studies, as well a clinical trial protocol and one mixed-methods study report. Studies were conducted in Canada, Denmark, France, Germany, Netherlands, Poland, Spain and USA.

Treatment benefits and risks were identified (**Supplementary Tables 1 and 2**) as potential topics for further discussion in the qualitative interviews.

Treatment benefits (**Supplementary Table 1**) included:

- treatment administration
- likelihood of achieving remission
- time to achieve remission
- symptom improvement
- onset of action
- need for steroids
- monotherapy.

Treatment risks (**Supplementary Table 2**) included:

- risk of infections
- risk of gastrointestinal events
- risk of blood clots and major cardiovascular events
- risk of malignancies.

**Supplementary Text** **2.** Qualitative interviews.

*Approach*

The 60-minute virtual interview consisted of three parts.

- **Part 1:** patients were asked open-ended questions about their health-related quality of life symptoms and impacts, their current treatment experiences, and treatment expectations. The main objective of this part was concept elicitation.
- **Part 2:** patients discussed their attitudes toward and beliefs about treatments. This section was specifically concerned with the treatment and disease aspects identified from the evidence review.
- **Part 3:** a hypothetical choice task was discussed to assess how patients choose between treatment alternatives and trade-offs between treatment attributes.

*Findings*

Overall, 25 patients, spanning France (n = 5), Germany (n = 5), Italy (n = 5), Spain (n = 5) or the UK (n = 5) participated in the qualitative interviews. Patients had a mean age of 30.4 years (standard deviation, 8.9) and were predominantly female (n = 19, 76%). Fourteen patients were diagnosed with UC at least 5 years ago (56%), and four (16%) were newly diagnosed within 2–5 years before the interview. Eight patients (32%) reported that they were currently in remission, and most patients (n = 16, 64%) indicated they were currently experiencing symptoms. Many patients were taking more than one medication; mesalamine/mesalazine (n = 14; 56%) and rectal suppository or foam/cream steroids (n = 7; 28%) were the most reported. Eleven patients (44%) were receiving a biologic, and one patient (4%) was taking a Janus kinase inhibitor.

Thematic analysis findings from the qualitative interviews are summarized in **Supplementary Figure 2**, which is a conceptual map that provides a non-exhaustive summary of the key drivers of treatment valuation.

The analysis identified the following interrelated patient-relevant themes with multiple sub-themes.

- **Symptoms and impacts:** fifteen patients (60%) reported a stool frequency in the screening week that was higher than what they experienced in remission or before their UC diagnosis. Six patients (24%) experienced rectal bleeding less than half the time, and one patient (4%) experienced rectal bleeding half the time or more. The symptoms that led most patients to receive a UC diagnosis were rectal bleeding and abdominal pain. Patients reported being especially alarmed by bleeding, while the combined impact of bowel frequency, urgency, and potential incontinence left many patients feeling unable to leave their home, causing feelings of isolation. Many patients experienced anxiety and embarrassment when outside the home (including at work and social events) due to the unpredictability of their symptoms and the possibility of needing to quickly find a toilet. Embarrassment also affected relationships with friends and family. While pain had an effect on many patients, knowing its cause helped reduce the associated anxiety. Fatigue was reported as affecting physical activities and contributing to an emotional burden.
- **Treatment experience and expectations**: patients expected treatments to be efficacious, with an emphasis on achieving and maintaining remission. While remission was the most important treatment objective for most patients (n = 22; 88%), seven patients (28%) explicitly stated that they had not previously discussed expectations with their treating physician about achieving remission. In addition to achieving remission, symptom control was important to patients, with stool frequency (n = 13; 52%) and abdominal pain (n = 12; 48%) being the most important, followed by rectal bleeding (n = 11; 44%), urgency (n = 6; 24%), and bloating (n = 1; 4%). Most patients reported that their treatment helped reduce stool frequency (n = 20; 80%), bleeding (n = 19; 76%), and pain (n = 18; 72%). Of those patients who reported incontinence (n = 18; 72%), 16 patients (80%) found that their treatment helped. In contrast, 11 patients (65%) reported that their treatment did not improve fatigue.
- **Likelihood of achieving remission:** a series of scenarios was used to elicit patients’ minimum acceptable probability of remission, also referred to as minimum acceptable benefit (MAB). Patients were presented with an initial, hypothetical scenario of a new treatment, with a 10% chance of remission. Patients who indicated that they would not be willing to try this treatment were asked a series of follow-up questions, in which the probability of remission increased in 10% increments (with a maximum of 50%), to the point that the probability of remission was acceptable. The lowest probability of remission that a patient would accept was interpreted as the MAB. Twelve patients (48%) had a MAB of 10%, two patients (8%) had a MAB of 10–20%, two patients (8%) had a MAB of 20–30%, four patients (16%) had a MAB of 30–40%, and three patients (12%) had a MAB of more than 40%. One patient did not respond, and one patient was reluctant to try treatments that have recently arrived on the market. Seven patients (28%) would change their answer if the treatment also required steroid use, one patient did not change the stated MAB, and nine patients (36%) were unsure about how this would affect their MAB.
- **Time to achieve remission**: a similar format was used to elicit the maximum time that patients would accept from starting treatment to remission onset for a hypothetical treatment with a remission rate of 37%. Ten patients (40%) would accept a treatment that would take up to 10 weeks to achieve remission, five patients (20%) would accept a treatment that would take 10–20 weeks to achieve remission, and six patients (24%) would accept a treatment that would take 20–50 weeks to achieve remission. One patient did not respond. Three patients (12%) thought that 10 weeks was too long to wait to achieve remission, and one patient (4%) thought that a 37% success rate was too low to accept for any given time to remission.
- **Administration:** patients were experienced with a wide range of administration modes, including subcutaneous or intramuscular injections (n = 9; 36%); infusions (n = 15; 60%); oral tablets or granules (n = 21; 84%); and rectal suppositories, enemas, and foams (n = 11; 44%). Many patients (n = 17; 68%) used a combination of administration modes in their current therapy. When asked about their administration preference, if all other aspects were equal, 13 patients (52%) preferred an oral daily pill over a weekly injection, whereas eight patients (32%) preferred the opposite. However, when comparing a daily oral treatment to a monthly injectable, seven (54%) of the 13 patients (52%) who preferred an oral daily pill over a weekly injectable preferred the monthly injectable. Furthermore, three more patients (23%) switched their preference from oral to injection when asked to choose between a twice daily pill and a monthly injection; whereas nine patients (36%) preferred a daily oral pill over a monthly injection, 12 patients (48%) preferred the opposite. These findings suggest that administration preferences depend on both mode and frequency.
- **Treatment risks:** of a presented list of 19 potential adverse events (AEs), the most concerning AE selected by most patients (n = 20; 80%) was cancer, followed by depression (n = 13; 52%), upper respiratory infections (n = 11; 44%), and anxiety (n = 9; 36%). Side effects associated with short- and long-term steroid use were also worrisome for some patients. Nevertheless, many patients who reported using steroids considered them an effective short-term solution. The possibility of developing anti-drug antibodies that would reduce a drugs’ effectiveness over time was very concerning for some patients. Ten patients (40%) indicated that this would influence their treatment choice, whereas eight patients (32%) said it would not, and five patients (20%) were unsure. One patient (4%) had experience with developing antibodies; others were unsure of the meaning or the implications for treatment (22%). To help inform the selection of attribute levels included in the discrete choice experiment (DCE), maximum acceptable risk (MAR) levels for selected AEs were elicited in short thresholding exercises, holding other treatment aspects constant. Similar to the MAB elicitation, questions were asked sequentially from lowest risk level to highest, up to the point at which the patient would no longer accept the treatment.
- **Venous thromboembolism (VTE; framed as blood clots):** eight patients (32%) would accept a treatment with a ≥ 5% risk of VTEs. This means that their MAR was 5% or higher. Two patients (8%) had a MAR of 1% and 5%, five patients (20%) had a MAR of 0.5–1%, seven patients (28%) had a MAR of 0.1–0.5%, and three patients (12%) had a MAR of < 0.1% – meaning they would not accept any treatment option we provided that had a risk of blood clots. Most patients (n = 17; 68%) had not heard of the risk of VTEs before the interview.
- **Malignancies:** four patients (16%) had a MAR of ≥ 5%. Three patients (12%) had a MAR of 1–5%, two patients (8%) had a MAR of 0.5–1%, eight patients (32%) had a MAR of 0.1–0.5%, and eight patients (32%) had a MAR of < 0.1% – meaning they would not accept any treatment option we provided that had a risk of malignancies. Many patients (n = 13; 52%) had heard of this risk before.
- **Nausea and vomiting:** sixteen patients (64%) had a MAR of ≥ 20%. Four patients (16%) had a MAR of 10–20%. Three participants (12%) had a MAR of 5–10%.
- **Infections:** fourteen patients (56%) had a MAR of ≥ 20%. Six patients (24%) had a MAR of 10–20%. One participant (4%) had a MAR of 5–10%.

**Supplementary Text** **3.** Statistical analysis.

*Descriptive statistics*

Descriptive statistics were conducted on the final data set to summarize the following data quality indicators.

- **Health literacy:** this was scored by averaging the sum of the scores across the three items. Each question was assigned 0 (highest problems with reading) to 4 points (no problems with reading) to the scaled responses. Lower scores reflected greater problems with reading; scores ≤ 2 indicated ‘inadequate’ health literacy and a score >2 indicated ‘adequate’ health literacy.
- **Numeracy:** patients were given one point for each correctly answered question. The maximum score attainable on the numeracy scale was 5. Lower scores reflected greater problems with numbers; scores < 3 indicated ‘inadequate’ health numeracy and scores ≥ 3 indicated ‘adequate’ health numeracy.
- **Stability test**: a patient failed this test when they chose a different treatment option (alternative) in the repeated task.
- **Dominance test**: a patient failed this test when they did not choose the superior (dominated) option as a preferred treatment.
- **Trade-off behaviour**: patients’ choices generated information about the underlying attribute preferences when they made trade-offs among the attributes (e.g. willing to accept a higher level of pain to improve the risk of non-serious infections). Patients typically did not trade off when their choices were driven by only one attribute (e.g. always choosing the option with the lower risk of serious infections). This type of dominated decision making was analysed at the patient level by counting the number of times they selected the option offering the better level of each attribute. The decision making was dominated when choices in all 12 experimental tasks were driven by only one attribute. This analysis was possible for all attributes but the mode of administration (‘how and how often the treatment is taken’).
- **Serial non-participation**: experimental choice tasks data provided information about patients’ treatment attribute preferences and trade-offs among the attributes. A typical case of non-participation was identified as a choice pattern in which the patient always made choices based on the position of the options within the tasks (e.g. always choosing the A/left option). For each patient, non-participation was assessed by counting the number of times they selected the same option. A patient was classified as a serial non-respondent if they chose the same option in all 12 experimental choice tasks.
- **Response time**: the time patients took to complete all 12 experimental tasks was recorded.

*Analyses of discrete choice experiment data*

The DCE data were analysed within the random utility maximization (RUM) framework, which assumes that the respondent $n$ chooses the alternative $j$ in the DCE task $t$ that results in the highest utility.^1-4^ The treatment utility was defined as:

| $u(\boldsymbol{x}_{jnt})=v(\boldsymbol{x}_{jnt})+\varepsilon_{jnt}$ | (1) |
| --- | --- |

in which $\varepsilon_{jnt}$ is an identically and independently distributed extreme value type I error, and $v(\boldsymbol{x}_{jnt})$is the systematic utility component. This framework was used to estimate different discrete choice models, as described in the sections below. The systematic utility was defined as an additive function consisting of marginal utilities ($\beta_{k\in[1,13]}$) and an alternative-specific constant ($\alpha_{left})$.

| $v\left( x_{jnt} \right)=\alpha_{right}+\beta_{1}{admin:si}_{jnt}+\beta_{2}{admin:oral}_{jnt}+\beta_{3}{remission:40}_{jnt}+\beta_{4}{remission:60}_{jnt}$ $+\beta_{5}{steroid:1or2}_{jnt}+\beta_{6}{steroid:0}_{jnt}+\beta_{7}{bloodclots:0.5}_{jnt}+\beta_{8}{bloodclots:0.0}_{jnt}$ $+\beta_{9}{infections:1.5}_{jnt}+\beta_{10}{infections:0.0}_{jnt}+\beta_{10}{cancer:0.4}_{jnt}+\beta_{10}{cancer:0.0}_{jnt}$ | (2) |
| --- | --- |

The $\alpha_{right}$ parameter captures a systematic tendency to choose the right alternative, while the remaining parameters capture the effect of deviating from the reference level of an attribute on utility. The marginal utilities were assumed to follow a joined multivariate normal distribution (β~MVN[β ̅,Σ]) to account for preference heterogeneity among patients. A mean $\bar{\beta}_{k}$ and standard deviation $\sigma_{k}$ were obtained for each marginal utility. **Supplementary Table 5** also lists the parameters for this reference model.

The model’s statistical performance was assessed based on the Bayesian information criterion, adjusted McFadden pseudo-R^2^, and/or log-likelihood ratio test. The hierarchical Bayesian models were estimated with Monte Carlo Markov chain procedure using 300 000 draws prior convergence and 30 000 posterior draws; and every tenth posterior draw was retained to simulate the posterior distribution (thus leaving 3000 effective draws).^5, 6^

**Supplementary Text** **4.** Full online DCE survey

| **Introduction** |
| --- |

Thank you for taking part in this survey. This study is conducted by the scientific research company Evidera, on behalf of a pharmaceutical company. The objective is to understand participants’ preferences for treatments for ulcerative colitis (UC).

There are three sections in this survey that will take approximately 30 minutes in total to complete:

**Section 1** will provide you with some background information about ulcerative colitis and available treatments.

**Section 2** will ask you to choose several times between two treatments for ulcerative colitis. Each treatment has slightly different characteristics. Afterwards you will be asked to select the most and least important aspect of UC to be improved by treatments. These are established approaches that helps us understand how important different treatment aspects are to you.

**Section 3** will ask you to provide some information about yourself and your ulcerative colitis, such as the symptoms you are experiencing, when you were diagnosed, your age, and your most recent education.

Your responses will be private, and your name will never appear alongside your survey responses. All data will be anonymized and only used for medical research purposes. Findings in this research may be used to support the development of future treatments for ulcerative colitis.

**WEB PAGE BREAK**

| **Section 1: Background Information** |
| --- |

**About Ulcerative colitis**

Ulcerative colitis (UC) is a chronic and relapsing inflammatory condition of the gastrointestinal (GI) tract, where inflammation occurs in the large intestine, colon, and/or rectum. Small ulcers can also develop on the colon’s lining.

Symptoms of UC include abdominal pain or discomfort, abnormal bowel patterns, rectal bleedings, incontinence, bloating or distention, increased bowel urgency, fever, and fatigue.

**WEB PAGE BREAK**

**About Treatments for Ulcerative colitis**

There is unfortunately no cure for UC. Treatments therefore aim to improve symptoms. For example, treatments aim to stop rectal bleeding and to help you achieve a normal stool frequency. Typical treatments for UC include:

Aminosalicylates and steroids, which reduce inflammation in the lining of the intestine

Immunomodulators and Janus kinase (JAK) inhibitors, which reduce the overly active immune response that leads to inflammation. JAK inhibitors block certain enzymes which will stop autoimmune processes.

Biologics, which target specific proteins involved in the inflammatory process

Combination therapies are often prescribed because taking the drugs together makes them work better. For example, some UC patients may take a combination of biologic treatments and immunomodulators. Since immunomodulators can take a while to work, some patients may also be given steroids to help speed up their effects.

**WEB PAGE BREAK**

| **Section 2: Your treatment preferences** |
| --- |

In this section, you will be asked to make *[ADD FINAL NUMBER OF DCE CHOICE TASKS]* choices between two alternative treatments for ulcerative colitis. Each of the treatments will be described by six characteristics, or attributes. These are listed below.

It is important that you consider all the six characteristics carefully. They will be introduced to you one by one before you will be asked to make the choices.

You can hover with your mouse over the characteristics in the first column for more information.


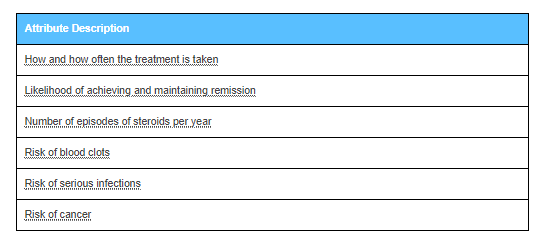


|  | **Treatment A** |  | **Treatment B** |
| --- | --- | --- | --- |
|  | \| 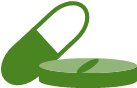 \| Oral pill  at home every day \| \| --- \| --- \| |  | \| 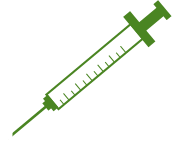 \| Self-injection at home every 1 to 2 weeks \| \| --- \| --- \| |

How the treatment is taken can differ between medicines. Some medicines can be taken at home, either every day with an oral pill, or an injection every 1-2 weeks. Other medicines need to be given to you at a hospital or clinic by an intravenous treatment, which means it is administered directly through your vein.

**From the list below, what is your most and what your least preferred way of taking your medicine?***Select one option per column*

|  |  | Most preferred | Least preferred |
| --- | --- | --- | --- |
| 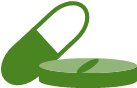 | Oral pill every day | □ | □ |
| 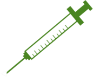 | Self-injection at home every 1 to 2 weeks | □ | □ |
| 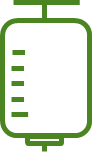 | Intravenous treatment in hospital  or clinic every 4 to 8 weeks | □ | □ |

**[PROGRAMMER NOTE: ONLY ONE SELECTION PER COLUMN ALLOWED, SELECTIONS MUST BE ON DIFFERENT ROWS]**

**WEB PAGE BREAK**


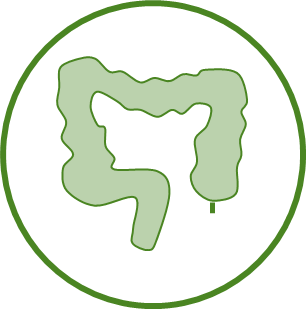


UC treatments may help you achieve and maintain remission. This means that you will have no rectal bleeding and a close to normal stool frequency.

The example picture below shows a 40% chance of achieving and maintaining remission

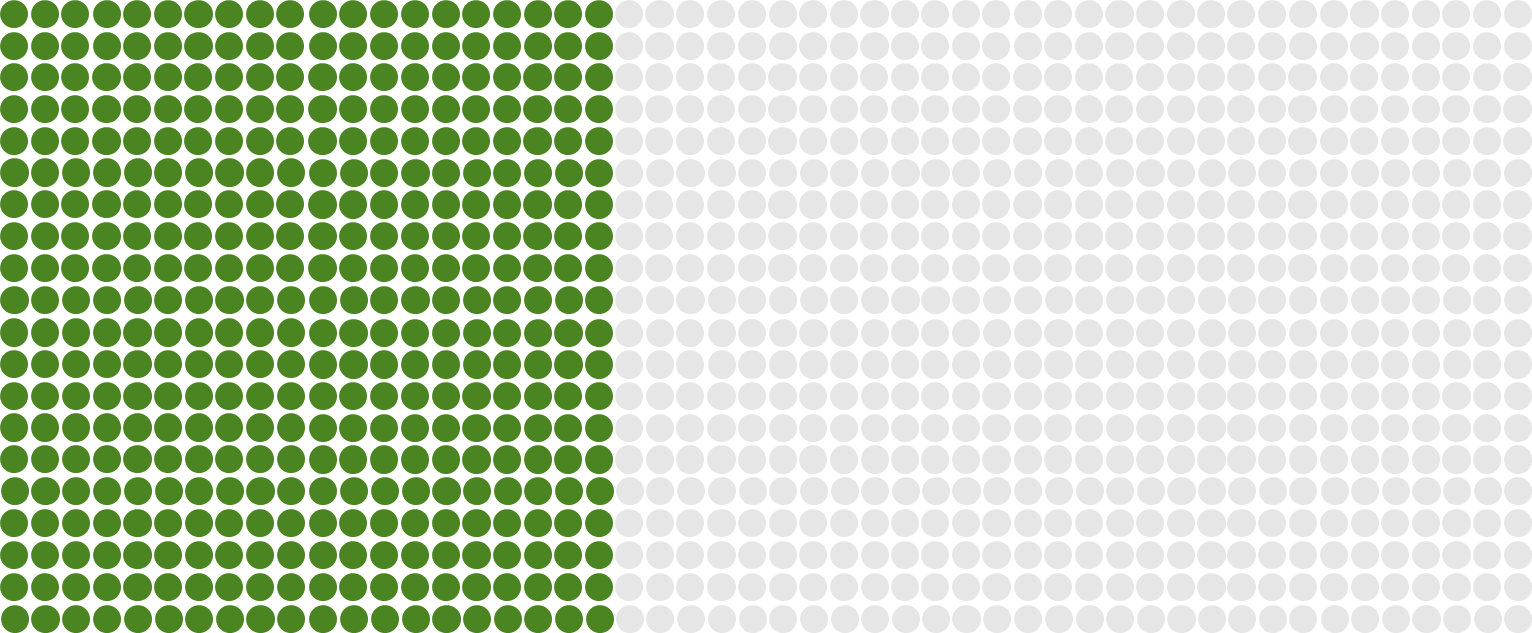


400 out of 1000 patients (40%)

The figures in **green** (400 out of 1000 or 40%) represent the number
of people who achieve and maintain remission.

The figures in **grey** (600 out of 1000 or 60%) represent the number
of people who do not achieve and maintain remission.

**Please compare the treatment options below. If all other aspects of the treatment are the same, which one is better?**

|  | **Treatment A** |  | **Treatment B** |
| --- | --- | --- | --- |
|  | 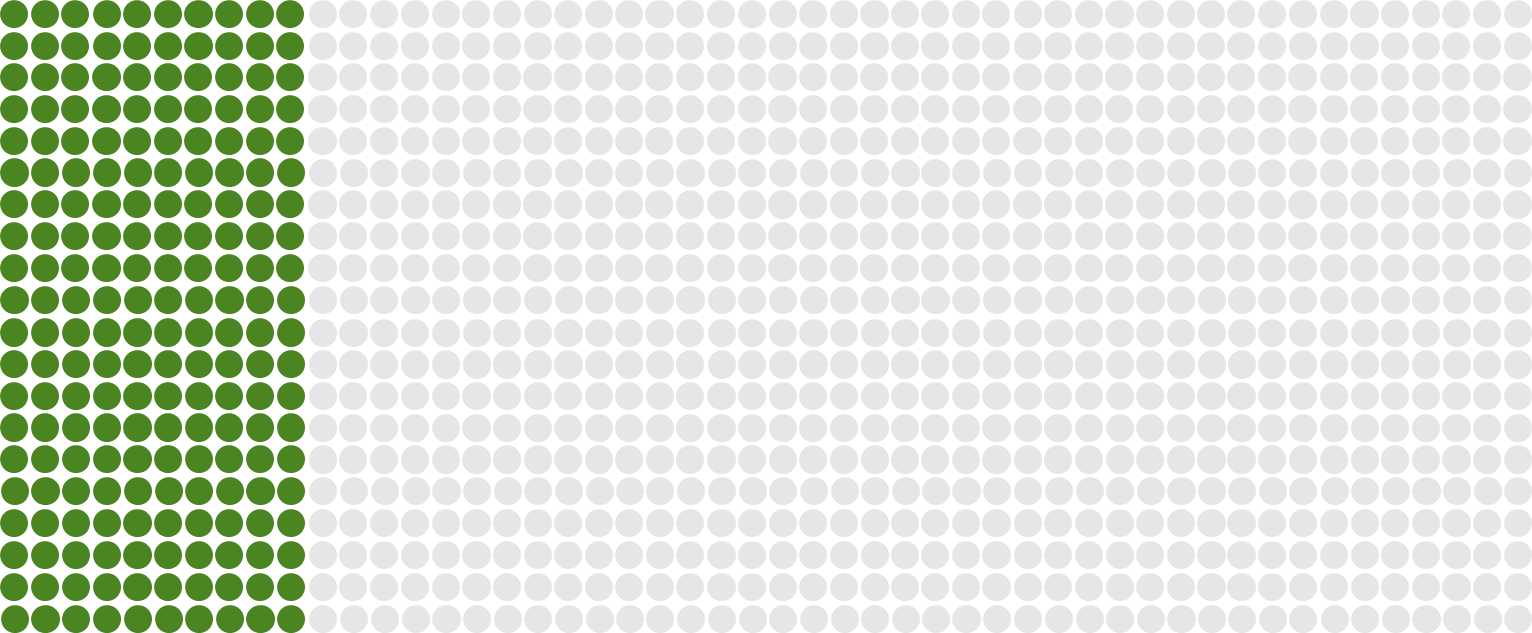  200 out of 1000 patients (20%) will  *achieve and maintain remission* |  | \| 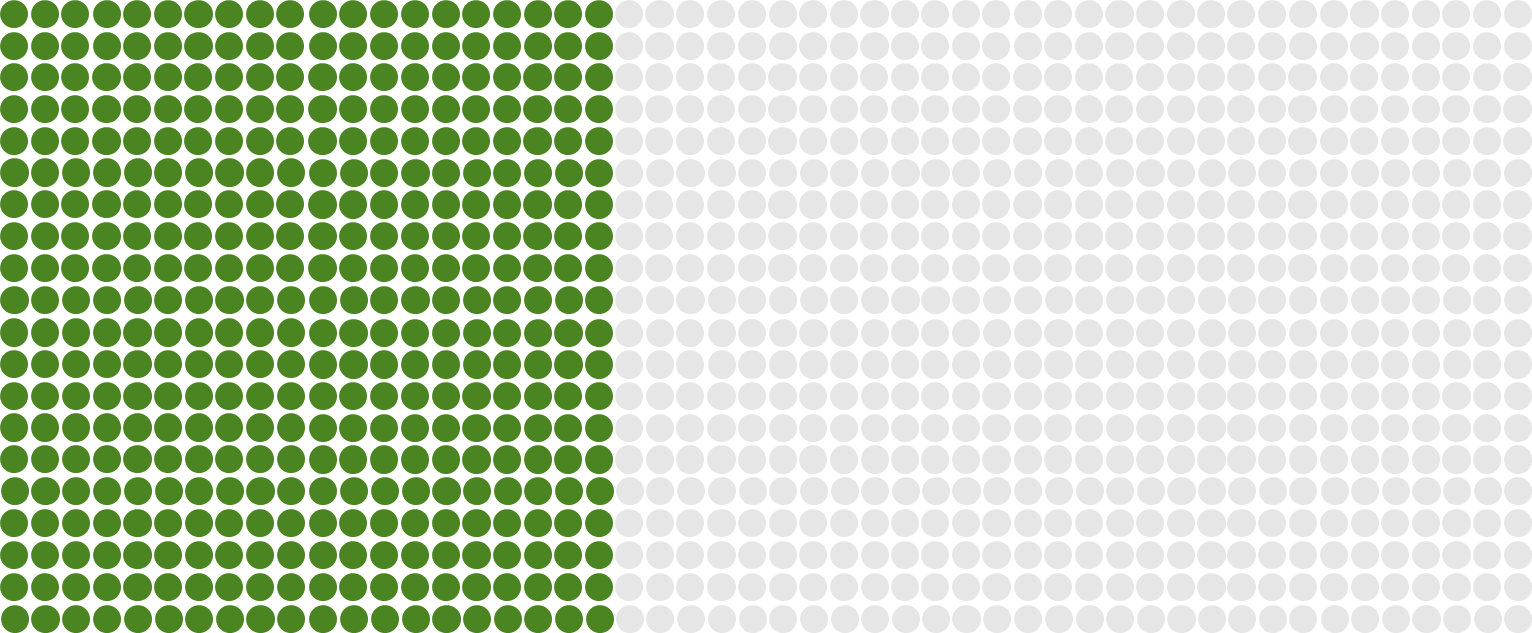 \| \| --- \| \| 400 out of 1000 patients (40%) will  *achieve and maintain remission* \| |
|  | □ |  | □ |

**[PROGRAMMER NOTE: ONLY ONE SELECTION ALLOWED]**

[If Treatment A is selected, display a pop-up with red text: “**NOT correct!** The chance of remission is higher with Treatment B than with Treatment A.”

If Treatment B is selected, display a pop-up with green text: “**Correct!** The chance of remission is lower with Treatment B than with Treatment A.”]

**WEB PAGE BREAK**

Steroids are often used to treat the symptoms of UC, in combination with other therapies.

One episode (course) of steroid use refers to the use of steroids for a period of three months or less. Two episodes (courses) refers to separate courses of steroid use of three months or less, separated by a period of no steroid use in between.

Each episode of steroid use can result in weight gain, mood swings, acne, problems sleeping, growth of facial hair, or concentration problems.

**How many episodes of steroids do you usually have per year?***Select one option*

| No episodes (courses) per year | □ |
| --- | --- |
| 1 to 2 episodes (courses) per year | □ |
| 3 to 4 episodes (courses) per year | □ |
| More than 4 episodes (courses) per year | □ |

**[PROGRAMMER NOTE: ONLY ONE SELECTION ALLOWED]**


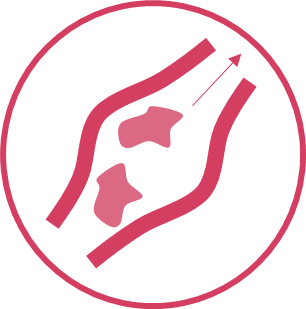


UC patients have an increased risk of blood clots that can also be affected by treatments. This is a clump of blood that forms in your veins and that can travel through your body. Blood clots are a medical emergency and are often treated with blood thinning medication and may require you to stay in the hospital. Blood clots can result in life-threatening complications (e.g. pulmonary embolism, heart attack, stroke).

The example picture below shows a 1% risk of developing blood clots,

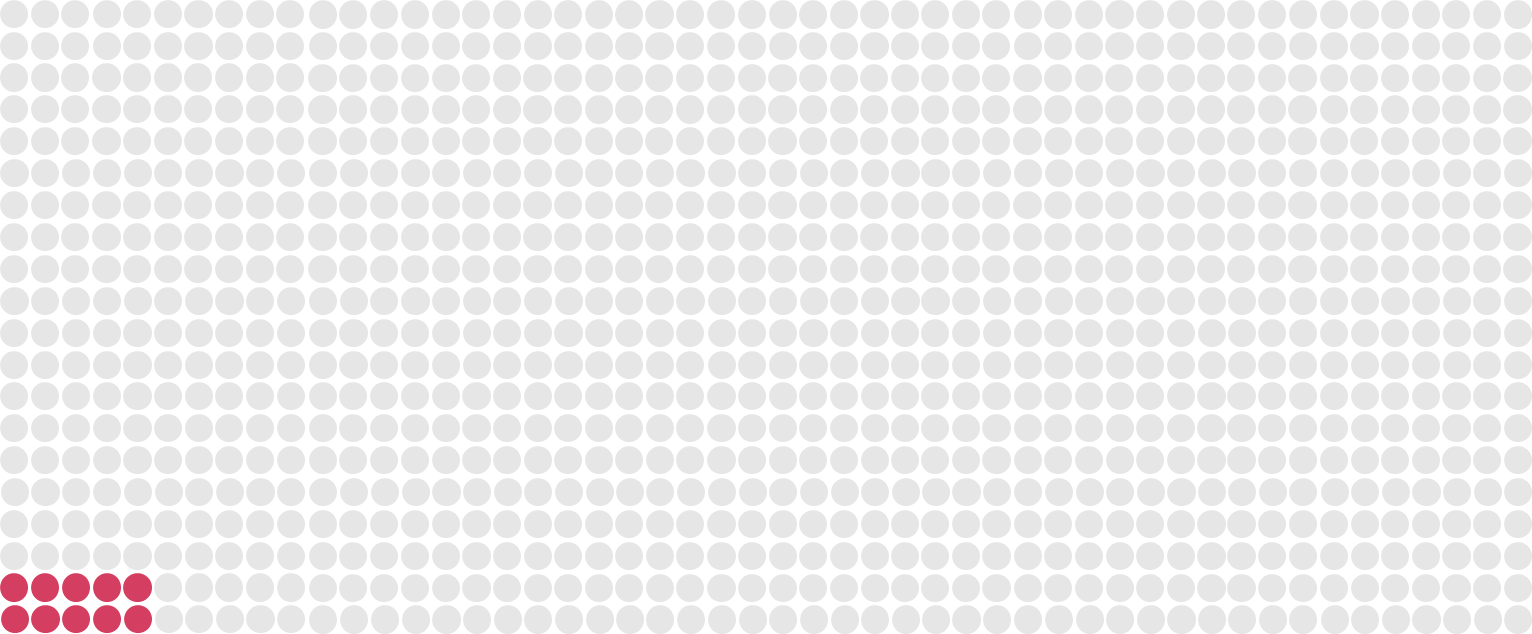


10 out of 1000 patients (1%)

The figures in **green** (10 out of 1000 or 1%) represent the number
of people who develop blood clots.

The figures in **grey** (990 out of 1000 or 99%) represent the number
of people who do not develop blood clots.

**Please compare the treatment options below. If all other aspects of the treatment are the same, which one is better?**

| **Treatment A** |  | **Treatment B** |
| --- | --- | --- |
| 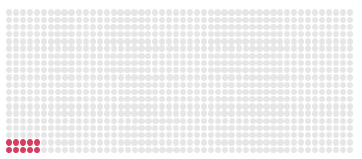 10 of 1,000 patients (1%)  will develop *blood clots* |  | 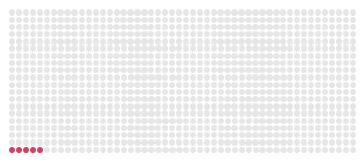 5 of 1,000 patients (0.5%)  will develop *blood clots* |
| □ |  | □ |

**[PROGRAMMER NOTE: ONLY ONE SELECTION ALLOWED]**

[If Treatment A is selected, display a pop-up with red text: “**NOT correct!** The risk of blood clots is lower with Treatment B than with Treatment A.”

If Treatment B is selected, display a pop-up with green text: “**Correct!** The risk of blood clots is lower with Treatment B than with Treatment A.”]

**WEB PAGE BREAK**


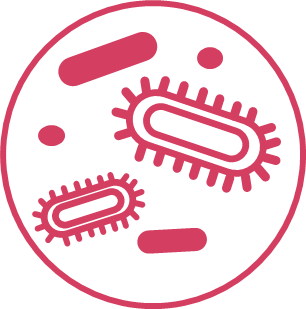


Some ulcerative colitis treatments increase the risk of patients developing serious infections, such as pneumonia or serious and painful forms of shingles. Serious infections require hospitalization and may become life threatening. Different treatments have different risks of causing serious infections.

**Please compare the treatment options below. If all other aspects of the treatment are the same, which one is better?**

| **Treatment A** |  | **Treatment B** |
| --- | --- | --- |
| 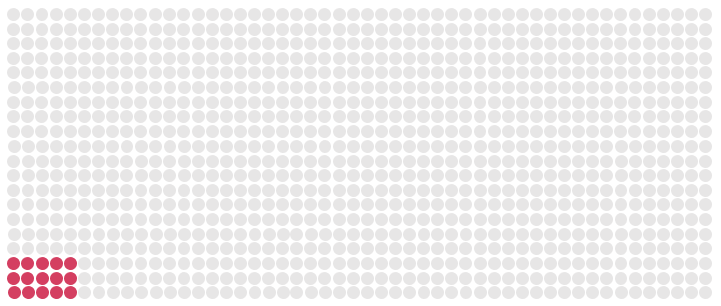 15 of 1,000 patients (1.5%)  will develop *serious infections* |  | 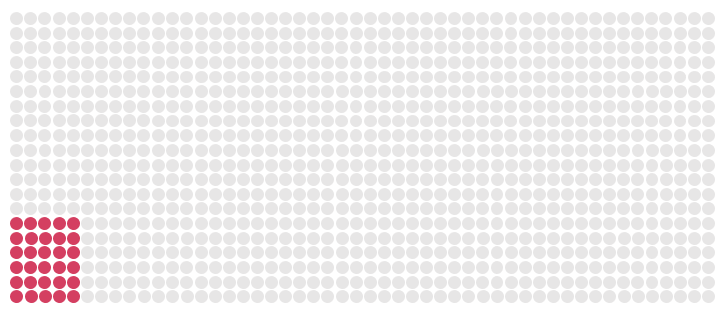 30 of 1,000 patients (3%)  will develop *serious infections* |
| □ |  | □ |

**[PROGRAMMER NOTE: ONLY ONE SELECTION ALLOWED]**

[If Treatment A is selected, display a pop-up with red text: “**Correct!** The risk of serious infections is lower with Treatment A than with Treatment B.”

If Treatment B is selected, display a pop-up with green text: “**NOT correct!** The risk of serious infections is lower with Treatment A than with Treatment B.”]

**WEB PAGE BREAK**


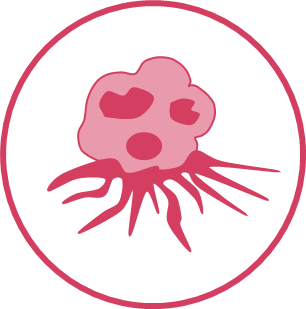


You have a risk of developing cancer even without taking treatments and this increases with age. Some UC treatments can increase your risk of certain types of cancer (lymphomas, and non-melanoma skin cancer). Cancer typically requires chemotherapy or surgery, and some cancers can be life-threatening. Some cancers can be treated or cured with treatment, while others may not be treatable. You may need to temporarily or permanently stop your UC treatment.

**Please compare the treatment options below. If all other aspects of the treatment are the same, which one is better?**

|  | **Treatment A** |  | **Treatment B** |
| --- | --- | --- | --- |
|  | 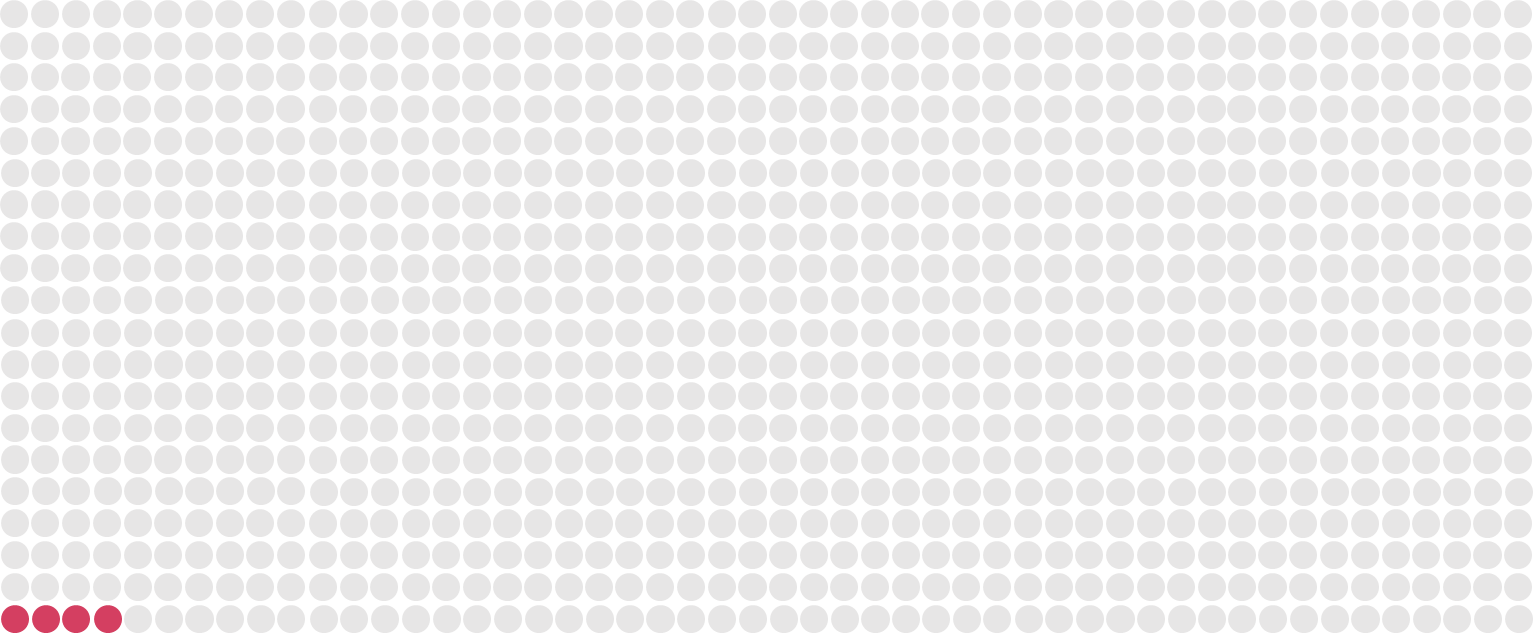 4 of 1,000 patients (0.4%)  will develop *cancer* |  | 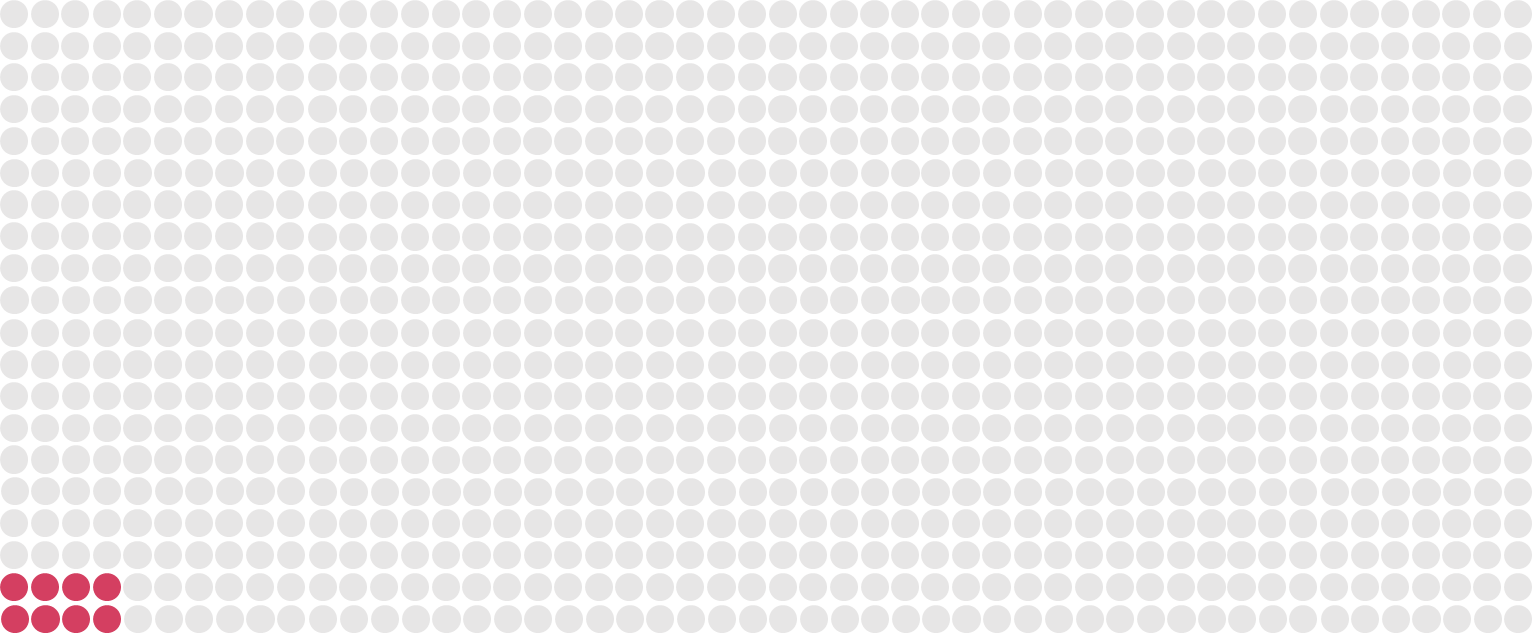 8 of 1,000 patients (0.8%)  will develop *cancer* |
|  | □ |  | □ |

**[PROGRAMMER NOTE: ONLY ONE SELECTION ALLOWED]**

[If Treatment A is selected, display a pop-up with red text: “**Correct!** The risk of cancer is lower with Treatment A than with Treatment B.”

If Treatment B is selected, display a pop-up with green text: “**NOT correct!** The risk of cancer is lower with Treatment A than with Treatment B.”]

**WEB PAGE BREAK**

| **Treatment A** |  | **Treatment B** |
| --- | --- | --- |
| \| 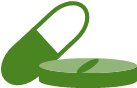 \| Oral pill  at home every day \| \| --- \| --- \| |  | \| 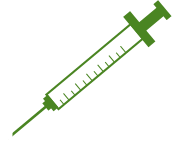 \| Self-injection at home every 1 to 2 weeks \| Self-injection  at home every 8 weeks \| \| --- \| --- \| --- \| |
| \| 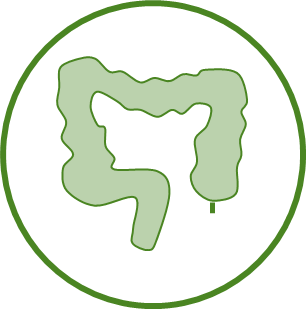 \| 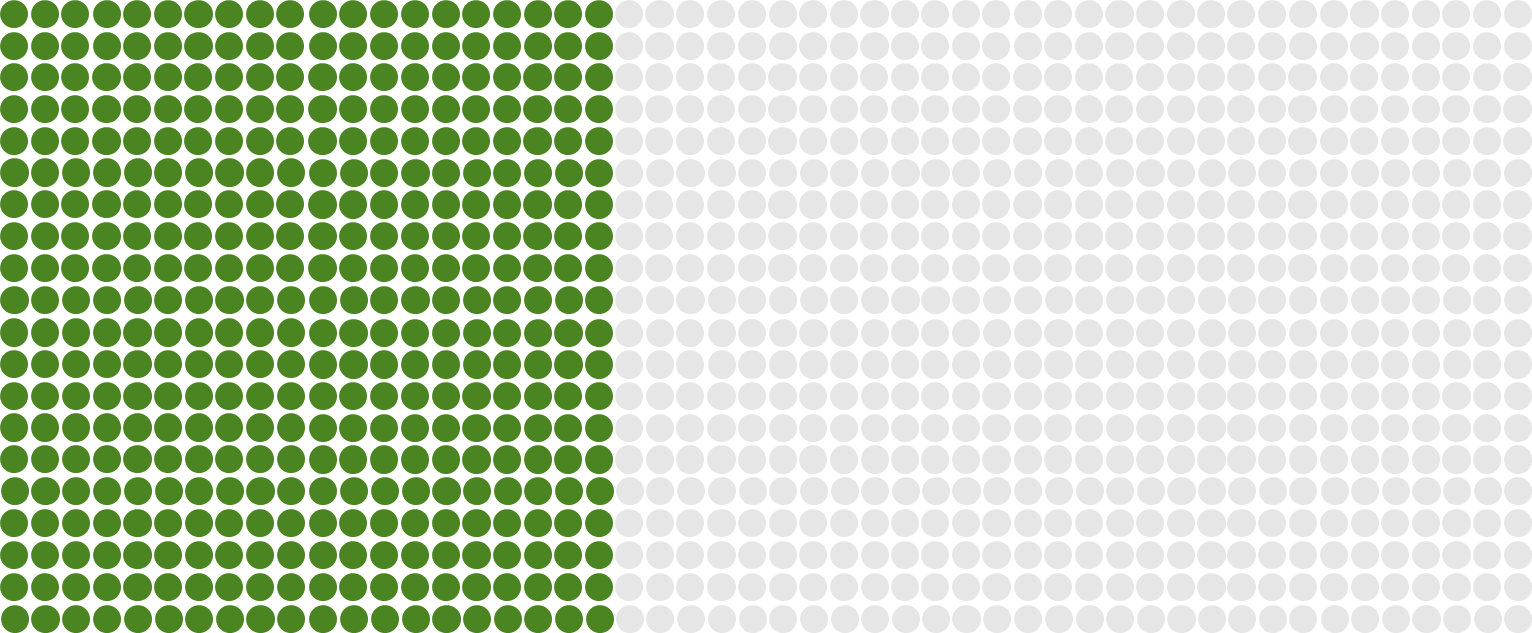 400 of 1,000 patients (40%) will  *achieve and maintain remission* \| \| --- \| --- \| |  | \| 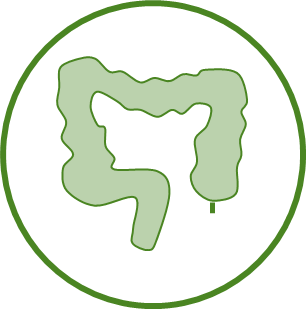 \| 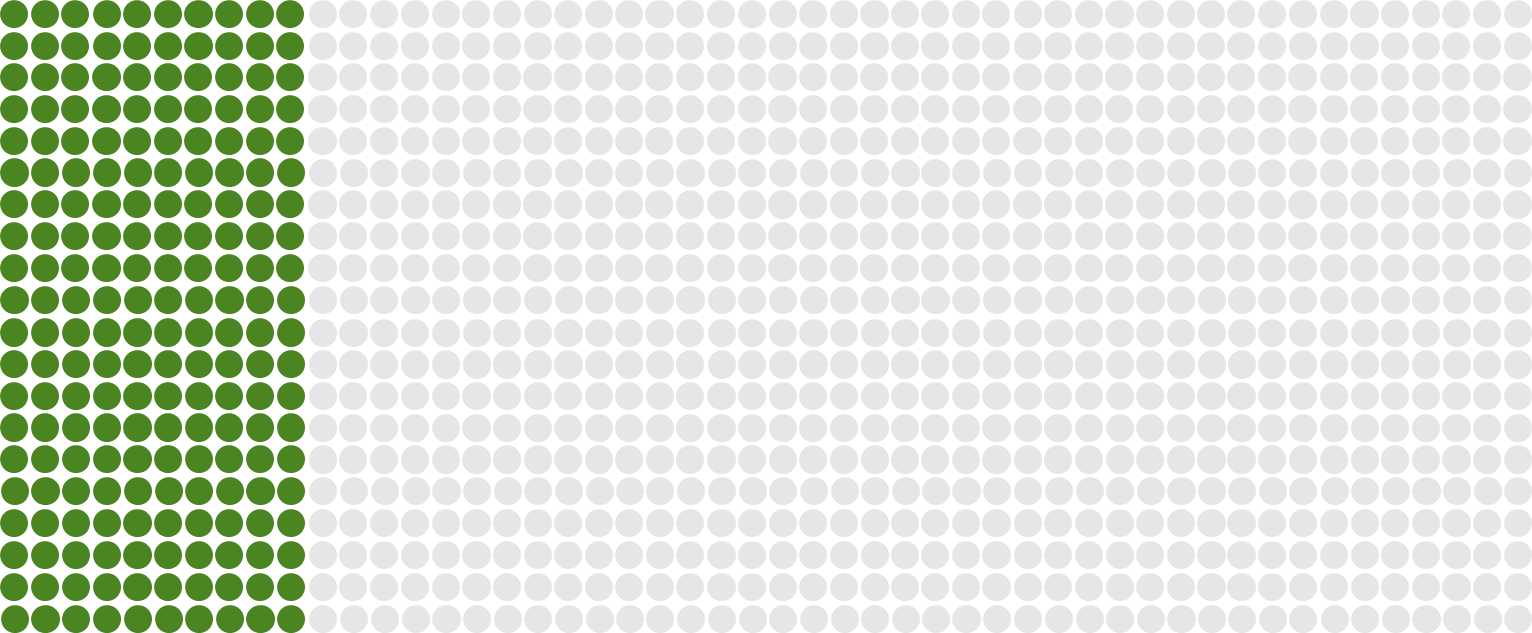 200 of 1,000 patients (20%) will  *achieve and maintain remission* \| \| --- \| --- \| |
| 0 episodes (courses) of *steroids* within a year | Now comes the section where you will be asked to choose between two treatments.  Let’s do a practice! | 1 to 2 episodes (courses) of *steroids* within a year |
| \| 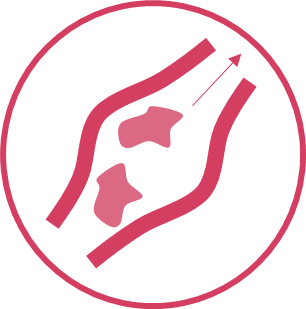 \| 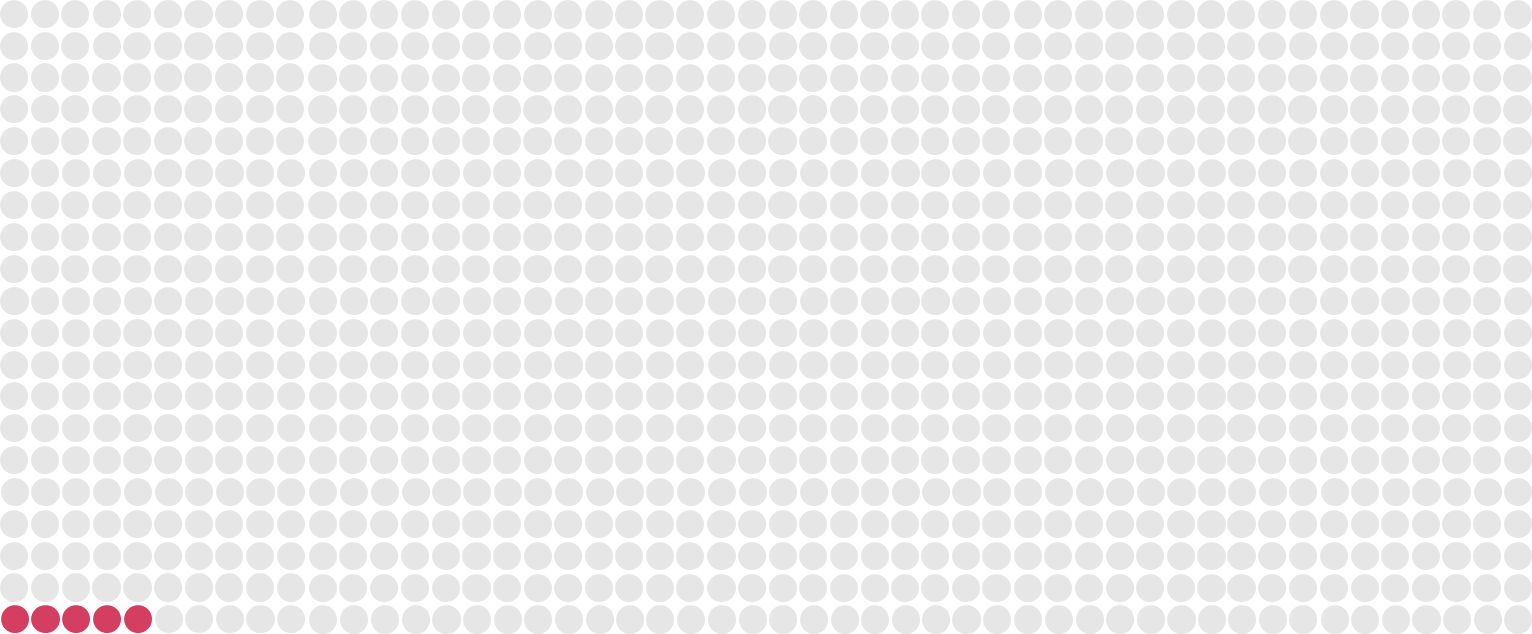 5 of 1,000 patients (0.5%) will develop *blood clots* \| \| --- \| --- \| |  | \| 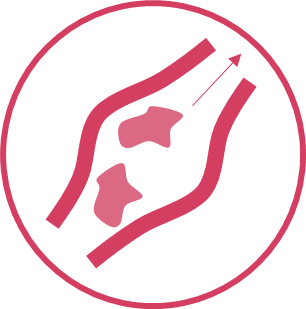 \| 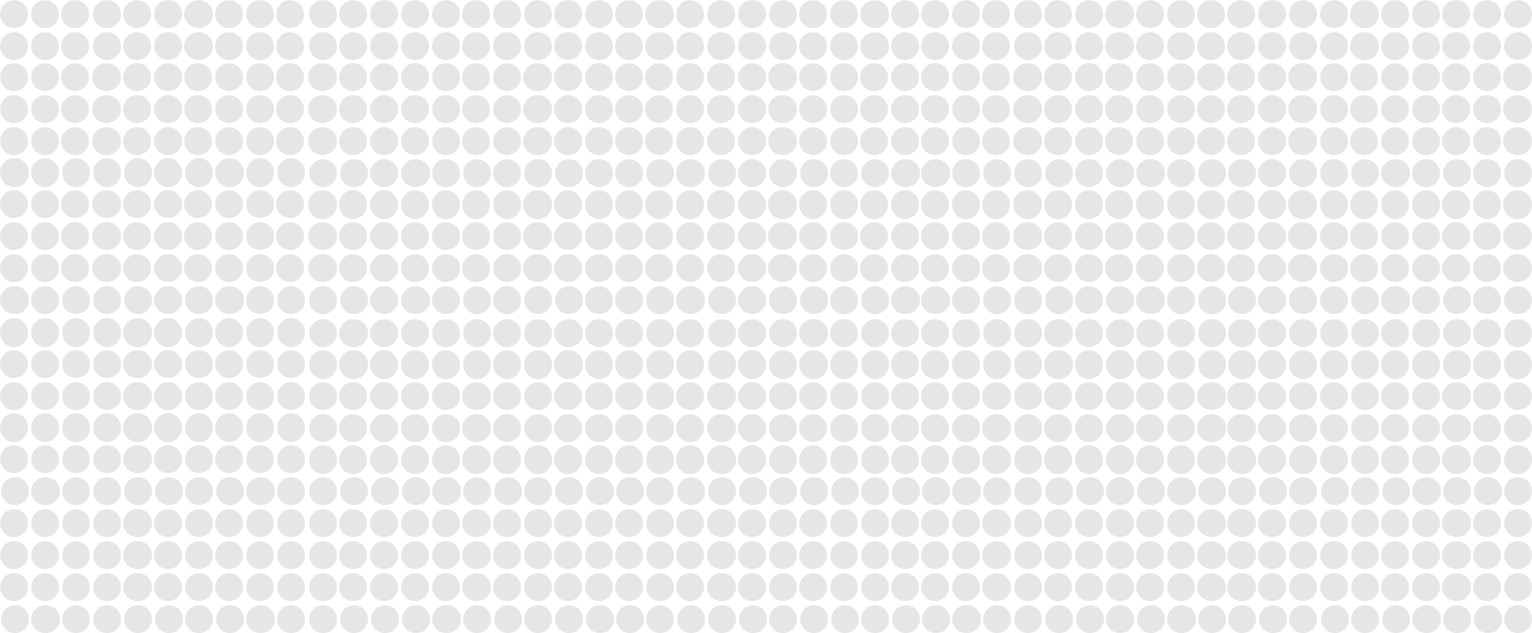 0 of 1,000 patients (0.0%) will develop *blood clots* \| \| --- \| --- \| |
| \| 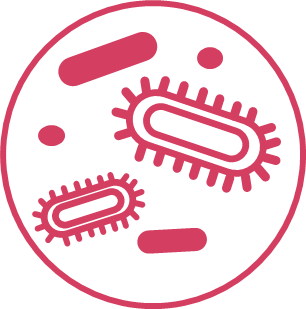 \| 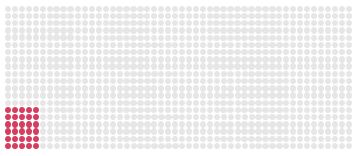 30 of 1,000 patients (3.0%)  will develop *serious infections* \| \| --- \| --- \| |  | \| 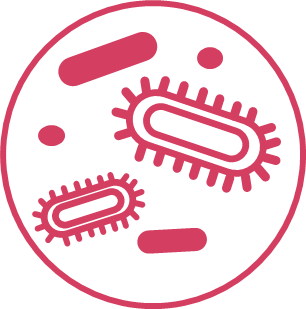 \| 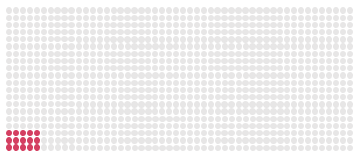 15 of 1,000 patients (4.0%)  will develop *serious infections* \| \| --- \| --- \| |
| \| 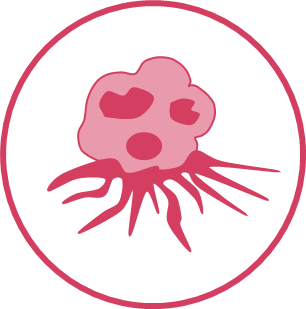 \| 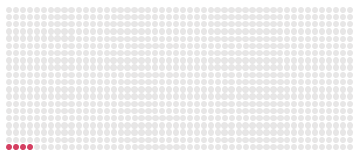 4 of 1,000 patients (0.4%)  will develop *cancer* \| \| --- \| --- \| |  | \| 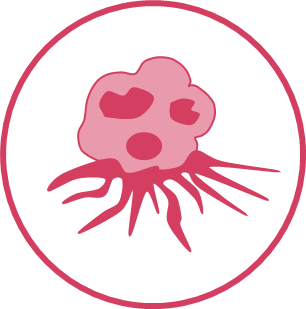 \| 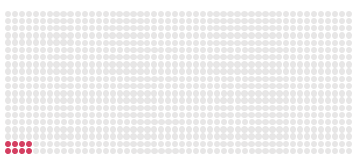 8 of 1,000 patients (0.8%)  will develop *cancer* \| \| --- \| --- \| |
| □ |  | □ |

| **Treatment A** |  | **Treatment B** |
| --- | --- | --- |
| \| 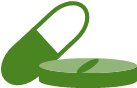 \| Oral pill  at home every day \| \| --- \| --- \| |  | \| 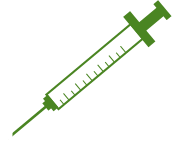 \| Self-injection at home every 1-2 weeks \| Self-injection  at home every 8 weeks \| \| --- \| --- \| --- \| |
| \| 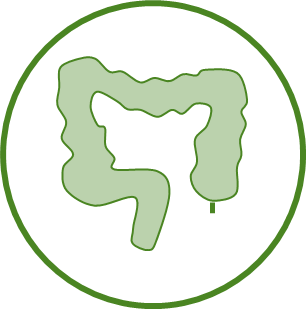 \| 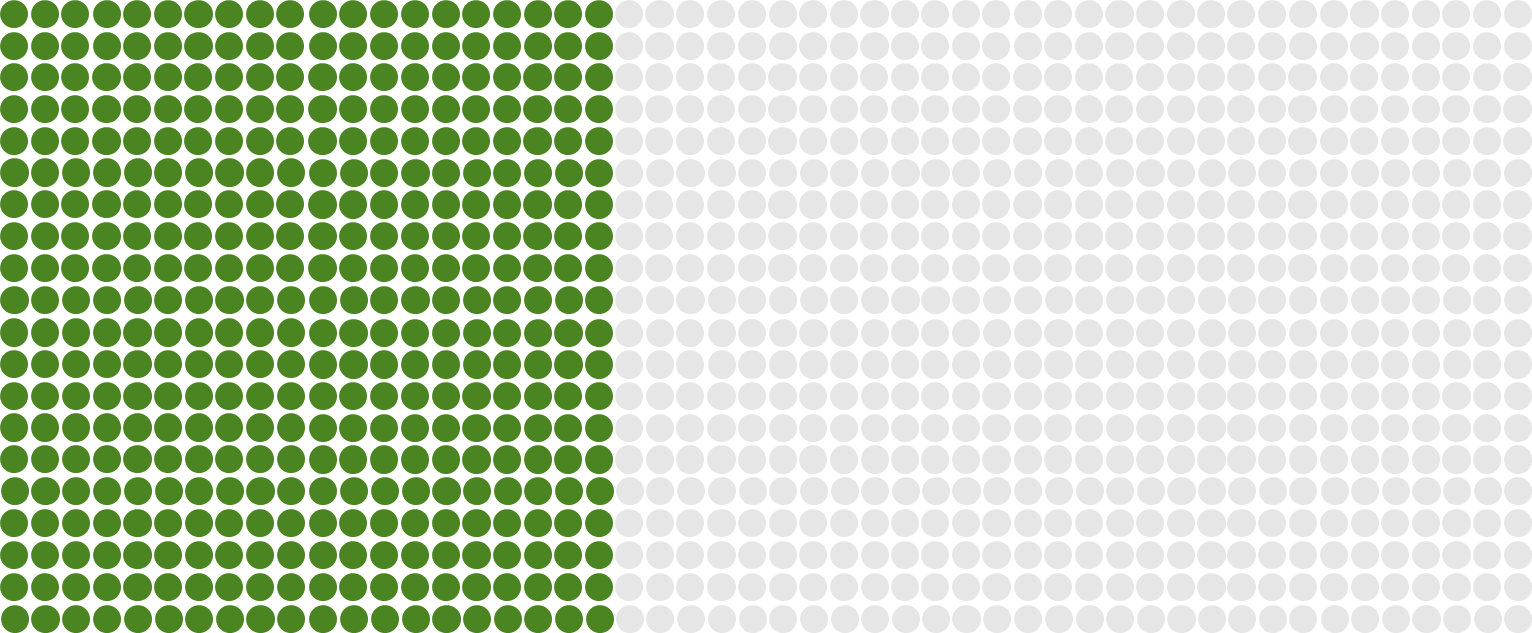 400 of 1,000 patients (40%) will  *achieve and maintain remission* \| \| --- \| --- \| |  | \| 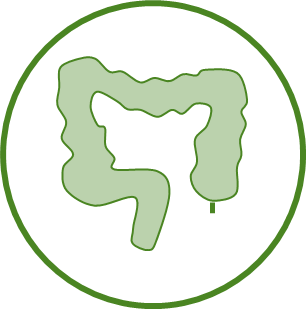 \| 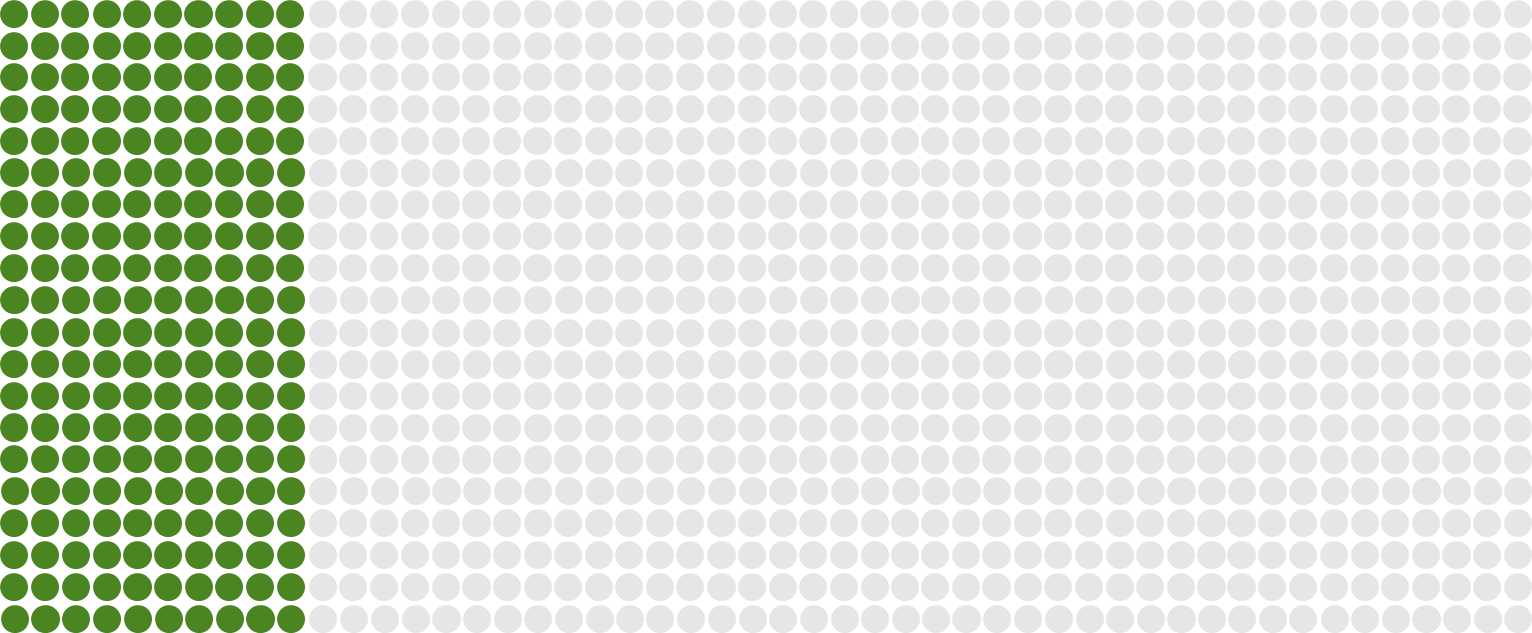 200 of 1,000 patients (20%) will  *achieve and maintain remission* \| \| --- \| --- \| |
| 0 episodes (courses) of *steroids* within a year |  | 1 to 2 episodes (courses) of *steroids* within a year |
| \| 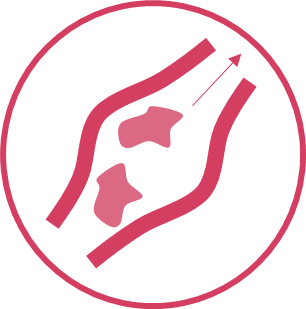 \| 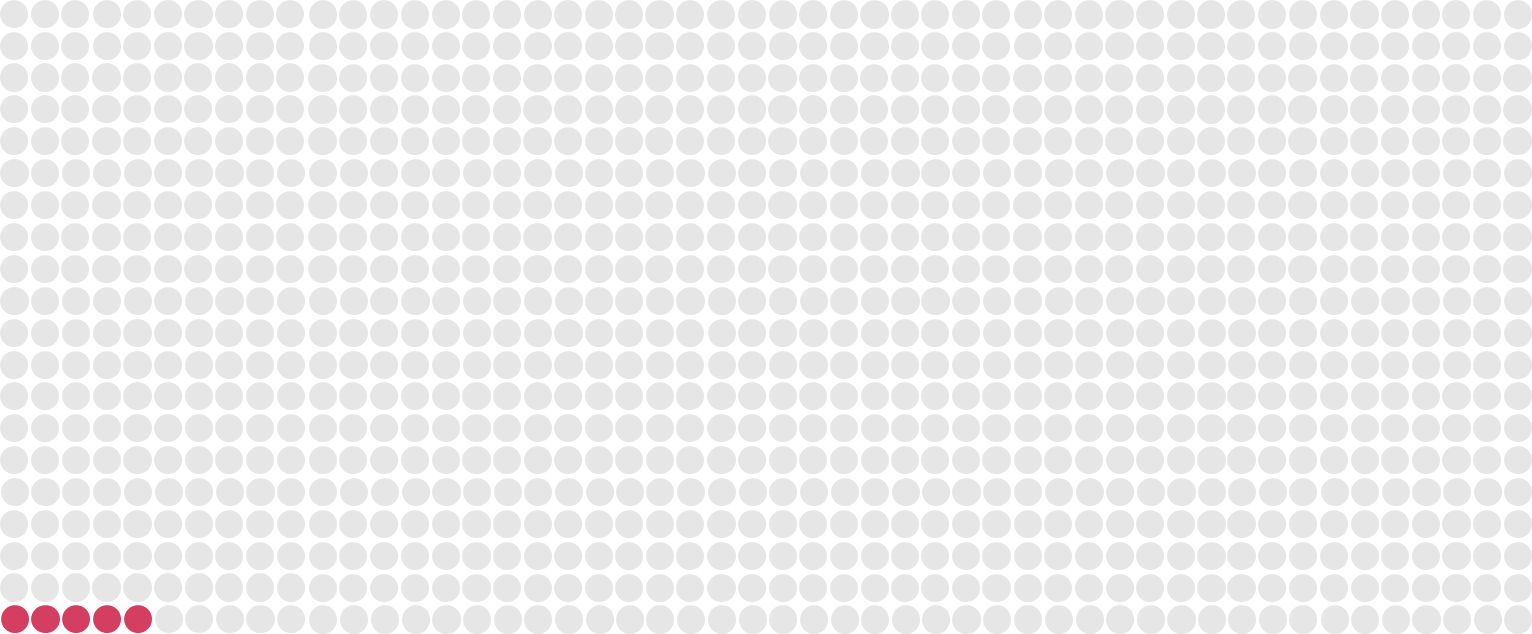 5 of 1,000 patients (0.5%) will develop *blood clots*  The two treatments differ in how they are taken and the likelihood of helping patients achieve and maintain remission after 10 weeks. They also differ in how many episodes (courses) of steroids are needed to manage symptoms.  While these treatment outcomes may differ from your personal experience, it is important to consider and weigh them all. \| \| --- \| --- \| |  | \| 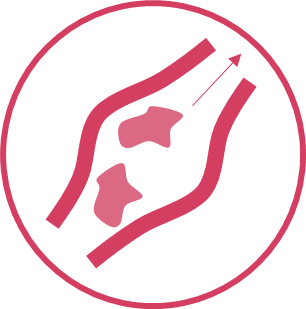 \| 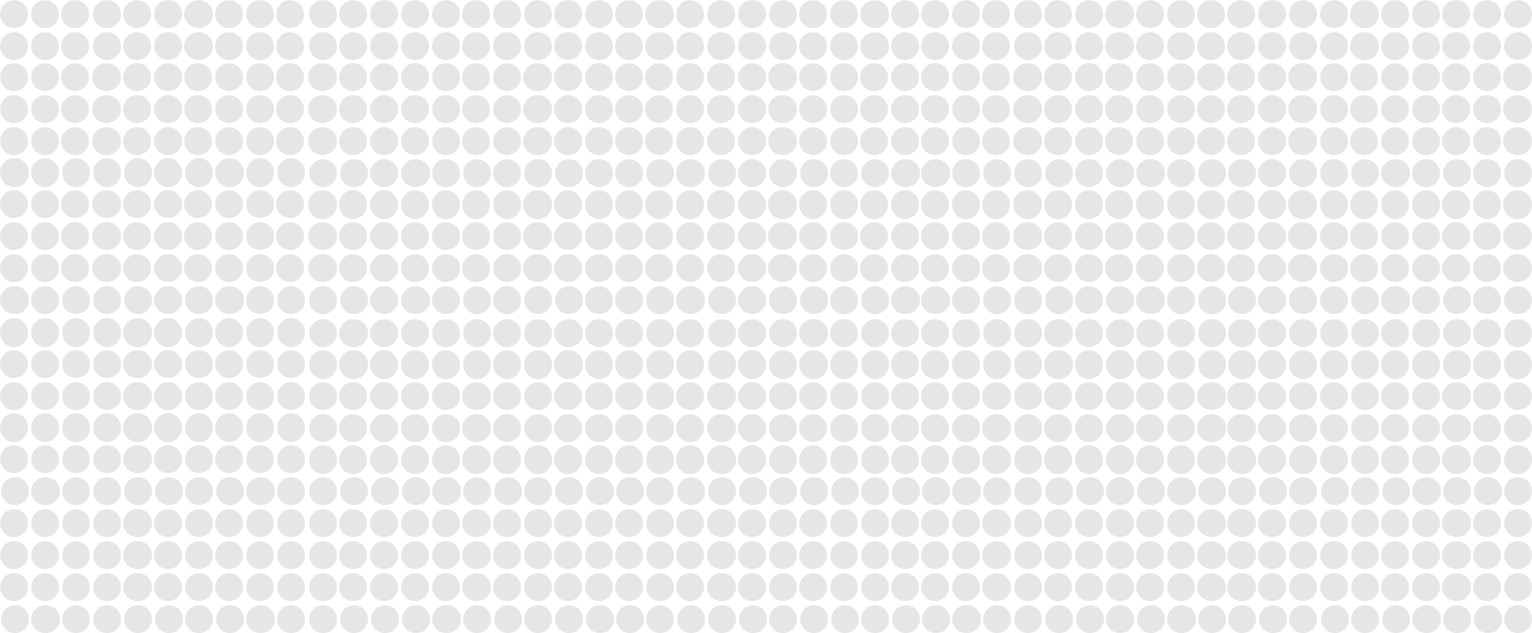 0 of 1,000 patients (0.0%) will develop *blood clots* \| \| --- \| --- \| |
| \| 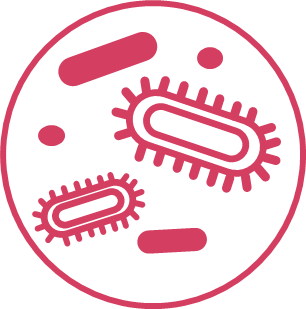 \| 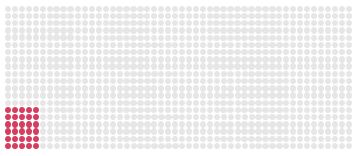 30 of 1,000 patients (3.0%)  will develop *serious infections* \| \| --- \| --- \| |  | \| 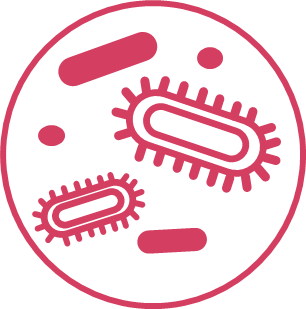 \| 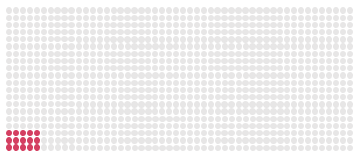 15 of 1,000 patients (4.0%)  will develop *serious infections* \| \| --- \| --- \| |
| \| 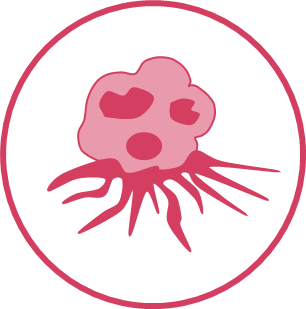 \| 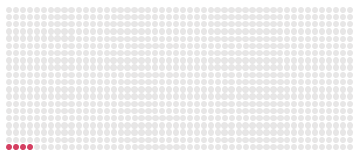 4 of 1,000 patients (0.4%)  will develop *cancer* \| \| --- \| --- \| |  | \| 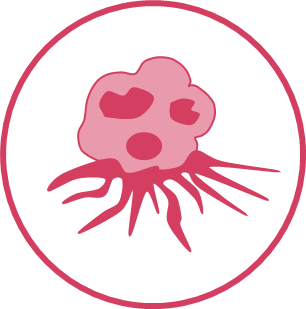 \| 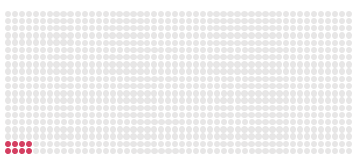 8 of 1,000 patients (0.8%)  will develop *cancer* \| \| --- \| --- \| |
| □ |  | □ |

| **Treatment A** |  | **Treatment B**  The two treatments are also associated with different risks of blood clots, serious infections, and cancer. Please weigh these risks carefully against the benefits of the treatment.  When you are ready, close this window and make your choice.  [Programmers: Make sure you put a NEXT button here to close this window] |
| --- | --- | --- |
| \| 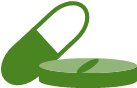 \| Oral pill  at home every day \| \| --- \| --- \| |  | \| 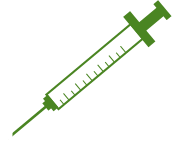 \| Self-injection at home every 1 to 2 weeks \| Self-injection  at home every 8 weeks \| \| --- \| --- \| --- \| |
| \| 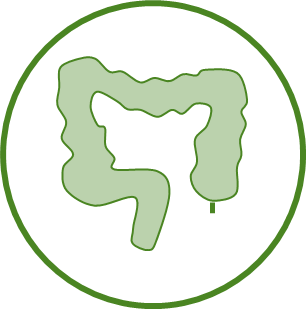 \| 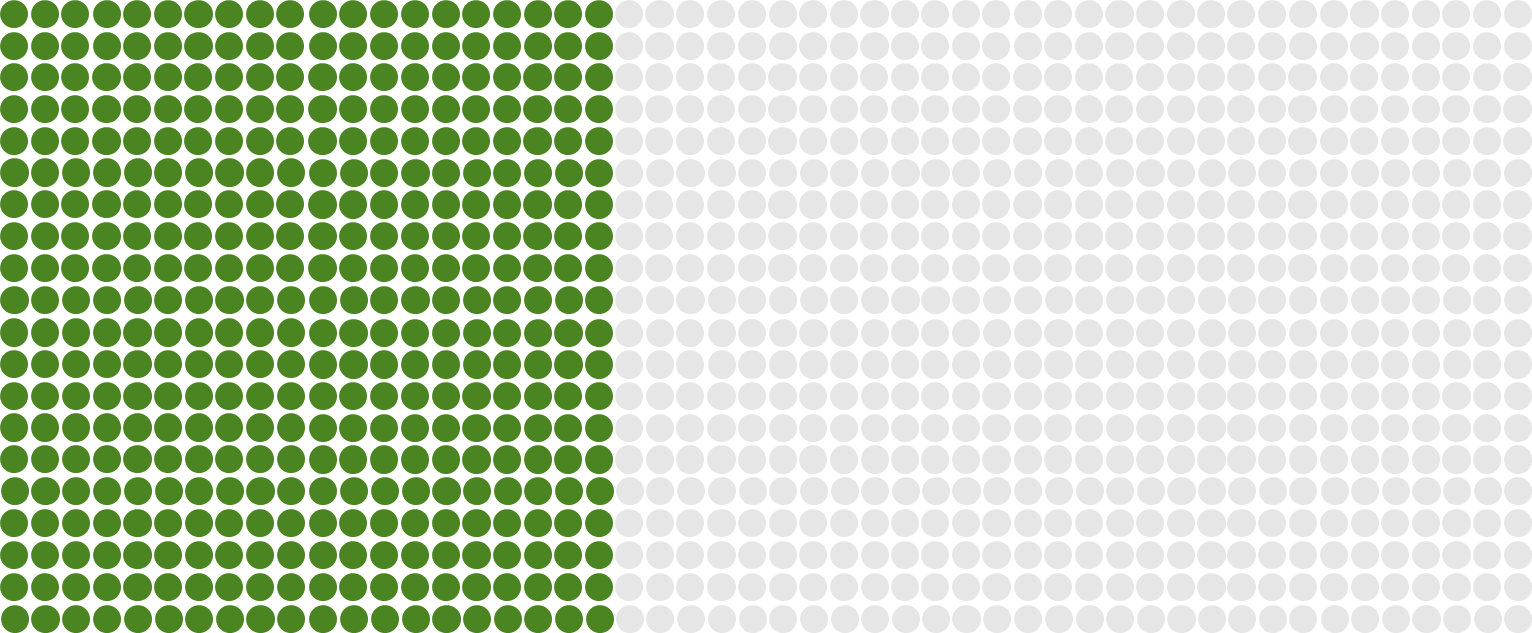 400 of 1,000 patients (40%) will  *achieve and maintain remission* \| \| --- \| --- \| |  | \| 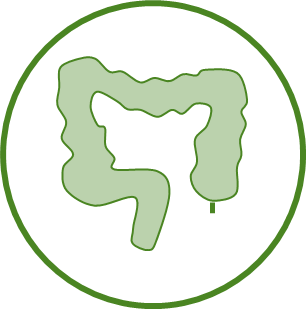 \| 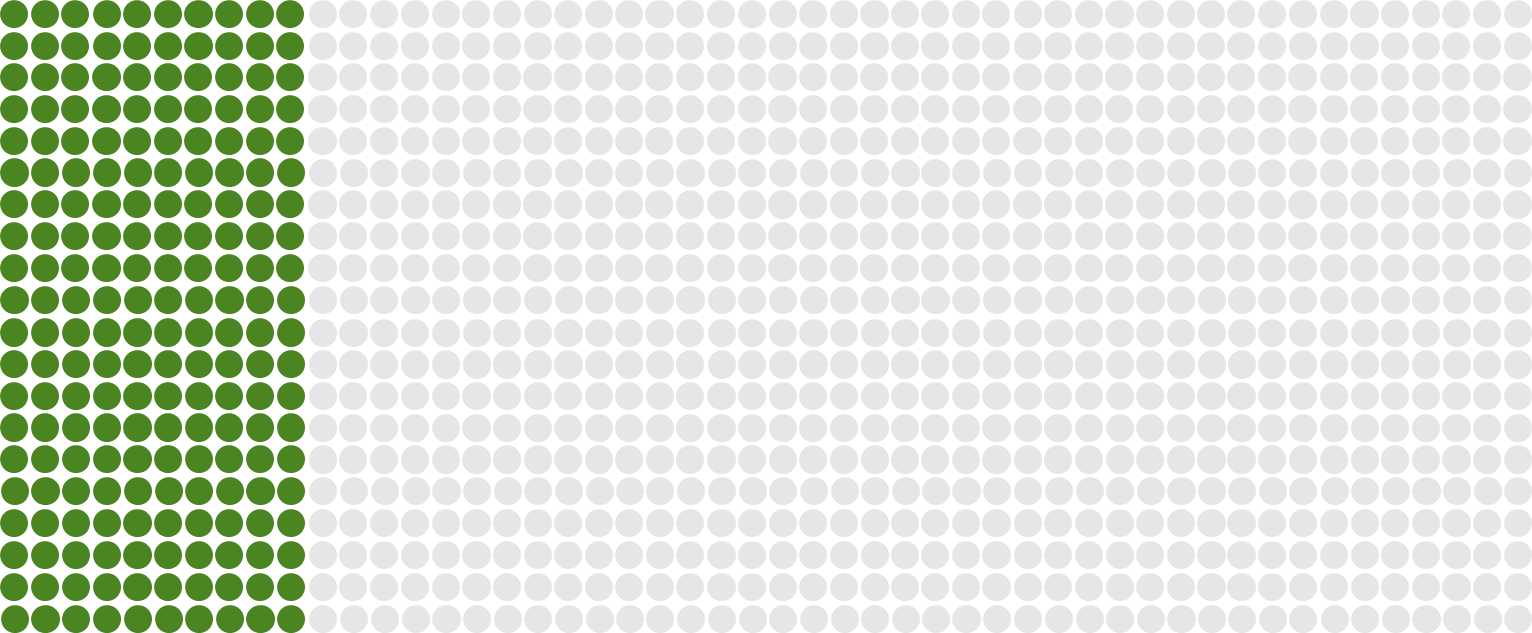 200 of 1,000 patients (20%) will  *achieve and maintain remission* \| \| --- \| --- \| |
| 0 episodes (courses) of *steroids* within a year |  | 1 or 2 episodes (courses) of *steroids* within a year |
| \| 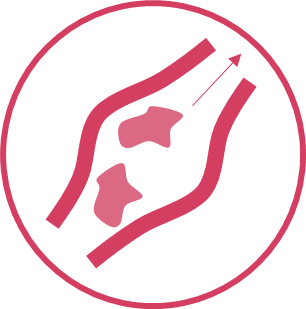 \| 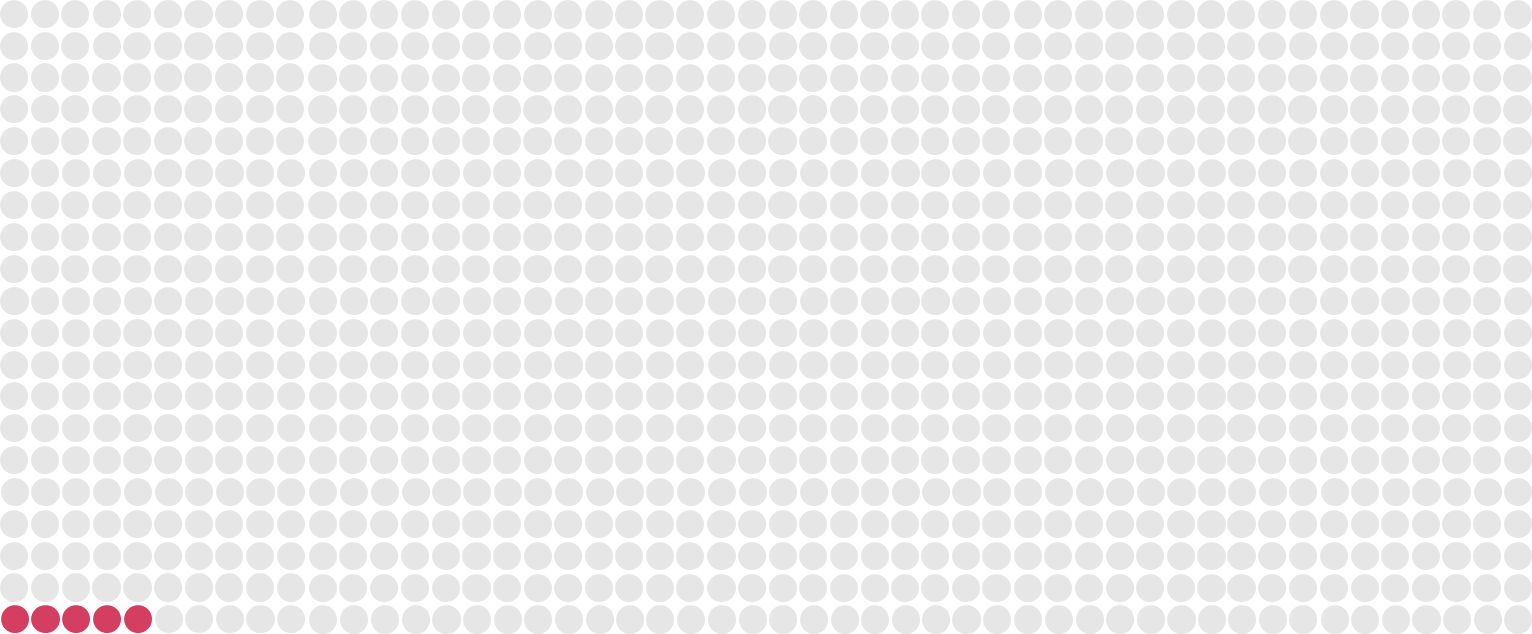 5 of 1,000 patients (0.5%) will develop *blood clots* \| \| --- \| --- \| |  | \| 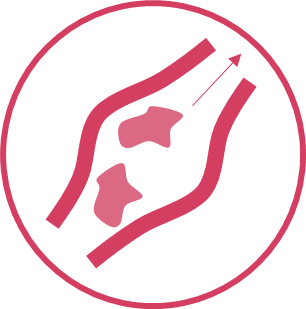 \| 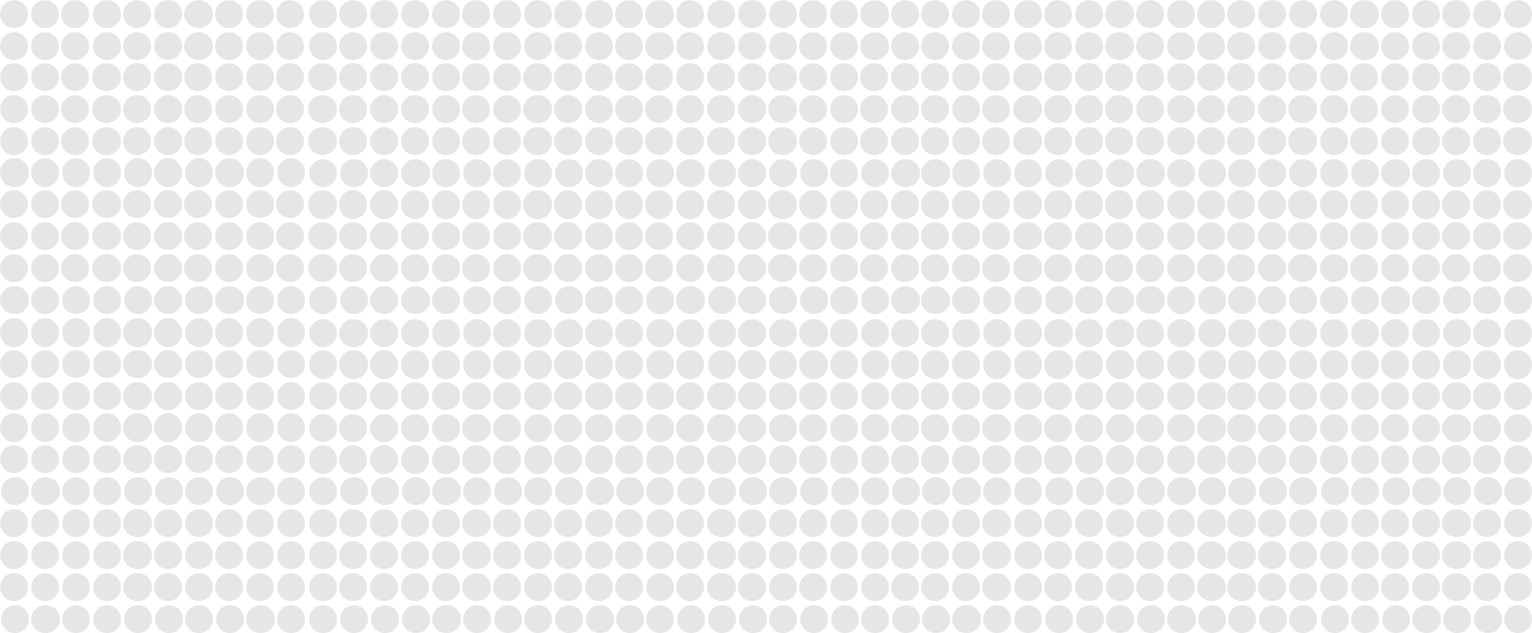 0 of 1,000 patients (0.0%) will develop *blood clots* \| \| --- \| --- \| |
| \| 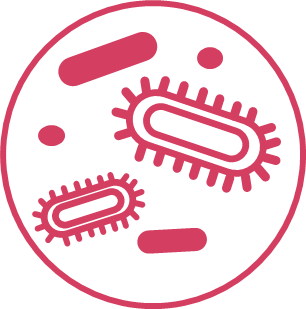 \| 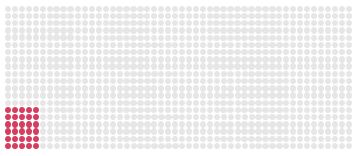 30 of 1,000 patients (3.0%)  will develop *serious infections* \| \| --- \| --- \| |  | \| 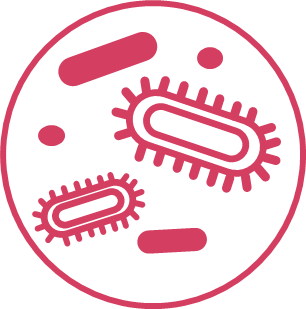 \| 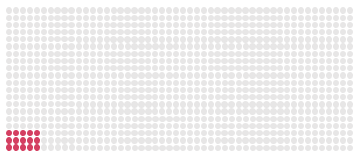 15 of 1,000 patients (4.0%)  will develop *serious infections* \| \| --- \| --- \| |
| \| 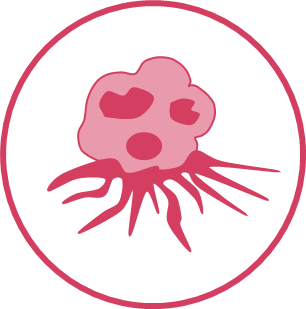 \| 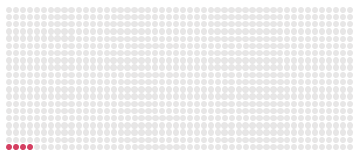 4 of 1,000 patients (0.4%)  will develop *cancer* \| \| --- \| --- \| |  | \| 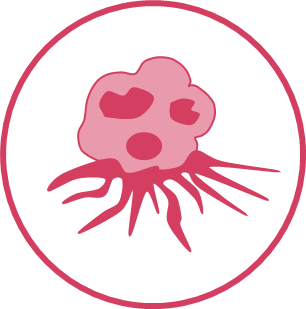 \| 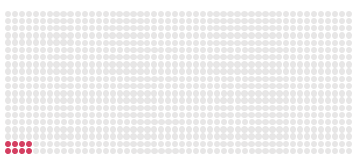 8 of 1,000 patients (0.8%)  will develop *cancer* \| \| --- \| --- \| |
| □ |  | □ |

**PROGRAMMER NOTE:**

**Qualitative Pilot:** Do not implement any randomization. Do not implement repeated task or dominant choice task. Only present the first 5 choice tasks.

**Main survey:** Implement repeated choice task, as seen by participant, and dominated choice task as final choice task. Implement all randomizations.

**DOMINANT CHOICE TASK**

| **Treatment A** |  | **Treatment B** |
| --- | --- | --- |
| \| 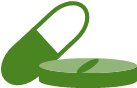 \| Oral pill at home every day \| \| --- \| --- \| |  | \| 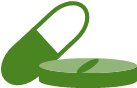 \| Oral pill at home every day \| Self-injection  at home every 8 weeks \| \| --- \| --- \| --- \| |
| \| 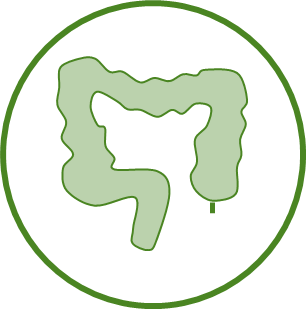 \| 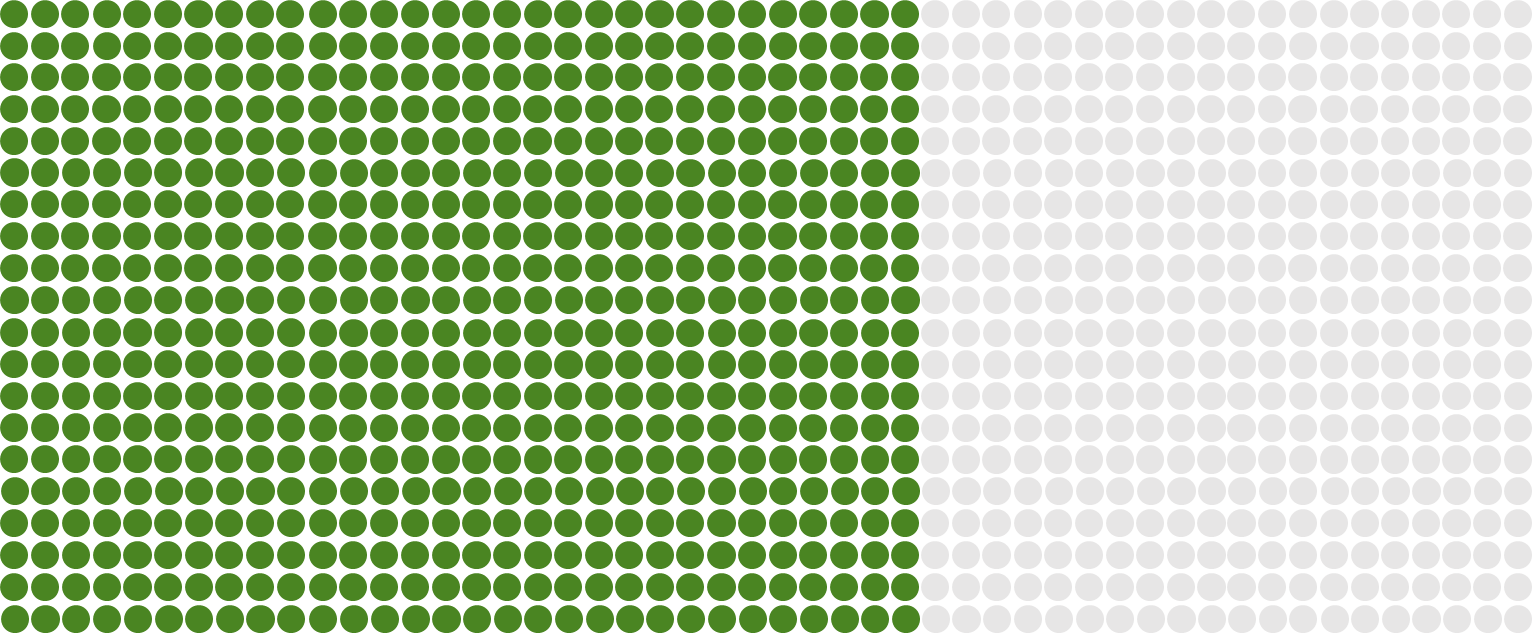 600 of 1,000 patients (60%) will  *achieve and maintain remission* \| \| --- \| --- \| |  | \| 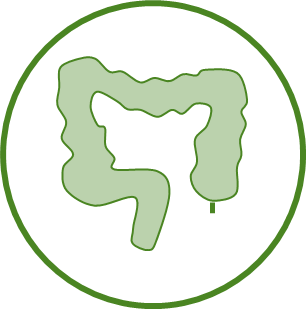 \| 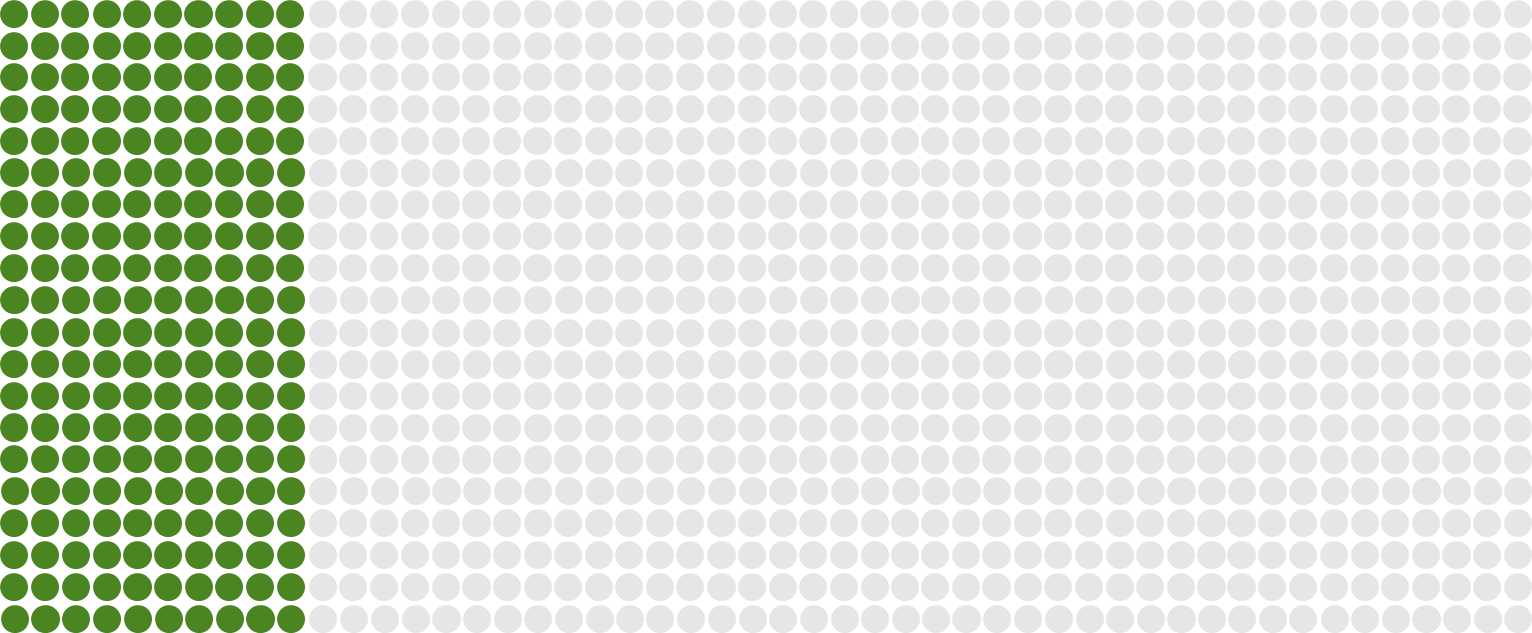 200 of 1,000 patients (20%) will  *achieve and maintain remission* \| \| --- \| --- \| |
| 0 episodes (courses) of *steroids* within a year |  | 3 to 4 episodes (courses) of *steroids*  within a year |
| \| 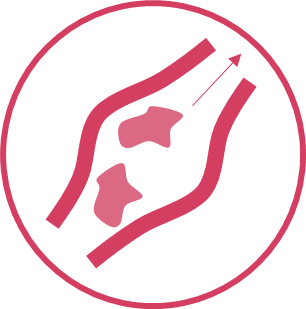 \| 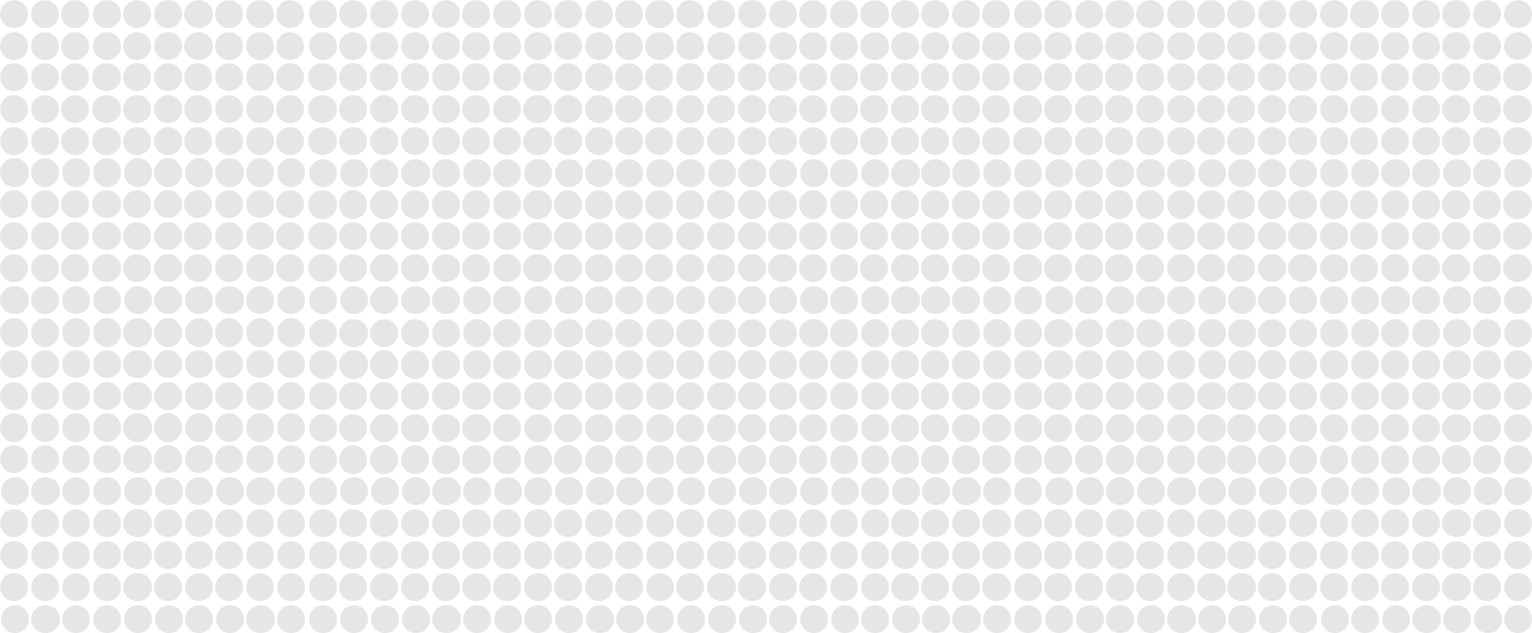 0 of 1,000 patients (0.0%) will develop *blood clots* \| \| --- \| --- \| |  | \| 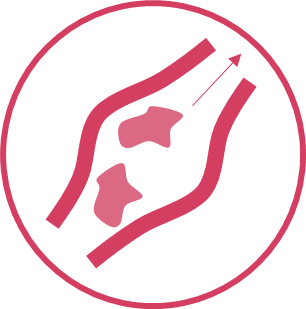 \| 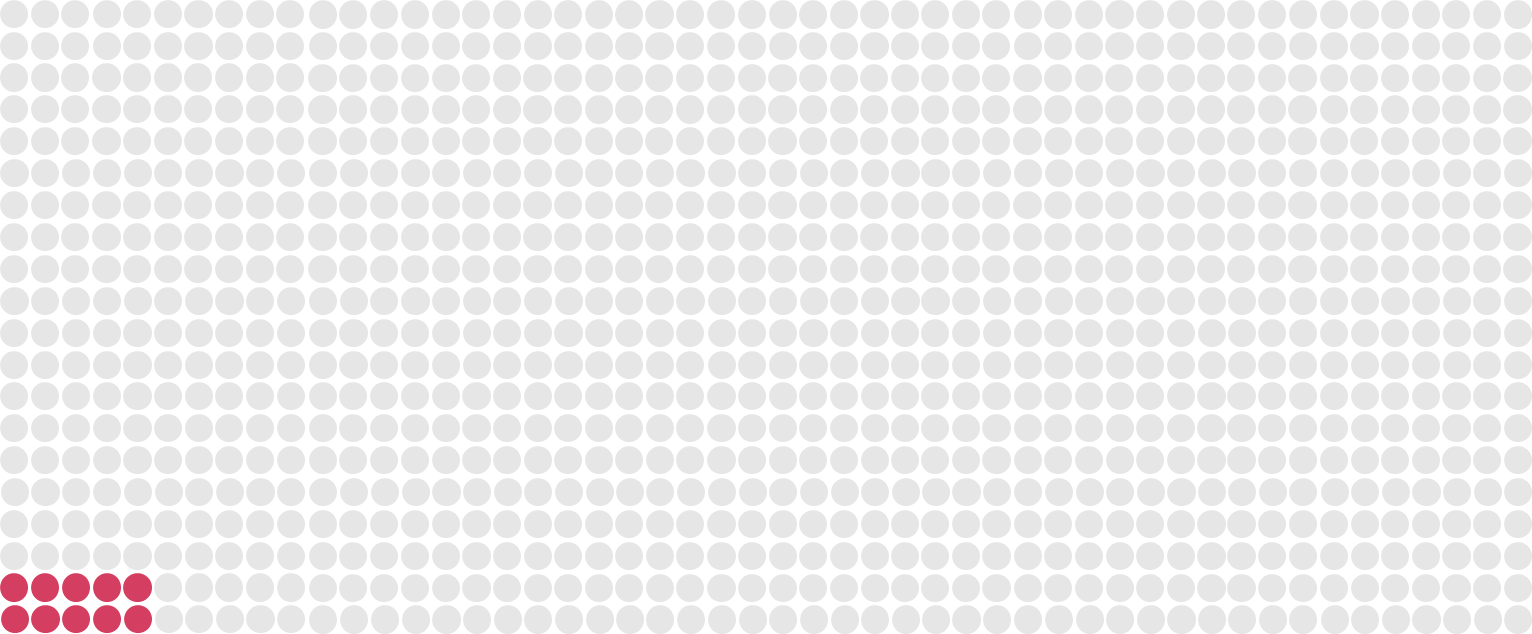 10 of 1,000 patients (1.0%) will develop *blood clots* \| \| --- \| --- \| |
| \| 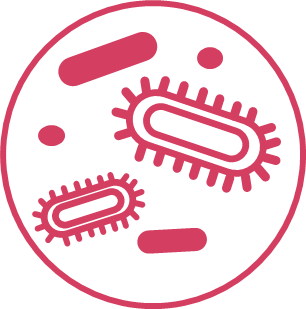 \| 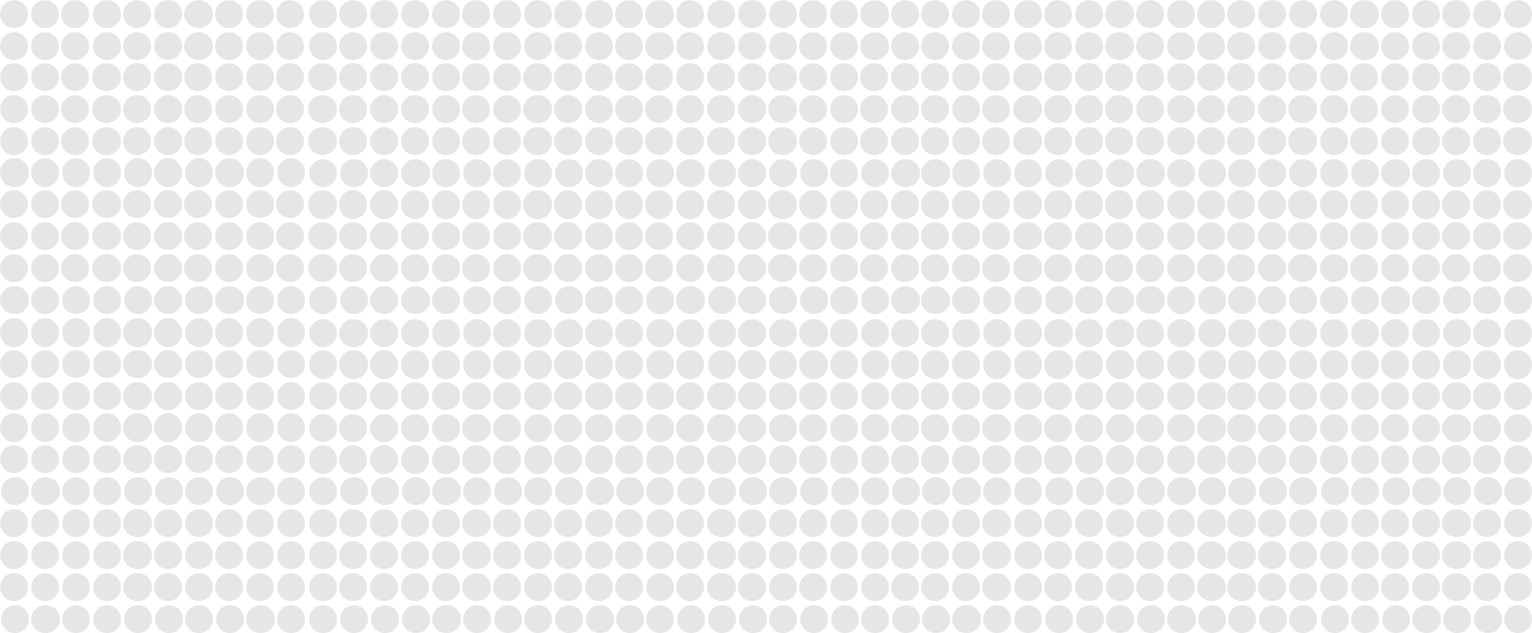 0 of 1,000 patients (0.0%) will develop *serious infections* \| \| --- \| --- \| |  | \| 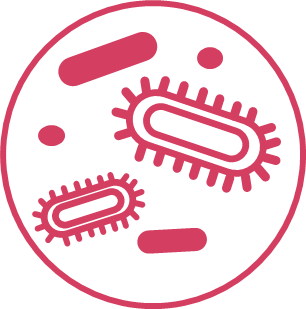 \| 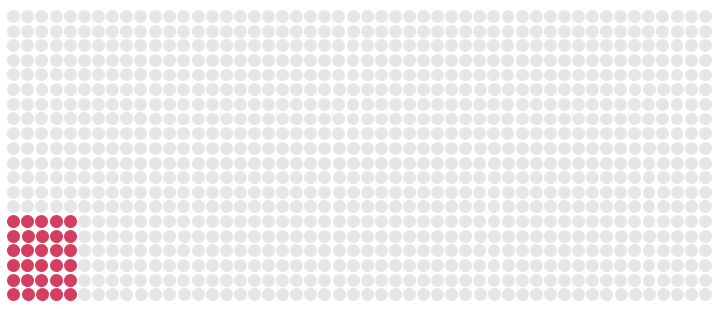 30 of 1,000 patients (3.0%) will develop *serious infections* \| \| --- \| --- \| |
| \| 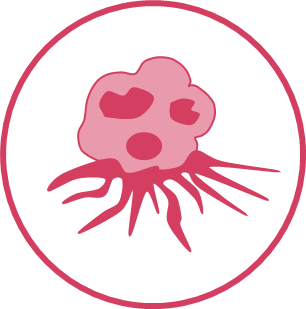 \| 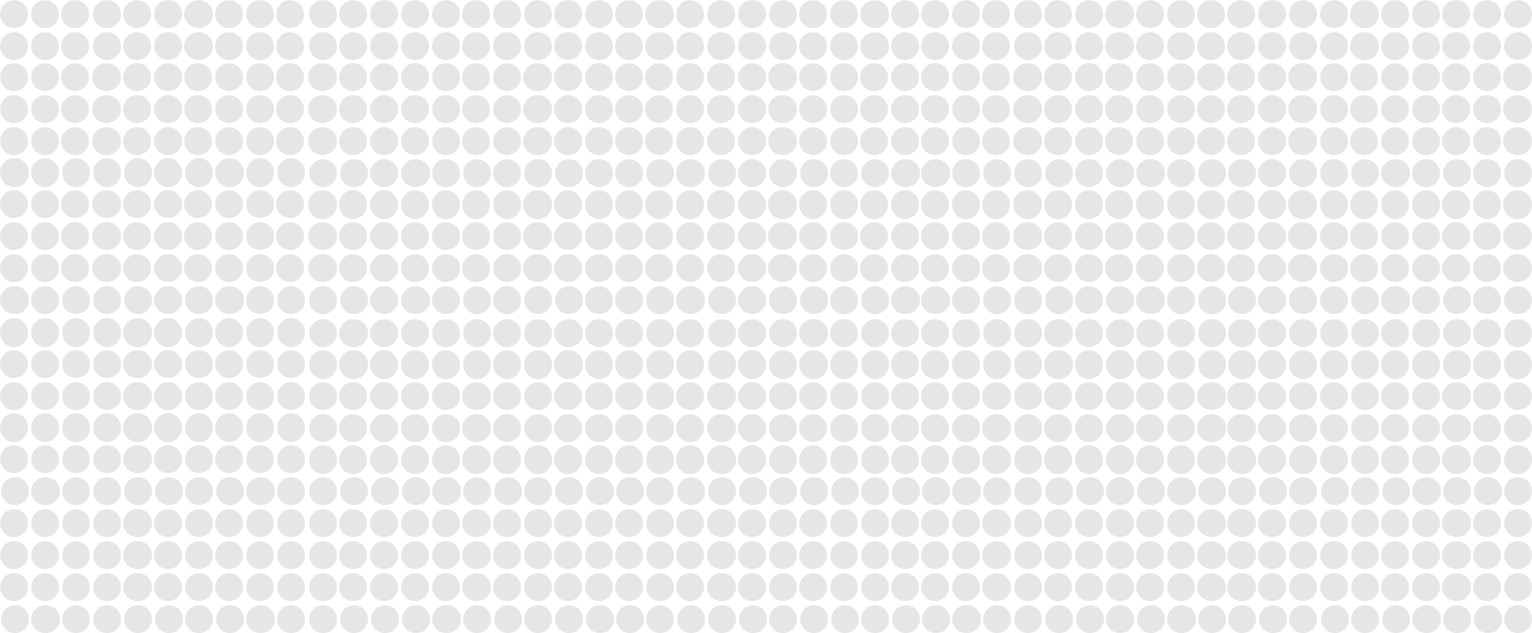 0 of 1,000 patients (0.0%) will develop *cancer* \| \| --- \| --- \| |  | \| 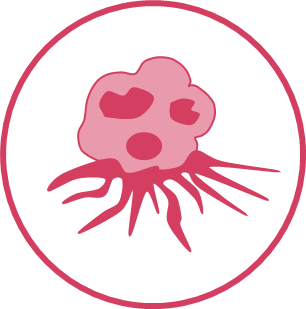 \| 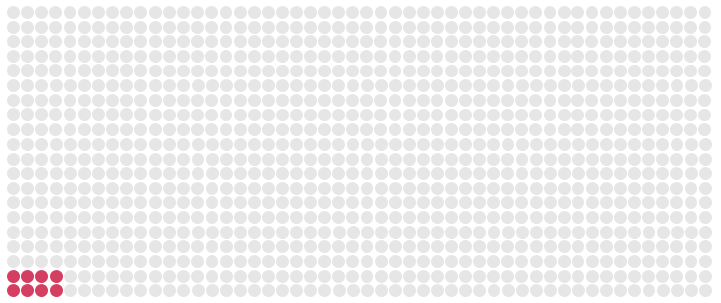 8 of 1,000 patients (0.8%)  will develop *cancer* \| \| --- \| --- \| |
| □ |  | □ |

**Within the previous choices you have been asked to select the preferred treatment out of two alternatives, while considering multiple treatment outcomes. Now, we will ask you *[INSERT FINAL NUMBER OF BWS CHOICE TASKS]* times to select the most and least important aspects from the list below. *Assuming you are currently in remission*, what are the most and least important UC-related aspects that you want a treatment to improve?**

**PROGRAMMER NOTE:**

**Qualitative Pilot:** Do not implement any randomization. Only present the first 3 choice tasks.

**Main survey:** Implement all randomizations.

| **Section 3: About you** |
| --- |

These first few questions will help us to understand your comfort level with written medical information.

*Select one response per question*

1. **How often do you have someone help you read medical information materials?**

| □ | □ | □ | □ | □ |
| --- | --- | --- | --- | --- |
| Always | Often | Sometimes | Occasionally | Never |

1. **How confident are you filling out medical forms by yourself?**

| □ | □ | □ | □ | □ |
| --- | --- | --- | --- | --- |
| Extremely | Quite a bit | Somewhat | A little bit | Not at all |

1. **How often do you have problems learning about your medical condition because of difficulty understanding written information?**

| □ | □ | □ | □ | □ |
| --- | --- | --- | --- | --- |
| Always | Often | Sometimes | Occasionally | Never |

**WEB PAGE BREAK**

Medical decisions often include information on probabilities and percentages. The following questions will help us understand how familiar and comfortable you are with this type of information.

1. **Which of the following numbers represents the highest probability/likelihood of getting a disease?**

*Select one option*

| □ | □ | □ |
| --- | --- | --- |
| 1 in 100 | 1 in 1000 | 1 in 10 |

1. **Which of the following numbers represents the highest probability/likelihood of getting a disease?**

*Select one option*

| □ | □ | □ |
| --- | --- | --- |
| 1% | 10% | 5% |

1. **If the chance of getting a disease is 10%, how many people would be expected to get the disease out of 100?**

*Select one option*

**[ENTER A NUMBER, RANGE 0-9999]**

1. **If the chance of getting a disease is 10%, how many people would be expected to get the disease out of 1000?**

*Select one option*

**[ENTER A NUMBER, RANGE 0-9999]**

1. **If the chance of getting a disease is 20 out of 100, this would be the same as having a ____% chance of getting the disease.**

*Select one option*

**WEB PAGE BREAK**

1. **How long ago were you diagnosed with UC?** *Select one option*

- Less than 6 months ago
- 6 months to less than 1 year ago
- 1 year to less than 3 years ago
- 3 years to less than 5 years ago
- 5 years to less than 10 years ago
- 10 years or more ago

1. **People with UC can experience a variety of different symptoms. Which symptoms have you experienced due to your UC?**

- Fatigue
- Abdominal pain
- Bloating and distention
- Rectal bleeding
- Stool frequency
- Stool urgency
- Constipation
- Tenesmus (the persistent urge to defecate without producing faeces)
- Rectal pain
- Diarrhea
- Faecal incontinence
- Night waking and insomnia

1. **Please arrange and rank the symptoms that you have experienced due to your UC according to the impact that they have on your quality of life. Place the symptom with the largest impact on your quality of life at the top, and then arrange the rest of the symptoms in descending order, from the greatest impact on your quality of life, to the smallest impact on quality of life.**

*Please use the drag and drop function*

**PROGRAMMER NOTE: THE LIST ON THE LEFT SHOULD BE POPULATED WITH THE ITEMS THAT ARE SELECTED IN Q=2. PARTICIPANTS MUST BE ABLE TO DRAG AND DROP SYMPTOMS INTO THE COLUMN ON THE RIGHT IN ORDER TO RANK THEM. PARTICPANTS MUST BE ABLE TO REORDER AND MOVE ITEMS UP AND DOWN.**

| *List of symptoms* | **Largest impact on quality of life** |
| --- | --- |
|  |  |
|  | **Smallest impact on quality of life** |

1. **Many patients do experience anxiety due to their UC. Please arrange and rank the symptoms that you have experienced due to your UC according to the impact that they have on your anxiety. Place the symptom with the largest impact on your anxiety at the top, and then arrange the rest of the symptoms in descending order, from the greatest impact on your anxiety, to the smallest impact on anxiety.**

*Please use the drag and drop function*

**PROGRAMMER NOTE: THE LIST ON THE LEFT SHOULD BE POPULATED WITH THE ITEMS THAT ARE SELECTED IN Q=2. PARTICIPANTS MUST BE ABLE TO DRAG AND DROP SYMPTOMS INTO THE COLUMN ON THE RIGHT IN ORDER TO RANK THEM. PARTICPANTS MUST BE ABLE TO REORDER AND MOVE ITEMS UP AND DOWN.**

| *List of symptoms* | **Makes me MOST anxious** |
| --- | --- |
|  |  |
|  | **Makes me LEAST anxious** |

**OR**

- My UC symptoms do not affect my feeling of anxiety

1. **How often do you feel anxious or find yourself worrying about your UC diagnosis or symptoms?**

- Never
- Almost never
- Sometimes
- Often
- All the time

1. **How often do you feel unhappy or depressed about your UC diagnosis or symptoms?**

- Never
- Almost never
- Sometimes
- Often
- All the time

1. **Thinking about how easy or difficult it is for you to get around by yourself outside your house (e.g. shopping)**

- Getting around is enjoyable and easy
- I have no difficulty getting around outside my house
- A little difficulty
- Moderate difficulty
- A lot of difficulty
- I cannot get around unless somebody is there to help me

1. **All treatments for UC can cause side effects. Potential side effects include those listed below. Please select the five side effects that you consider to be the worst, even if you have not experienced them before.** *Please select the five worst side effects, based on your own opinion.*

- Headache
- Diarrhea
- Nausea
- Joint Pain
- Vomiting
- Abdominal Pain
- Loss of appetite
- Rash
- Upper respiratory infections
- Weight gain
- Swelling
- Urinary tract infection
- Viral infection
- Tired/Fatigue
- Aesthetic complications (e.g., acne, increased size in the face and neck, etc.)
- Insomnia
- Depression
- Anxiety
- Cancer

1. **During the previous week, did you make adjustments to your activities to ensure there was a toilet nearby?**

- Yes
- No
- I don’t know

1. **What aspects of your life have been impacted by UC and/or treatment?**

*Select all that apply*

- Physical exercise (e.g. sport, walking)
- Household chores (e.g. cleaning, shopping)
- Social aspects (e.g. attending social events)
- Family responsibilities (e.g. caring for family, child-minding)
- Emotional aspects (e.g., reducing burden and worries about ulcerative colitis)
- Financial and work aspects (e.g., being able to work, earning an income)

1. **Please arrange and rank the aspects of your like that have been impacted by UC according to the impact that they have on your quality of life. Place the item with the largest impact on your quality of life at the top, and then arrange the rest of the symptoms in descending order, from the greatest impact on your quality of life, to the smallest impact on quality of life.**

*Please use the drag and drop function*

**PROGRAMMER NOTE: THE LIST ON THE LEFT SHOULD BE POPULATED WITH THE ITEMS THAT ARE SELECTED IN Q=2. PARTICIPANTS MUST BE ABLE TO DRAG AND DROP SYMPTOMS INTO THE COLUMN ON THE RIGHT IN ORDER TO RANK THEM. PARTICPANTS MUST BE ABLE TO REORDER AND MOVE ITEMS UP AND DOWN.**

| *List of Activities* | **Largest impact on quality of life** |
| --- | --- |
|  |  |
|  | **Smallest impact on quality of life** |

1. **We would like to know how good or bad your overall health is today. 1 means the worst overall health you can imagine. 100 means the best overall health you can imagine.**

*Indicate on the scale how your overall health is today.*


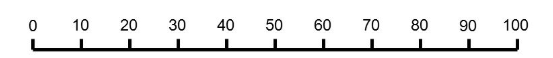


1. **We would like to know how good or bad your abdominal pain is today. 1 means no pain. 100 means the worst pain you can imagine.**

*Indicate on the scale how your pain is today.*


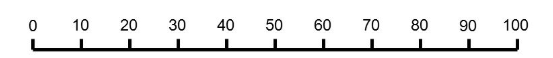


1. **Treatments can improve symptoms of ulcerative colitis which will also improve aspects of your daily living. Which aspects of daily living are important to you?**

*Select all that apply*

- Physical aspects (e.g., being able to do exercise, do household chores and physical activities)
- Social aspects (e.g., spending time with family and friends)
- Emotional aspects (e.g., reducing burden and worries about ulcerative colitis)
- Financial and work aspects (e.g., being able to work, earning an income)

**Q21 [UK only:] What is your racial background?**

*Select all that apply*

| UK |
| --- |
| □ White  □ Black/African/Caribbean/Black-British  □ Asian or Asian-British  □ Other (please specify)  □ Prefer not to say |

**Q22 What is the highest level of education you have completed?**

*Select one answer*

| UK | FR | DE | ES | IT |
| --- | --- | --- | --- | --- |
| No formal qualifications | No formal qualification | Grundschule | No formal qualifications | No formal qualifications |
| GCSE/ O’ levels or equivalent | Primary school | Sonderschule | Primary School | Primary School |
| A’ levels or equivalent | Faculty | Hauptschule | Secondary School | Secondary School |
| Vocational/ work-based qualifications | Baccalaureate | Realschule | High School/Vocational Training | Upper Secondary School |
| Undergraduate degree | Undergraduate degree | Abitur | Undergraduate degree | Undergraduate degree |
| Graduate degree | Graduate degree | Berufsausbildung | Graduate degree | Graduate degree |
| PhD/MBA | PhD/MBA | Bachelor | PhD/MBA | PhD/MBA |
| Other: ________ | Other: ________ | Master/Diplom | Other: ________ | Other: ________ |
|  |  | Promotion/MBA |  |  |
|  |  | Other: ________ |  |  |

**Q23 Which of the following best describe your employment status?**

*Select one answer*

□ Employed, full time

□ Employed, part time

□ Self-employed

□ Voluntary work

□ Homemaker

□ Student

□ Unemployed

□ Retired

□ On sick leave

□ Maternity/paternity leave

□ Not able to work due to disability

**Q24 What health insurance do you currently have?**

*Select all that apply*

| UK | FR | DE | ES | IT |
| --- | --- | --- | --- | --- |
| National Health Service | National Health Service | Gesetzliche Krankenversicherung | National Health Service | National Health Service |
| Employer provided private insurance | Employer provided private insurance | Private Krankenversicherung | Employer provided private insurance | Employer provided private insurance |
| Personal provided private insurance | Personal provided private insurance |  | Personal provided private insurance | Personal provided private insurance |

**WEB PAGE BREAK**

**Thank you for participating in this survey!**

**Supplementary Table 1.** Benefits of medical treatments for UC extracted from evidence review.

|  | **Quantitative studies** | | | | | | | | | | | | **Qualitative studies** | | | | | | **RCT** |
| --- | --- | --- | --- | --- | --- | --- | --- | --- | --- | --- | --- | --- | --- | --- | --- | --- | --- | --- | --- |
|  | **Gregor *et al.* 2018^7^** | **Bewtra *et al.* 2015^8^** | **Almario *et al.* 2018^9^** | **Boeri *et al.* 2019^10^** | **MacKenzie-Smith *et al.* 2018^11^** | **Hazlewood *et al.* 2020^12^** | **Hagelund *et al.* 2020^13^** | **Holko *et al.* 2018^14^** | **van Deen *et al.* 2017^15^** | **Bewtra *et al.* 2020^16^** | **Casellas *et al.* 2017^17^** | **Living with UC^a^** | **McMullan *et al.* 2017^18^** | **Rubin *et al.* 2020^19^** | **Higgins *et al.* 2017^20^** | **Rapport *et al.* 2019^21^** | **van Waal *et al.* 2019^22^** | **Walsh *et al.* 2019^23^** | **NCT02914522^24, 25^** |
| **Population** | **UC/ CD** | **UC/ CD** | **UC/ CD** | **UC** | **UC** | **UC** | **UC** | **UC/ CD^b^** | **UC/ CD** | **CD** | **UC/ CD** | **UC** | **UC** | **UC** | **UC** | **UC** | **UC** | **UC** | **UC** |
| Symptom relief at 12 months | ● |  | ● | ● |  |  | ● |  |  |  |  |  |  |  |  |  |  |  |  |
| Mucosal healing at 12 months | ● |  |  |  |  |  |  |  |  |  |  |  |  |  |  |  |  |  |  |
| Remission at 12 months |  |  |  |  |  | ● |  |  |  |  |  |  |  |  |  |  |  |  | ● |
| Symptom improvement^c^ |  |  | ● |  |  |  |  |  |  |  | ● | ● |  |  | ● |  | ● |  | ● |
| Time until symptoms improve | ● |  | ● | ● |  |  |  |  |  |  |  | ● |  |  |  | ● |  |  |  |
| Control of inflammation |  |  |  |  |  |  |  |  | ● |  |  |  |  |  |  |  |  |  |  |
| HRQoL improvement |  |  |  |  |  |  |  |  | ● |  |  | ● | ● | ● |  |  | ● | ● |  |
| Productivity (home, work etc.) |  |  |  |  |  |  |  |  | ● |  |  | ● | ● |  |  | ● |  | ● |  |
| Probability of needing surgery |  |  |  |  |  |  |  |  | ● |  |  | ● |  |  |  | ● |  |  |  |
| Hospitalization in the next year | ● |  |  |  |  |  |  |  |  |  |  |  |  |  |  |  |  |  |  |
| Time with specified severity |  |  |  |  |  |  |  |  | ● |  |  |  |  |  |  |  |  |  |  |
| Time in remission |  |  |  |  |  |  |  |  |  |  |  | ● |  |  |  |  |  |  |  |

^a^Living with UC: EU5 results, Research Partnership Ltd, March 2020.

^b^n = 53 CD and n = 74 ‘other’ type of IBD, including UC.

^c^Includes rectal bleeding, urge to have bowel movement, diarrhoea, distention/meteorism/bloating, abdominal pain, incontinence, articular pain, night waking, stool frequency, fatigue, rectal pain; defined as ‘risk of illness’.

CD, Crohn’s disease; EU5, European Union 5; IBD, inflammatory bowel disease; HRQoL, health-related quality of life; RCT, randomized controlled trial; UC, ulcerative colitis.

**Supplementary Table 2.** Risks associated with medical treatments for UC extracted from evidence review.

|  | **Quantitative studies** | | | | | | | | | | | | **Qualitative studies** | | | | | | | | | | | **RCT** |
| --- | --- | --- | --- | --- | --- | --- | --- | --- | --- | --- | --- | --- | --- | --- | --- | --- | --- | --- | --- | --- | --- | --- | --- | --- |
|  | **Gregor *et al.* 2018^7^** | **Bewtra *et al.* 2015^8^** | **Almario *et al.* 2018^9^** | **Boeri *et al.* 2019^10^** | **MacKenzie-Smith *et al.* 2018^11^** | **Hazlewood *et al.* 2020^12^** | **Hagelund *et al.* 2020^13^** | **Holko *et al.* 2018^14^** | **van Deen *et al.* 2017^15^** | **Bewtra *et al.* 2020^16^** | **Casellas *et al.* 2017^17^** | **Living with UC^a^** | **McMullan *et al.* 2017^18^** | **Rubin *et al.* 2020^19^** | | **Higgins *et al.* 2017^20^** | | **Rapport *et al.* 2019^21^** | | **van Waal *et al.* 2019^22^** | | **Walsh *et al.* 2019^23^** | | **NCT-02914522^24, 25^** |
| **Population** | **UC/ CD** | **UC/ CD** | **UC/ CD** | **UC** | **UC** | **UC** | **UC** | **UC/ CD^b^** | **UC/ CD** | **CD** | **UC/ CD** | **UC** | **UC** | | **UC** | | **UC** | | **UC** | | **UC** | | **UC** | **UC** |
| Need for steroids | ● |  |  | ● |  | ● |  |  |  | ● |  | ● |  | |  | |  | |  | |  | |  |  |
| Pain during administration | ● |  |  |  |  |  |  |  |  |  |  |  |  | |  | |  | |  | |  | |  |  |
| Injection/infusion reaction | ● |  |  |  |  |  |  | ● |  |  |  |  |  | |  | |  | | ● | |  | |  |  |
| Lymphoma/malignancy |  | ● | ● | ● |  | ● |  |  |  | ● |  |  |  | | ● | |  | |  | |  | |  | ● |
| Infections (including herpes zoster) |  | ● | ● | ● |  | ● |  |  |  | ● |  | ● |  | | ● | |  | |  | |  | |  | ● |
| Number of months to relapse |  | ● |  |  |  |  |  |  |  |  |  |  |  | |  | |  | |  | |  | |  |  |
| Discontinuing due to side effects |  |  | ● |  |  | ● |  |  |  |  |  |  |  | |  | |  | |  | |  | |  |  |
| Rash |  |  | ● |  |  |  |  |  |  |  |  |  |  | |  | |  | |  | |  | |  |  |
| Low blood count or liver reaction |  |  |  |  |  | ● |  |  |  |  |  | ● |  | |  | |  | |  | |  | |  | ● |
| Fever/feeling hot |  |  |  |  |  |  |  |  |  |  |  |  |  | |  | |  | | ● | |  | |  | ● |
| Blood clots |  |  |  |  |  |  |  |  |  |  |  | ● |  | |  | |  | |  | |  | |  | ● |
| Leg sensations (tingling, numbness) |  |  |  |  |  |  |  |  |  |  |  |  |  | |  | |  | | ● | |  | |  |  |
| Headache |  |  |  |  |  |  |  |  |  |  |  | ● |  | |  | |  | | ● | |  | |  | ● |
| Upper respiratory tract symptoms |  |  |  |  |  |  |  |  |  |  |  |  |  | |  | |  | |  | |  | |  | ● |
| Nausea/vomiting |  |  |  |  |  |  |  |  |  |  |  | ● |  | |  | |  | |  | |  | |  | ● |

^a^Living with UC: EU5 results, Research Partnership Ltd, March 2020.

^b^n = 53 CD and n = 74 “other” type of IBD, including UC.

CD, Crohn’s disease; EU5, European Union 5; IBD, inflammatory bowel disease; RCT, randomized controlled trial; UC, ulcerative colitis.

**Supplementary Table 3.** Best–worst scaling attributes.

*Assuming you are currently in remission, what are the most and least important ulcerative colitis (UC) related aspects that you want a treatment to improve?*

| **Most important** | **Attribute** | **Least important** |
| --- | --- | --- |
|  | My treatment gives me **more** **energy** |  |
|  | My treatment helps me feel less **worried or anxious** about my UC |  |
|  | My treatment helps me be able to attend **school or work** |  |
|  | My treatment helps me attend **social engagements** such as parties or gatherings with friends and family |  |
|  | My treatment helps me participate in **sports and leisure** activities |  |
|  | My treatment helps me get a **good full-night sleep** |  |
|  | My treatment helps me be able to do **daily activities** like household chores |  |
|  | My treatment helps me **maintain healthy** **sexual relationships** |  |

UC, ulcerative colitis.

**Supplementary Table 4.** Values and relevant clinical data sources used to describe the filgotinib 200 mg and placebo treatment profiles.

| **Treatment attribute** | **Filgotinib 200 mg** | **Placebo** | **Clinical data source** |
| --- | --- | --- | --- |
| Likelihood of achieving and maintaining remission | 37.2% | 12.3% | The maintenance study of the filgotinib phase 2b/3 trial (NCT02914522).^25^ |
| Risk of serious infections | 1.3% | 1.1% | The maintenance study of the filgotinib phase 2b/3 trial (NCT02914522).^25^ |
| Number of episodes (courses) of steroids per year | Assumed to be constant across both filgotinib and placebo. | | |
| Risk of malignancies^a^ | 0.6% | 0.1% | An integrated safety analysis among patients with UC treated with tofacitinib.^26^ A small baseline risk was defined for placebo.^27, 28^ |
| Risk of blood clots^a^ | 0.2% | 0.1% | An integrated safety analysis among patients with UC treated with tofacitinib.^26^ A small baseline risk was defined for placebo.^29^ |

^a^Given the challenges of reliably estimating the malignancy and blood clot risk, the maintenance study of the filgotinib phase 2b/3 trial was not used in this instance.

UC, ulcerative colitis.

**Supplementary Table 5.** Model Parameters.

| **Attribute** | **Levels** | **Variable name** | **Paramete**r |
| --- | --- | --- | --- |
| **How and how often the treatment is taken** | Intravenous treatment in hospital/clinic every 4–8 weeks | Reference | |
|  | Self-injection at home every 1–2 weeks | $admin:si$ | *β_1_* |
|  | Oral pill at home every day | $admin:oral$ | *β_2_* |
| **Likelihood of achieving and maintaining remission** | 20% | Reference | |
|  | 40% | $remission:$  $40$ | *β_3_* |
|  | 60% | $remission:$  $60$ | *β_4_* |
| **Number of episodes of steroids per year** | 3 or 4 episodes (courses) of steroid use | Reference | |
|  | 1 or 2 episodes (courses) of steroid use | $steroid:$  $1 o r2$ | *β_5_* |
|  | No steroid use | $steroid:0$ | *β_6_* |
| **Risk of blood clots** | 1.0% (10 of 1,000 patients) | Reference | |
|  | 0.5% (5 of 1,000 patients) | $bloodclot:$  $0.5$ | *β_7_* |
|  | 0.0% (0 of 1,000 patients) | $bloodclot:$  $0.0$ | *β_8_* |
| **Risk of serious infections** | 3.0% (30 of 1,000 patients) | Reference | |
|  | 1.5% (15 of 1,000 patients) | $infections:$  $1.5$ | *β_9_* |
|  | 0.0% (0 of 1,000 patients) | $infections:$  $0.0$ | *β_10_* |
| **Risk of cancer** | 0.8% (8 of 1,000 patients) | Reference | |
|  | 0.4% (4 of 1,000 patients) | $cancer:0.4$ | *β_11_* |
|  | 0.0% (0 of 1,000 patients) | $cancer:$0.0 | *β_12_* |
| **Alternative-specific constant** | Left alternative (option A) | Reference | |
|  | Right alternative (option B) | *-* | $\alpha_{left}$ |

**Supplementary Figure 1.** Study design.


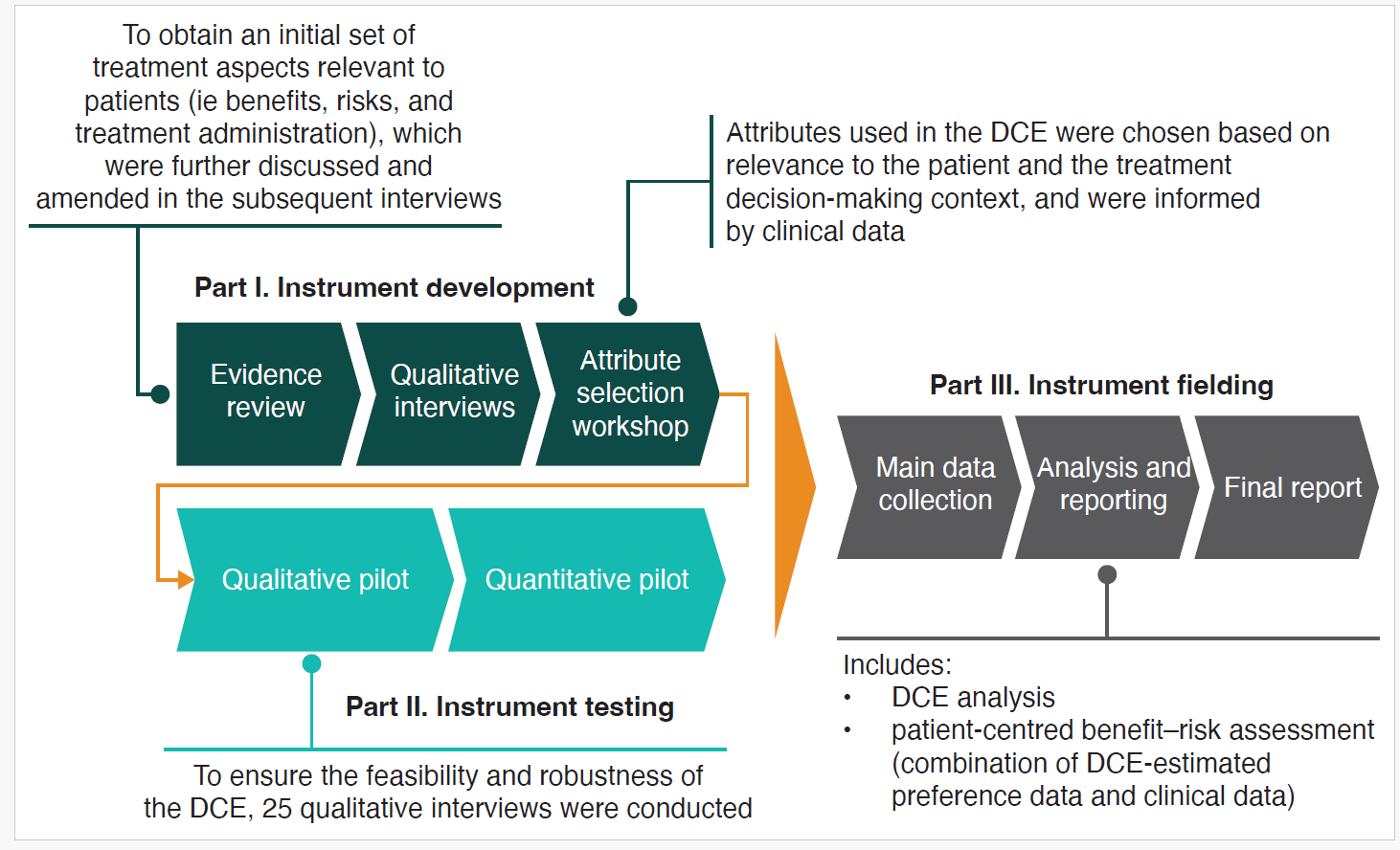


DCE, discrete choice experiment.

**Supplementary Figure 2.** Summary of patient-relevant treatment themes identified from qualitative research.


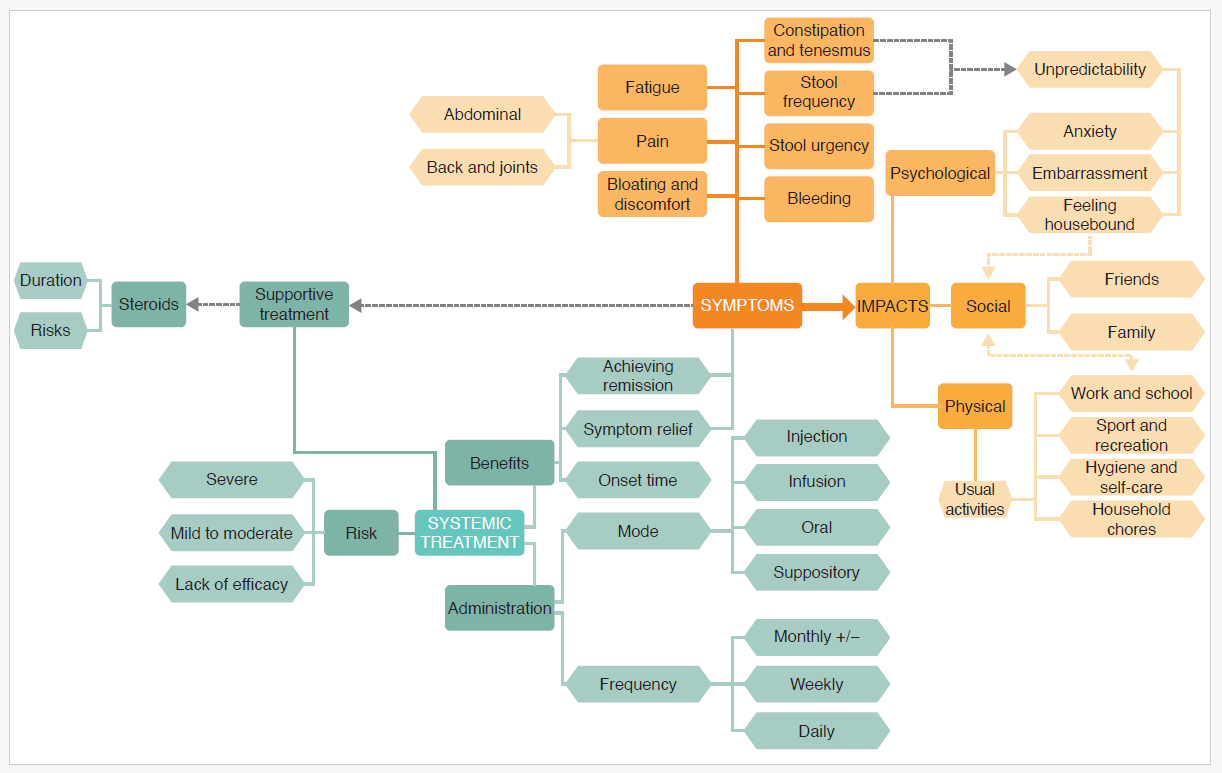


**Supplementary Figure 3.** Patient disposition.


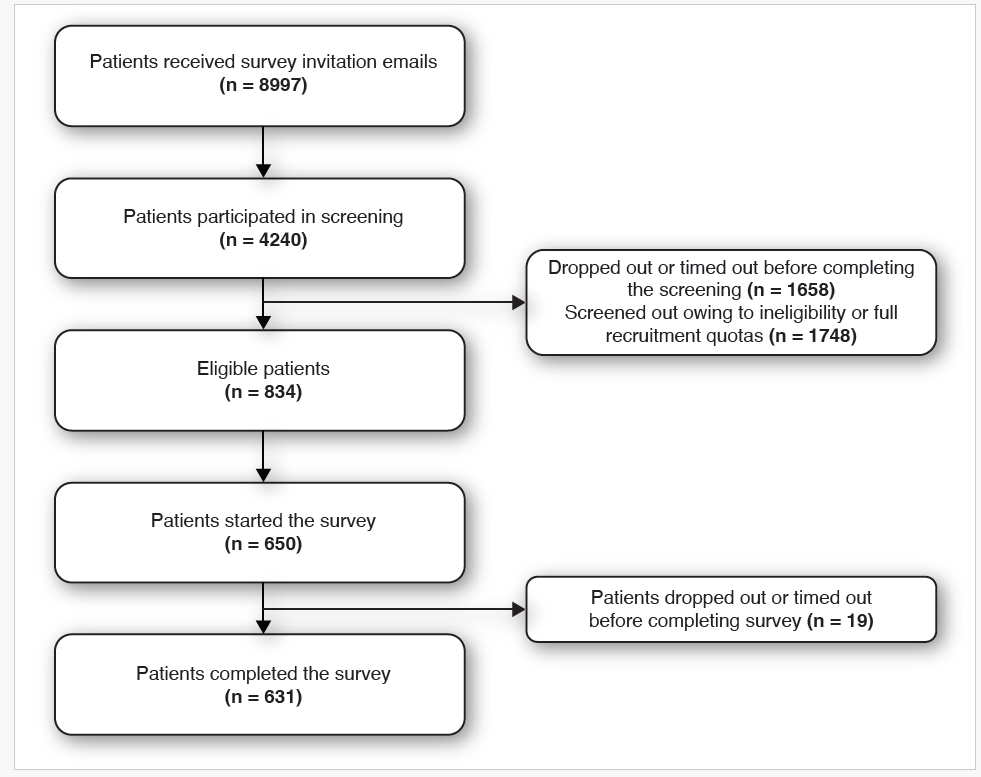


**Supplementary Figure 4.** Relative attribute importance in the overall population.

**
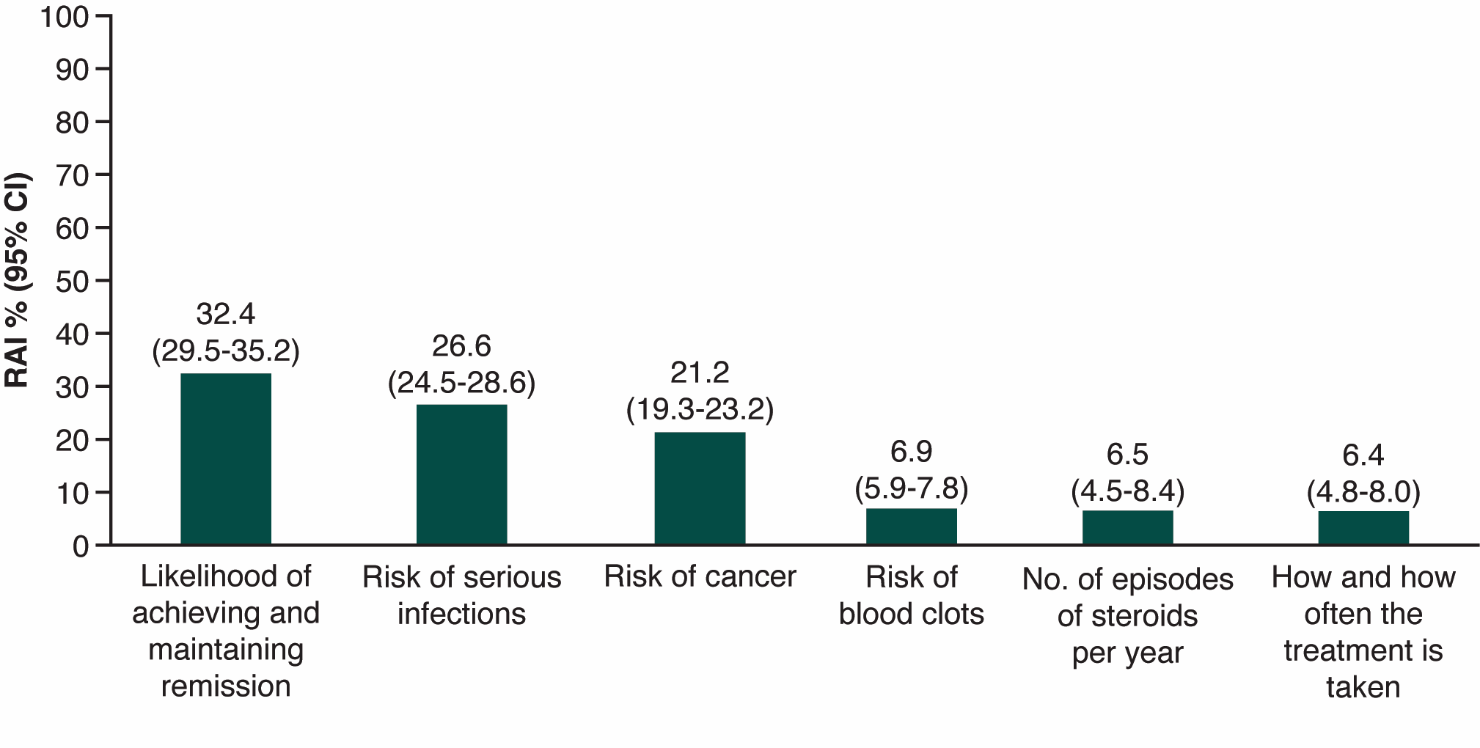
**

CI, confidence interval; RAI, relative attribute importance.

**Supplementary Figure** **5.** Best–worst scaling results.


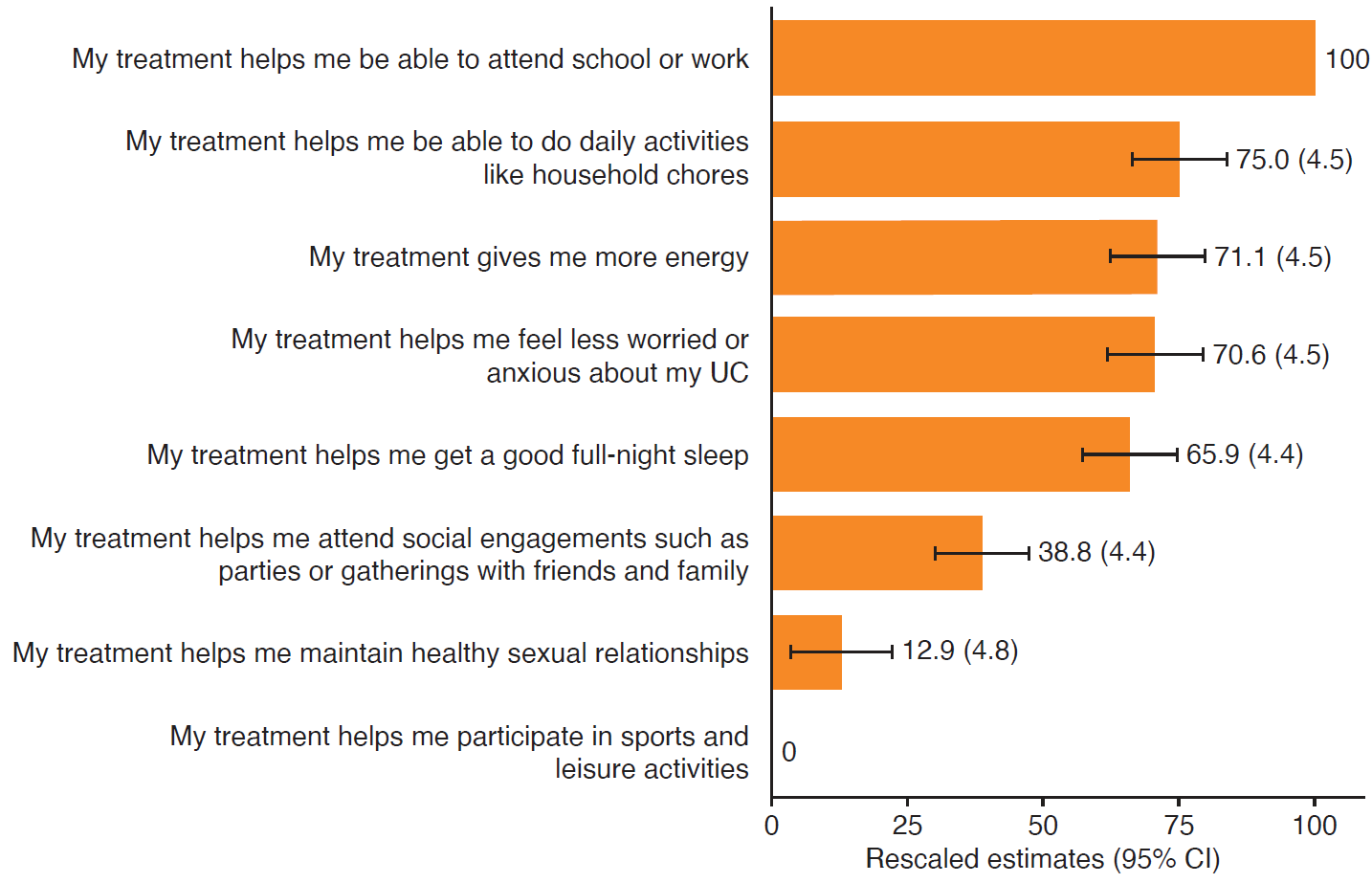


Log-likelihood at convergence, −969.7; BIC, 2411.9; adjusted McFadden pseudo-R^2^, 2.8%; total number of parameters: 7; estimates are normalized importance scores.

BIC, Bayesian information criterion; CI, confidence interval; UC, ulcerative colitis.

**Supplementary Figure 6.** Best–worst scaling results stratified by subgroup: (a) country of residence, (b) age, and (c) time since diagnosis.

**
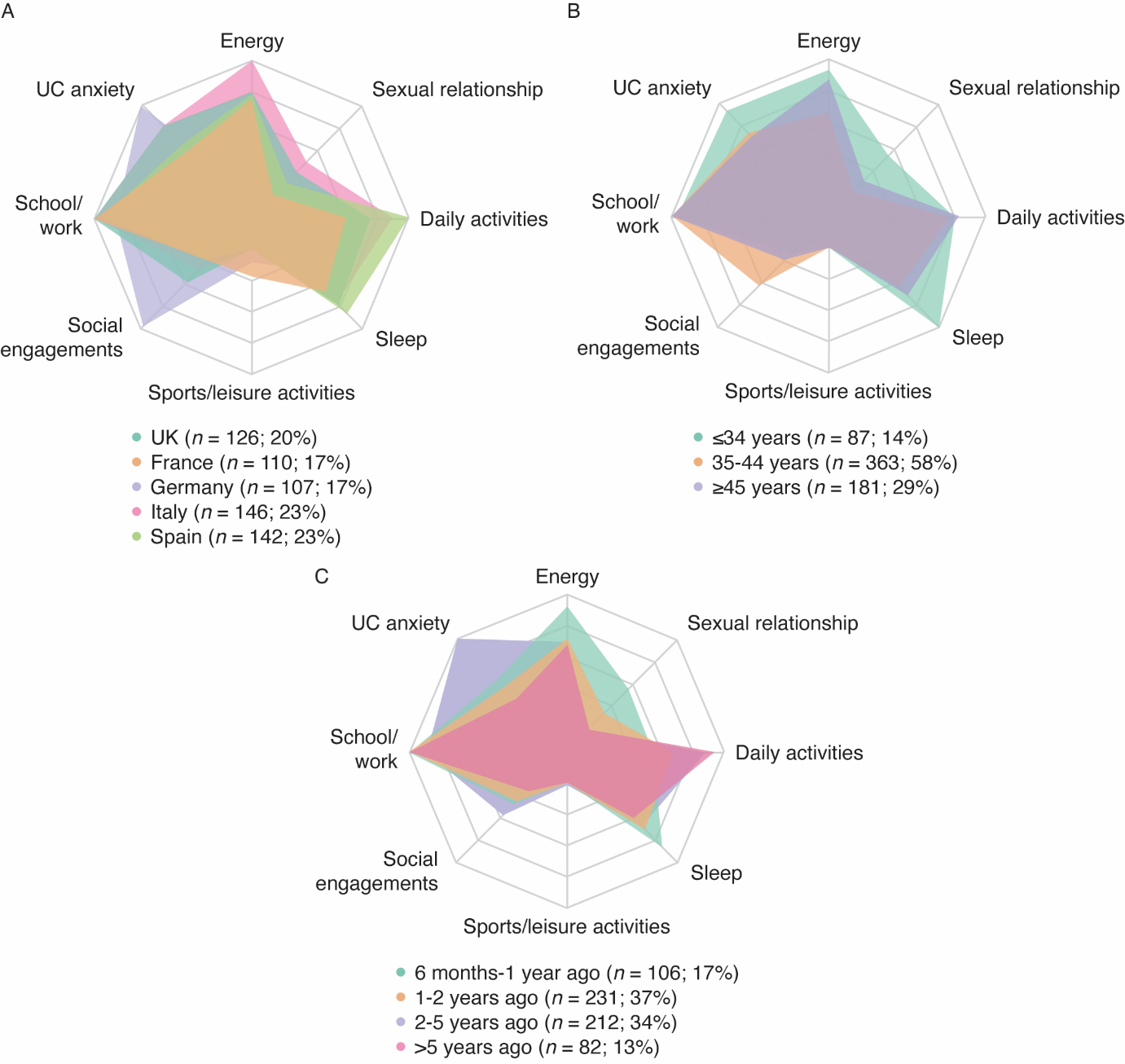
**

Radar plots denote relative importance weights obtained for subgroups from the best–worse scaling data. The outermost circle corresponds to a value of 1, while the innermost circle corresponds to a value of 0. Areas with little overlap indicate noticeable differences in treatment priorities among subgroups.

UC, ulcerative colitis.

**REFERENCES**

1 Manski CF. The structure of random utility models. *Theory and Decision*. 1977;8(3):229-254.

2 Binary Choice Constraints on Random Utility Indicators. Cowles Foundation for Research in Economics, Yale University. 1959. Accessed November 27, 2023. <https://EconPapers.repec.org/RePEc:cwl:cwldpp:74>

3 McFadden D. *Conditional logit analysis of qualitative choice behaviour*. New York, NY: Academic Press; 1974. 105-142

4 Thurstone LL. A law of comparative judgment. *Psychological Review*. 1927;34(4):273-286.

5 Hess S, Palma D. Apollo: A flexible, powerful and customisable freeware package for choice model estimation and application. *Journal of Choice Modelling*. 2019;32:100170.

6 Apollo version 0.1.0 user manual. Accessed November, 27 2023. <www.ApolloChoiceModelling.com>

7 Gregor JC, Williamson M, Dajnowiec D, Sattin B, Sabot E, Salh B. Inflammatory bowel disease patients prioritize mucosal healing, symptom control, and pain when choosing therapies: results of a prospective cross-sectional willingness-to-pay study. *Patient Prefer Adherence*. 2018;12:505-513.

8 Bewtra M, Fairchild AO, Gilroy E, et al. Inflammatory bowel disease patients' willingness to accept medication risk to avoid future disease relapse. *Am J Gastroenterol*. 2015;110(12):1675-1681.

9 Almario CV, Keller MS, Chen M, et al. Optimizing selection of biologics in inflammatory bowel disease: development of an online patient decision aid using conjoint analysis. *Am J Gastroenterol*. 2018;113(1):58-71.

10 Boeri M, Myers K, Ervin C, et al. Patient and physician preferences for ulcerative colitis treatments in the United States. *Clin Exp Gastroenterol*. 2019;12:263-278.

11 MacKenzie-Smith L, Marchi P, Thorne H, Timeus S, Young R, Le Calve P. Patient preference and physician perceptions of patient preference for oral pharmaceutical formulations: results from a real-life survey. *Inflamm Intest Dis*. 2018;3(1):43-51.

12 Hazlewood GS, Pokharel G, Deardon R, et al. Patient preferences for maintenance therapy in Crohn's disease: a discrete-choice experiment. *PLoS One*. 2020;15(1):e0227635.

13 Hagelund LM, Elkjaer Stallknecht S, Jensen HH. Quality of life and patient preferences among Danish patients with ulcerative colitis - results from a survey study. *Curr Med Res Opin*. 2020;36(5):771-779.

14 Holko P, Kawalec P, Mossakowska M. Quality of life related to oral, subcutaneous, and intravenous biologic treatment of inflammatory bowel disease: a time trade-off study. *Eur J Gastroenterol Hepatol*. 2018;30(2):174-180.

15 van Deen WK, Nguyen D, Duran NE, Kane E, van Oijen MG, Hommes DW. Value redefined for inflammatory bowel disease patients: a choice-based conjoint analysis of patients' preferences. *Qual Life Res*. 2017;26(2):455-465.

16 Bewtra M, Reed SD, Johnson FR, et al. Variation among patients with Crohn's disease in benefit vs risk preferences and remission time equivalents. *Clin Gastroenterol Hepatol*. 2020;18(2):406-414 e407.

17 Casellas F, Herrera-de Guise C, Robles V, Navarro E, Borruel N. Patient preferences for inflammatory bowel disease treatment objectives. *Dig Liver Dis*. 2017;49(2):152-156.

18 McMullan C, Pinkney TD, Jones LL, et al. Adapting to ulcerative colitis to try to live a 'normal' life: a qualitative study of patients' experiences in the Midlands region of England. *BMJ Open*. 2017;7(8):e017544.

19 Rubin DT, Hart A, Panaccione R, et al. Ulcerative colitis narrative global survey findings: communication gaps and agreements between patients and physicians. *Inflamm Bowel Dis*. 2020:1096–1106.

20 Higgins PDR, Harding G, Revicki DA, et al. Development and validation of the Ulcerative Colitis patient-reported outcomes signs and symptoms (UC-pro/SS) diary. *J Patient Rep Outcomes*. 2017;2(1):26.

21 Rapport F, Clement C, Seagrove AC, Alrubaiy L, Hutchings HA, Williams JG. Patient views about the impact of ulcerative colitis and its management with drug treatment and surgery: a nested qualitative study within the CONSTRUCT trial. *BMC Gastroenterol*. 2019;19(1):166.

22 Van der Waal MB, Flach J, Browne PD, Besseling-van der Vaart I, Claassen E, van de Burgwal LHM. Probiotics for improving quality of life in ulcerative colitis: Exploring the patient perspective. *PharmaNutrition*. 2019;7:100139.

23 Walsh A, Matini L, Hinds C, et al. Real-time data monitoring for ulcerative colitis: patient perception and qualitative analysis. *Intest Res*. 2019;17(3):365-374.

24 Filgotinib in the induction and maintenance of remission in adults with moderately to severely active ulcerative colitis (SELECTION). Accessed November, 27 2023. <https://clinicaltrials.gov/ct2/show/NCT02914522>

25 Feagan BG, Danese S, Loftus EV, Jr., et al. Filgotinib as induction and maintenance therapy for ulcerative colitis (SELECTION): a phase 2b/3 double-blind, randomised, placebo-controlled trial. *Lancet*. 2021;397(10292):2372-2384.

26 Burmester GR, Nash P, Sands BE, et al. Adverse events of special interest in clinical trials of rheumatoid arthritis, psoriatic arthritis, ulcerative colitis and psoriasis with 37 066 patient-years of tofacitinib exposure. *RMD Open*. 2021;7(2).

27 Zhou Q, Shen ZF, Wu BS, et al. Risk of colorectal cancer in ulcerative colitis patients: a systematic review and meta-analysis. *Gastroenterol Res Pract*. 2019;2019:5363261.

28 Rabbenou W, Ullman TA. Risk of colon cancer and recommended surveillance strategies in patients with ulcerative colitis. *Gastroenterol Clin North Am*. 2020;49(4):791-807.

29 Scoville EA, Konijeti GG, Nguyen DD, Sauk J, Yajnik V, Ananthakrishnan AN. Venous thromboembolism in patients with inflammatory bowel diseases: a case-control study of risk factors. *Inflamm Bowel Dis*. 2014;20(4):631-636.
